# A three-plasmid-containing CRISPR-Cas9 platform to engineer *Bacillus velezensis* 916 as an efficient biocontrol agent

**DOI:** 10.1128/aem.01389-25

**Published:** 2025-09-02

**Authors:** Lian Li, Kecheng Luo, Shuangyu Zhang, Xiaohua Wang, Sihan Wang, Xuehui Liu, Shanshan Zang, Yuan Liu, Changyong Zhou, Chuping Luo

**Affiliations:** 1 Jiangsu Provincial Key Construction Laboratory of Probiotics Preparation, Huaiyin Institute of Technology 66520, Huaian, China; 2 Institute of Biophysics, Chinese Academy of Sciences 53015, Beijing, China; 3 Huaiyin Academy of Agricultural Sciences, Huaian, China; The University of Tennessee Knoxville, Knoxville, Tennessee, USA

**Keywords:** CRISPR-Cas9 platform, *Bacillus velezensis*, cyclic lipopeptides, antagonistic activity, biocontrol efficiency

## Abstract

*Bacillus velezensis* (Bv) is a widely used biocontrol agent against plant diseases, mainly because its genome contains numerous non-ribosomal peptide synthetases (NRPS) gene clusters for the synthesis of various cyclic lipopeptides (CLPs). The domesticated strain Bv916, capable of co-producing four CLPs, has been successfully applied for green control of rice sheath blight and angular leaf spot. To enhance Bv916’s biological control efficacy while maintaining environmental safety, it is essential to establish a food-grade gene editing platform in Bv916. Here, a three-plasmid CRISPR-Cas9 platform for Bv916 was constructed using the thermosensitive origin pET194ts, constitutive P43 promoters for Cas9, the specific promoter Psrf for single guide RNAs (sgRNAs), and three resistance gene expression cassettes. By replacing the native promoters of ComX and RecA in Bv916 with the strong promoters P43 and PrepU, respectively, this platform achieved a single-gene editing efficiency of 96%, while the simultaneous dual-gene editing efficiency reached 61%, with each round completed within five business days. Furthermore, this gene editing platform is used to replace promoters of four NRPS gene clusters (*loc*, *srf*, *bl*, and *fen*) in Bv916 with strong constitutive promoters (PB, PA, P43, and PrepU), generating the derivative BvLSBF. Compared to Bv916, BvLSBF showed 6.8-fold, 5.9-fold, 10.9-fold, and 6.2-fold increases in locillomycin, surfactin, bacillomycin L, and fengycin, respectively. Its antagonistic activity against plant pathogens was also significantly enhanced. This system enables further development of Bv916 as a cell factory and integration of multiple biocontrol factors, offering significant potential for sustainable agriculture.

**IMPORTANCE:**

In this study, a food-grade three-plasmid CRISPR-Cas9 platform for Bv916 was established by incorporating the optimized BvCas9 under the constitutive promoter P43, single guide RNAs (sgRNAs), and homologous recombination fragments into three thermosensitive shuttle vectors. This gene editing system was used to achieve gene insertion, deletion, and replacement in Bv916, particularly by editing four non-ribosomal peptide synthetase (NRPS) gene clusters. This resulted in increased production of four cyclic lipopeptides and significantly enhanced antibacterial and antifungal activity.

## INTRODUCTION

Due to the need for sustainable agricultural systems and the desire for chemical-free foods, intensive research has been conducted into alternatives to chemical control of plant diseases (1–3). Microorganisms, particularly *Bacillus*, are the preferred candidates for the biocontrol of these pathogenic microorganisms, including bacteria, fungi, and nematodes, due to their ability to form endospores, produce a variety of bioactive compounds, and promote plant growth (4, 5). Based on the current pangenomic reassignment and unique phylogenomic features of Bv, several biocontrol strains originally classified under other *Bacillus* species—such as *Bacillus amyloliquefaciens* FZB42^T^, *Bacillus subtilis* QST713, and *Bacillus subtilis* GB03—have been reclassified into the Bv group (4, 6–9). *Bacillus velezensis* (Bv) Bv916, investigated in this study, shares the same reclassification trajectory. The Bv strains exhibit an excellent genetic ability to synthesize secondary metabolites, including cyclic lipopeptides (CLPs) (i.e., surfactin, iturin, fengycin, and locillomycin) and polyketides (i.e., macrolactin, bacillaene, and difficidin), which are produced at the end of the exponential phase by non-ribosomal peptide synthetases (NRPSs) and polyketide synthetases (PKSs) (10–14). In particular, CLPs produced by Bv can induce systemic resistance in plants and inhibit plant pathogens (15, 16). The domesticated Bv916 with high production of four CLPs (surfactin, bacillomycin L, fengycin, and locillomycin) was derived from the wild-type strain through continuous domestication over many years through physical and chemical mutagenesis (9). Due to Bv’s environmental friendliness and probiotic properties, it has recently been increasingly studied and used in the agricultural industry to combat plant diseases (5, 17, 18).

Biocontrol agents developed from Bv and its active products, like other biological pesticides, have inherent disadvantages compared to chemical pesticides, such as a narrow bacteriostatic spectrum, slow action, susceptibility to environmental factors, and poor stability (19–22). The clustered regularly interspaced short palindromic repeats and associated genes (CRISPR-Cas9) system is a revolutionary gene-editing technology capable of precisely and efficiently modifying bacterial genomic DNA. Its broad applications offer an effective solution to the aforementioned challenges (23–29). The fundamental mechanism of CRISPR-Cas9 gene editing relies on the pairing of the Cas9 nuclease with a single guide RNA (sgRNA), which confers high precision and programmability. Here, the Cas9 nuclease functions as a proteolytic enzyme capable of cleaving specific target sites in DNA, while the sgRNA directs Cas9 to the intended genomic locus through complementary binding, thereby guiding site-specific DNA cleavage (30, 31). In the widely used Class II CRISPR-Cas9 system for bacterial genome editing, three key elements are typically involved: (i) sgRNA, which is a chimera of CRISPR-RNA (crRNA), and transactivating CRISPR-RNA (tracrRNA) containing a 20-nt target-specific sequence that matches the target genomic DNA sequence and includes a specific Protospacer Adjacent Motif (PAM); (ii) Cas9, an sgRNA-directed endonuclease that creates a double-strand break (DSB) at the target genomic DNA; (iii) a donor DNA template for precise DSB repair via homologous recombination (32, 33). Alternatively, the DSBs can also be repaired via the non-homologous end-joining pathway. However, this results in small insertions or deletions at the target site if a donor DNA template is not available for repair (34).

*Bacillus subtilis*, a typical Gram-positive model strain, has achieved significant breakthroughs in the development of its CRISPR-Cas9 system. Currently, three typical CRISPR-Cas9 system architectures have been established for this strain, including all-in-one, double plasmid, and chromosomally integrated systems. Since Jiang et al. (35) first reported that a strategy based on the CRISPR-Cas9 system was developed for continuous genome editing in the Gram-negative bacteria *Escherichia coli* and *Tatumella citrea*, numerous CRISPR-Cas9 platforms have been developed for the Gram-positive bacteria *Bacillus* sp. Genome iterative editions have been developed and used for large genomic deletion, gene insertion, and point mutation (26, 36–38). However, compared to the development of CRISPR-Cas9 gene editing toolkits in *B. subtilis*, *Bacillus anthracis*, *Bacillus cereus*, *Bacillus licheniformis,* and *Bacillus mycoides* et al., there are only a few reports on Bv (26, 39). Given this gap, the development of efficient and high-precision CRISPR-Cas9 platforms for Bv is imminent and is crucial for its further use in sustainable agriculture. The genome editing efficiency of these CRISPR-Cas9 systems was further optimized by screening appropriate constitutive promoters, inducible promoters, or growth-phase-specific promoters to establish Cas9 and sgRNA-specific expression patterns in *B. subtilis* (26, 36, 40–42). However, all three CRISPR-Cas9 systems require curing the plasmid containing the previous sgRNA and introducing a new plasmid containing the new sgRNA to achieve multi-step genome editing in *B. subtilis*. Currently, three main methods are used to eliminate plasmids in *B. subtilis*: temperature-sensitive replication, traditional negative selection, and programmed sgRNA removal systems. However, all of these methods are time-consuming and labor-intensive (26). Therefore, the above research has laid a solid foundation for the development of a CRISPR-Cas9 platform that can efficiently edit the genome of Bv.

In the study, we focused on engineering Bv916 as an efficient bio-control agent using the constructed three-plasmid containing CRISPR-Cas9 platform, and testing its control effect through antibacterial activity and biological control experiments.

## MATERIALS AND METHODS

### Strains and media

The plasmids and strains used in this study are listed in Table 1. Luria-Bertani broth (LB) medium (10 g/L tryptone, 5 g/L yeast extract, 5 g/L NaCl) was used for routine culture of *E. coli*, Bv, and other bacterial strains. The potato dextrose agar (PDA) medium (200 g/L potato infusion, 20 g/L glucose, 20 g/L agar) was used to cultivate fungi. As previously reported, GCHE (0.2% potassium L-glutamate, 100 mmoL/L potassium phosphate buffer [pH 7], 3 mmoL/L trisodium citrate, 3 mmoL/L MgSO_4_·7H_2_O, 22 mg/L ferric ammonium citrate, 50 mg/L L-tryptophan, 0.1% casein hydrolysate, 1% glucose) and GE (GCHE medium without casein hydrolysate) medium were used for the transformation of Bv strains (43). Difco sporulation medium (DSM, 0.8% Difco nutrient broth, 0.025% MgSO_4_·7H_2_O, 0.1% KCl, 10 µmoL/L MnCL_2_, 1 µmoL/L FeSO_4_, and 1 mmoL/L Ca[NO_3_]_2_) was used to induce sporulation of Bv by nutrient depletion (44). All media were adjusted to pH 7.0. When necessary, antibiotics were added at appropriate screening concentrations: erythromycin 1 µg/mL, chloramphenicol 5 µg/mL, neomycin 20 µg/mL, kanamycin 20 µg/mL, ampicillin 100 µg/mL, and spectinomycin 100 µg/mL.

**TABLE 1:** *B. velezensis* strains and plasmids used

Strains or plasmids	Description^*a*^	Source or reference
Strains		
*Bacillus velezensis* 916 (Bv916)	Wild type, co-production of surfactin, fengycin, bacillomycin L, and locillomycin, spore formation	CGMCC No. 0808
BvCotB-GFP	Bv916 derivative, the putative coat protein BvCotB tagged by GFP	This study
BvCgeA-RFP	Bv916 derivative, the putative coat protein BvCgeA tagged by RFP	This study
Bv-GFP-RFP	Bv916 derivative, the putative coat protein BvCotB and BvCgeA tagged by GFP and RFP, respectively	This study
Bv-srf-loc	Bv916 derivative, the native promoters of surfactin and locillomycin were substituted by PA (from *Bacillus subtilis* regulatory gene *rsbU*) and PB (from *Bacillus subtilis* positive regulator *rsbV*) promoters, respectively	This study
Bv-bl-fen	Bv916 derivative, the native promoters of bacillomycin L and fengycin were substituted by P43 (from *Bacillus subtilis* JCL16) and PrepU (from *Staphylococcus aureus* plasmid pUB110) promoters, respectively	This study
BvLSBF	Bv916 derivative, the native promoters of surfactin, locillomycin, bacillomycin L, and fengycin were substituted by PA, PB, P43, and PrepU promoters, respectively	This study
△ComX	Bv916 derivative, the native promoters of ComX were substituted by P43 promoters	This study
△RecA	Bv916 derivative, the native promoters of RecA were substituted by PrepU promoters	This study
△ComX△RecA	Bv916 derivative, the native promoters of ComX and RecA were substituted by P43 and PrepU promoters, respectively	This study
Bv916-GFP	Bv916 derivative, Bv916 tagged with green fluorescent protein	This study
Bv-srf-loc-GFP	Bv916 derivative, Bv-srf-loc tagged with green fluorescent protein	This study
Bv-bl-fen-GFP	Bv916 derivative, Bv-bl-fen tagged with green fluorescent protein	This study
BvLSBF-GFP	Bv916 derivative, BvLSBF tagged with green fluorescent protein	This study
Plasmids		
pUC19	Cloning vector, Amp^r^	Laboratory stock
pMarA	TnYLB-1 delivery plasmids, pET194ts origin of replication, Kan^r^, Em^r^	BGSC
pMUTIN4	Integrated vector, Amp^r^, Em^r^	BGSC
pBEST501	Integrated vector, Neo^r^	BGSC
pRp22-gfp	Cl^r^	Laboratory stock
pTN-Cas9	Expression vector, pTN carrying neomycin cassette from pBEST501, native surfactin promoter Psrf and T1T2 terminator, and pET194ts origin of replication of pMarA, Neo^r^	This study
pTC-CgeA-RFP	pTarget plasmid, containing P43 promoter fusion specific sgRNA1, and coat protein BvCgeA gene fusion with RFP for homologous recombination, Cl^r^	This study
pTK-CotB-GFP	pTarget plasmid, containing P43 promoter fusion with specific sgRNA2, and coat protein BvCotB gene fusion with GFP for homologous recombination, Kan^r^	This study
pTC-PA-srf	pTarget plasmid, containing P43 promoter fusion with specific sgRNA3, and a sandwich with two homologous fragments and PA promoter, Cl^r^	This study
pTK-PB-loc	pTarget plasmid, containing P43 promoter fusion with specific sgRNA4, and a sandwich with two homologous fragments and PB promoter, Kan^r^	This study
pTK-P43-bl	pTarget plasmid, containing P43 promoter fusion with specific sgRNA5, and a sandwich with two homologous fragments and P43 promoter, Kan^r^	This study
pTC-PrepU-fen	pTarget plasmid, containing P43 promoter fusion with specific sgRNA6, and a sandwich with two homologous fragments and PrepU promoter, Cl^r^	This study
pTK-comX	pTarget plasmid, containing P43 promoter fusion with specific sgRNA7, and a sandwich with two homologous fragments and P43 promoter, Kan^r^	This study
pTC-recA	pTarget plasmid, containing P43 promoter fusion with specific sgRNA8, and a sandwich with two homologous fragments and PrepU promoter, Cl^r^	This study

^*a*^ Amp^r^, ampicillin resistance; Em^r^, erythromycin resistance; Cl^r^, chloramphenicol resistance; SpeC^r^, spectinomycin resistance; Kan^r^, kanamycin resistance; Neo^r^, neomycin resistance; BGSC, *Bacillus* Genetic Stock Center.

### Construction of recombinant plasmids

The nucleotide sequence for Cas9 was optimized according to the codon preference of Bv916. The expression cassette that fused the native promoter for surfactin (Psrf), the designed gene for Cas9, and the phage-derived transcription terminator T_1_T_2_ was fully synthesized by chemical synthesis (Bioengineering Co., Ltd., Shanghai). The synthetic expression cassette was inserted into the double digestion sites of *Apa* I and *Xho* I of the shuttle plasmid vector pTN, resulting in the recombinant expression vector pTN-Cas9 for heterologous expression of Cas9 in Bv. The nucleotide sequences of *BvCotB* and *BvCgeA* in Bv916, which are highly homologous to the nucleotide sequence of the spore proteins CotB and CgeA of *B. subtilis* 168, were identified by DNAMAN software. Based on the nucleotide sequences of *BvCotB* and *BvCgeA*, sgRNA1 and sgRNA2 sequences were designed, and transcription boxes P43sgRNA1 and P43sgRNA2 were constructed by fusing P43 promoters with sgRNA1 and sgRNA2, respectively (also synthesized by Bioengineering Co., Ltd., Shanghai). The transcription boxes P43sgRNA1 and P43sgRNA2 were inserted into pTK (double digested by *Bam*H I and *Eco*R I) and pTC (double digested by *Bam*H I and *Xba* I), respectively, resulting in the plasmids pTK-sgRNA1 and pTC-sgRNA2. Three primer pairs, COTBUF2/COTBUR2, COTBDF2/COTBDR2, and COBGFPF2/COBGFPR2 (Table 2), were used to amplify the upstream sequence of the *BvCotB* gene, the green fluorescent protein (GFP) sequence, and the downstream sequence of the *BvCotB* gene, respectively. Subsequently, the sandwich GFP expression cassette, in which the GFP gene was fused to the upstream and downstream genes of *BvCotB,* was obtained by overlapping PCR. Finally, the sandwich GFP expression cassette was double-digested and inserted into the also double-digested plasmid pTK-sgRNA1, resulting in the recombinant plasmid pTK-CotB-GFP. In the same way, the sandwich red fluorescent protein (RFP) expression cassette, in which the RFP gene was fused with the upstream and downstream genes of BvCgeA, was constructed by three primer pairs CGEAUF2/CGEAUR2, CGEARFPF2/CGEARFPR2, CGEADF2/CGEADR2, and converted into pTC-sgRNA2 was inserted, resulting in the recombinant plasmid pTC-CgeA-RFP. The construction of other recombinant plasmids, including pTC-PA-srf, pTK-PB-loc, pTK-P43-bl, pTC-PrepU-fen, pTK-comX, and pTC-recA, was very similar to that of pTK-CotB-GFP and pTC-CgeA-RFP (Fig. S1 to S9). Their detailed gene sequences are shown in Fig. S10 to S18.

**TABLE 2:** Primers used in this study

Primer	Sequence (5′–3′)	Note
COTBUF2	5′-TTTGAATTCTGATCAGCCGGATGACCAAT-3′	Used for sandwich homologous recombination fragment CotBUp-GFP-CotB Down construction
COTBUR2	5′-CTTGCCCCCTCCGCCACCTTTACGGCCGTGTTTCCACC-3′	
COTBGFPF2	5′-GGTGGCGGAGGGGGCAAGATGGTGAGCAAGGGCGAGG -3’	
COTBGFPR2	5′-GTTTACGGACAATCTTTCCAATTACTTGTACAGCTCGTCCA-3′	
COTBDF2	5′-TTGGAAAGATTGTCCGTAAAC-3′	
COTBDR2	5′-TTTCTCGAGCTAAATCCAGAATAATCAGCTT-3′	
CGEAUF2	5′-TTTTCTAGACTCCTTCATCTTTTAATCTA-3′	Used for sandwich homologous recombination fragment CgeAUp-RFP-CgeA Down construction
CGEAUR2	5′-CTTGCCCCCTCCGCCACCCGAAGTGAACGTGACACTTT-3′	
CGEARFPF2	5′-TATAAAACGTTCATATGCTGTTACAGGAACAGGTGGTGGC-3′	
CGEARFPR2	5′-CAGCATATGAACGTTTTATA-3’	
CGEADF2	5′-TTTCTCGAGTTCGGGTGCAATCCATTCAT-3′	
CGEADR2	5′-TTTCTCGAGTTCGGGTGCAATCCATTCAT-3′	

### Preparation of competent cells and transformation

The preparation of competent cells and the transformation of Bv916 were carried out according to the method previously developed by Kunst F and Rapoport G with minor modifications (43). Preparation of competent cells: a single colony of Bv916 from an LB plate was inoculated into 5 mL of GCHE liquid medium and cultured at 37°C with shaking at 160 rpm for 13–15 h. Then, 1 mL of the Bv916 culture was inoculated into 24 mL of GCHE liquid medium and cultured at 28°C and 180 rpm for approximately 3 h until the OD_600_ reached 0.6–0.8. Next, 25 mL of GE medium was added to the culture, followed by further incubation at 37°C with shaking at 200 rpm for 1 h. The culture was aliquoted into 2 mL centrifuge tubes, centrifuged at 5,000 rpm for 5 min, and the supernatant was partially discarded. The pellet was resuspended in the remaining 1 mL supernatant, dispensed into 100 µL aliquots, and stored at −80°C. For transformation, the competent cells prepared above were taken out (thawed on ice bath if previously stored at −80°C), about 1–10 µg of recombinant plasmid was added, and left to stand for 25 min. The mixture was then cultured at 37°C with shaking at 100 rpm for 0.5 h, then 500 µL of GCHE medium was added and incubation continued for 1 h. Subsequently, the cells were spread on LB plates containing the corresponding antibiotics to obtain transformants. The positive transformants were further confirmed by PCR.

### Engineering the genome of Bv916 using the CRISPR-Cas9 platform

The constructed plasmids pTK-CotB-GFP and/or pTC-CgeA-RFP were co-transformed with plasmid pTN-Cas9 into Bv916, yielding single-fluorescent derivative BvGFP or BvRFP and double-fluorescent derivative Bv-GFP-RFP. The gene editing efficiency of the three-plasmid CRISPR-Cas9 platform was evaluated by analyzing the ratio of single fluorescent colonies, double fluorescent colonies, and total transformed colonies. Three plasmids, pTC-PA-srf, pTK-PB-loc, and pTN-Cas9, were co-transformed into Bv916 and gave rise to the derivative Bv-srf-loc. Similarly, three plasmids, pTK-P43-bl, pTC-PrepU-fen, and pTN-Cas9, were co-transformed into Bv916 and gave rise to the derivative Bv-bl-fen. Three plasmids, pTK-P43-bl, pTC-PrepU-fen, and pTN-Cas9, were co-transformed into Bv-srf-loc and gave rise to the derivative BvLSBF. All derived strains were further verified by PCR. The plasmids in Bv916 and its derivatives were eliminated by high-temperature treatment at 50°C, as all plasmids used in the CRISPR-Cas9 platform contained the temperature-sensitive replication origin.

For strains integrated with GFP and/or RFP, colonies on the plates can be examined under UV light to check for red and/or green fluorescence, and the relevant efficiencies can be calculated.

Transformation efficiency (%) = number of transformants/number of competent cells (~10^5^ CFU).

Total editing efficiency (%) = number of fluorescent colonies/number of transformants.

### The addition of GFP tags in Bv916 derivatives

Transformation of plasmid pRp22-gfp (45) into Bv916 derivatives Bv-srf-loc, Bv-bl-fen, and BvLSBF yielded the corresponding GFP-tagged strains: Bv916-GFP, Bv-srf-loc-GFP, Bv-bl-fen-GFP, and BvLSBF-GFP on LB plates supplemented with chloramphenicol.

### Spore formation and purification

A single colony of Bv916 and its derivatives, grown on LB agar plates at 37°C for approximately 16 h, was inoculated into 2 mL of DSM medium in each tube, followed by overnight cultivation at 37°C with shaking at 180 rpm. Then the cultures were transferred into a 100 mL Erlenmeyer flask containing 25 mL of DSM medium prewarmed at 37°C, and the culture was further shaken at 180 rpm for approximately 48 h. When the spores in the culture reach more than 90% according to microscopic observation, the cells (crude spores) were collected by centrifugation (8,000 rpm) at 4°C and stored at –20°C. The crude spores were further purified as follows. The crude spore collections were rinsed three times with 10 mL of 0.5 mol/L NaCl and suspended in Tris-HCl buffer (50 mmol/L, pH 7.2). Then, the samples were treated with 50 µg/mL lysozyme at 37°C for 1 h. After centrifugation, the cells were suspended in 1 mL of sterile water containing the purified spores and stored at –20°C.

### Microscope assay

Ten microliters of purified spore samples was transferred onto microscope slides and air-dried naturally (appropriate dilution was performed if the spore concentration was too high). Spore proteins fused with GFP or RFP were observed using confocal laser microscopy (product no. LSM880, ZEISS, Germany) with a 63× oil-immersion objective. The exposure time for acquiring images of the GFP fusion was 0.5 to 1 s, while for the RFP fusion, it was 1.5 to 2 s. Images were processed using ZEN 3.4 (Blue Edition), including overlay, contrast, and tonal balance adjustments.

### Western blot analysis

To confirm the successful insertion of recombinant spores in the engineered strain, western blot (WB) analysis of the recombinant spores was performed. The prepared spore suspension was centrifuged at 8,000 rpm for 10 min. An appropriate volume of SDS-DTT solution was added, followed by incubation in a water bath at 37°C for 2 h. After centrifugation at 8,000 rpm (4°C) for 10 min, the pellet was washed three times with 1 mol/L Tris-HCl buffer (pH 8.0). Lysis buffer was added, and the sample was sonicated on ice until the solution became clear. Following another centrifugation at 8,000 rpm (4°C) for 10 min, 5× loading buffer was added, and proteins were denatured at 100°C for 10 min. SDS-PAGE was performed using a 12.5% separating gel and 5% stacking gel. After loading the boiled samples, electrophoresis was initiated at 90 V until the samples entered the separating gel, then increased to 120 V until the dye front reached the bottom. For protein transfer, filter papers and NC membrane were pre-wetted in transfer buffer. Using the sandwich method, the gel was placed between the filter paper and the NC membrane, and the bubbles were removed carefully using a roller. Semi-dry transfer was conducted for 15 min. The membrane was blocked with 5% skim milk for 2 h at room temperature, followed by three 10 min washes with PBST. Primary antibodies (anti-GFP mouse monoclonal, 1:2,000; anti-RFP mouse monoclonal, 1:8,000) diluted in PBS were incubated at 4°C overnight or 2 h at room temperature. After PBST washes, HRP-conjugated goat anti-mouse IgG secondary antibody (1:10,000) was applied for 1 h at room temperature. Following final washes, chemiluminescent substrate was added for detection.

To further confirm the successful insertion of the Cas9 protein in the recombinant strain, Cas9 protein WB analysis was performed. BvGFP and BvRFP recombinant strains were cultured in LB medium with appropriate antibiotics at 37°C, 200 rpm for 12 h. Cells were collected by centrifugation at 12,000 rpm for 1 min and treated with 4 mg/mL lysozyme at 37°C for 1 h. After washing with PBS, cells were resuspended and sonicated on ice. Subsequent steps followed the recombinant spore WB protocol. Primary antibody was GenScript Cas9 rabbit antibody (1:2,000), with HRP-conjugated goat anti-rabbit IgG (1:10,000) as secondary antibody.

### HPLC-MS analysis of cyclic lipopeptides

Isolation and high-performance liquid chromatography-mass spectrometry (HPLC-MS) analysis of the four groups of CLPs—surfactin, bacillomycin L, fengycin, and locillomycin—produced by Bv916 and its derivatives (Bv-srf-loc, Bv-bl-fen, and BvLSBF), were performed according to our previous report (13). Briefly, Bv916 and its derivative strains were inoculated into LB liquid medium and cultured at 28°C with shaking (180 rpm) for 72 h. After removing bacterial cells by centrifugation, the supernatant pH was adjusted to 2.8 to precipitate cyclic lipopeptides. The CLP-containing pellet was collected by supernatant removal, dried, and extracted with methanol. Before HPLC-MS analysis, the methanol extractions were treated with activated carbon and passed through a 0.22 µM pore filter. The liquid chromatography analysis was performed using a C18 column (5 µm; 250 × 4.6 mm; VYDAC 218 TP; VYDAC, Hesperia, CA) for all detection conditions. The four CLPs were further purified using the established method. The four CLP groups were analyzed by HPLC using the acetonitrile-water-trifluoroacetic acid solvent system (80:20:0.5 [vol/vol/vol] for surfactin, 50:50:0.5 [vol/vol/vol] for locillomycin and fengycin, and 40:60:0.5 [vol/vol/vol] for bacillomycin L) at a rate of 0.5 mL/min. Locillomycin was monitored at 230 nm, and the other three CLPs were monitored at 210 nm. Using surfactin purchased from Sigma and previously prepared and verified bacillomycin L, fengycin, and locillomycin as standards to calibrate the HPLC quantitative data. All methanol extractions were further analyzed by matrix-assisted laser desorption ionization time-of-flight mass spectrometry (MS) using an Agilent 1100 series HPLC-MS system.

### Antibiotic susceptibility test

The susceptibility of Bv916 and its derivatives (Bv-srf-loc, Bv-bl-fen, and BvLSBF) to four antibiotics (Ampicillin, Kanamycin, Erythromycin, and Chloramphenicol) was evaluated using the disk diffusion method. Briefly, 0.1 mL of bacterial cultures of Bv916 and its derivatives, grown in LB liquid medium for 16 h, were evenly spread onto LB solid agar plates. Antibiotic disks were then aseptically placed on the surface of the agar plates, followed by incubation at 37°C for 24 h. After incubation, the susceptibility of Bv916 and its derivatives was analyzed by measuring the diameter (mm) of the inhibition zones.

### Assessment of antimicrobial activity

The antifungal activities of wild-type Bv916 and its derivatives (Bv-srf-loc, Bv-bl-fen, and BvLSBF) against *Fusarium oxysporum, Rhizoctonia solani* et al. were carried out as follows. Before testing, all the fungi were grown on PDA medium. Mycelial plugs (5 mm) were placed at the center of the LB plates. Two microliter culture broth samples of Bv916 and its derivatives (Bv-srf-loc, Bv-bl-fen, BvLSBF), prepared by 16 h cultivation in LB liquid medium, were evenly spotted at positions 2.5 cm from the edge of the mycelial plugs at equal intervals. Each treatment was performed in triplicate. The plates were sealed with parafilm and incubated at 28°C. The zones of inhibition were measured after 24 to 72 h. The antifungal activity changes of the derivatives were evaluated with Bv916 as the control.

The antibacterial activity of Bv916 and its derivatives was evaluated as follows. When the OD_600_ of *Staphylococcus aureus* cultured in LB liquid medium reached 0.8, the cultures were diluted 1:100 in LB medium solidified with 1.5% agar, mixed thoroughly, and poured into plates. After solidification, a sterilized Oxford cup was gently placed vertically at the center of the LB agar plate using sterile forceps. Single colonies of Bv916 and its derivatives were inoculated into LB liquid medium and cultured at 37°C with shaking (180 rpm) for 48 h. After centrifugation of the fermentation broth, 200 µL of supernatant was aliquoted into Oxford cups. Each treatment was performed in triplicate. The plates were incubated at 37°C for 24 h, and the diameters of inhibition zones surrounding the Oxford cups were measured. The net inhibition zone diameter (mm) = (total zone diameter) − (Oxford cup diameter).

### Biocontrol assays of wild-type Bv916 and its derivatives against rice sheath blight and bacterial colonization in rice plants

Fifty milliliters of sterile PDA medium with 100 wooden matchsticks in 250 mL Erlenmeyer flasks was inoculated with a 5 mm plug taken from a PDA Petri dish culture of *R. solani*. The flasks were incubated without shaking at 30°C for 1 week. Rice cultivar 9311 seeds were soaked in water for 48 h and then sown in the nursery containing sterile organic soil. The 28-day-old seedlings were then transplanted into the 15 cm × 30 cm pots, also containing sterile organic soil. To simulate its natural environment, the rice seedlings in the pots were grown outdoors. During the joint-booting stage, each rice plant was inoculated with 10 matchsticks of *R. solani* between the stems and leaves (i.e., rice sheath). Then rice plants were sprayed with 20 mL of Bv916-GFP, Bv-bl-fen-GFP, Bv-srf-loc-GFP, or BvLSBF-GFP cultures, while control plants received 20 mL of water. From day 0 to 15 post-inoculation, rice leaves colonized by Bv916 and its derivatives were daily excised and observed under a confocal microscope at 40× magnification to examine colonization efficiency, with images captured accordingly. Disease severity was assessed from 3 to 20 days post-inoculation, with eight replicate pots per treatment.

The biocontrol efficacy was calculated using the following formula:

Biocontrol efficacy (%) = [(lesion length/plant height in control − lesion length/plant height in treatment)/(lesion length/plant height in control)] × 100%.

### Biocontrol assays for wild-type Bv916 and its derivatives against angular leaf spot

The experiment was conducted in greenhouse trial fields at the Huaian Academy of Agricultural Sciences in Huaian City, Jiangsu Province. A field with a history of continuous melon cultivation for over 2 years and severe recurring bacterial angular leaf spot was selected for the study. The experiment included five treatments: Bv916, Bv-bl-fen, Bv-srf-loc, BvLSBF fermentation broth, and a water control (CK). Each treatment was applied at a rate of 0.5 L/m², with three replicates per treatment, resulting in a total of 15 plots. Each plot measured approximately 3 × 6 m and was arranged in a randomized complete block design. Isolation rows were established between plots, and protective rows were set up along the edges.

Disease assessment was conducted 7 days after each application. Five sampling points were randomly selected per plot, with 10 leaves examined per point. Disease severity was graded based on the percentage of leaf area covered by lesions, using the following scale. 0: No lesions; 1: lesions covering ≤5% of the leaf area; 3: lesions covering 6%–10% of the leaf area; 5: lesions covering 11%–20% of the leaf area; 7: lesions covering 21%–50% of the leaf area; 9: lesions covering >51% of the leaf area.

The disease index and biocontrol efficacy were calculated as follows:

Disease index = ∑(number of diseased leaves at each grade × grade value)/(total leaves investigated × highest grade value) × 100.

Biocontrol efficacy (%) = [(disease index of control − disease index of treatment)/disease index of control] × 100.

### Statistical analysis

Data analyses and column chart drawings were conducted using Microsoft Excel 2010 software and GraphPad Prism 9 statistical software, respectively. Experimental data in each group were analyzed by one-way analysis of variance (ANOVA) or two-way ANOVA. All data were presented as the mean  ±  standard deviation from at least three independent experiments.

## RESULTS

### Development of a three-plasmid CRISPR-Cas9 platform for Bv916

**Fig 1:**
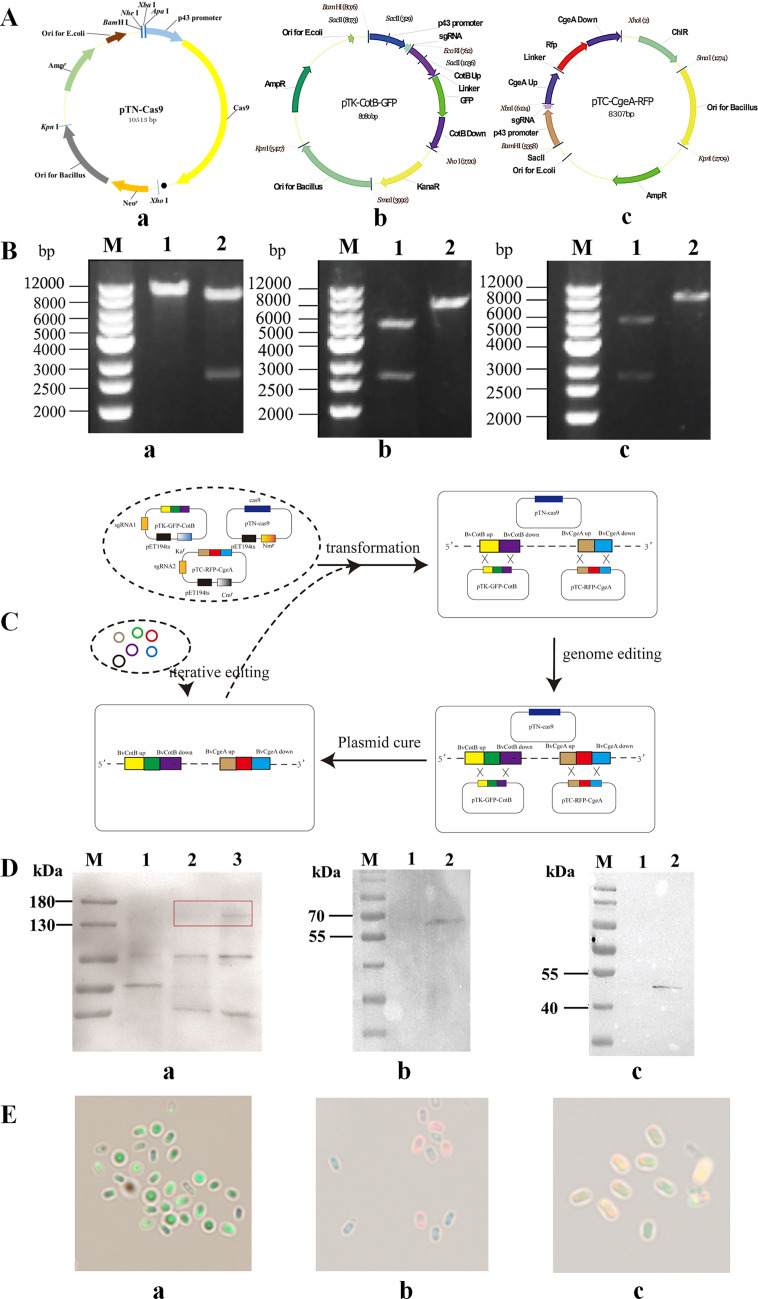
Construction of the three-plasmid containing CRISPR-Cas9 platform for tandem and iterative genome engineering. (**A**) Maps of the plasmid pTN-Cas9 (Aa) containing the Cas9 expression cassette that fused the native surfactin promoter (Psrf), the optimized gene for Cas9 in Bv916, and the transcription terminator T_1_T_2_. Maps of pTK-CotB-GFP (Ab), which contains the BvCotB sgRNA transcription module and the donor DNA template for homologous recombination of the Bv*CotB-GFP* fusion gene. Maps of Bv (Ac), which contain the BvCgeA sgRNA transcription and the donor DNA template for homologous recombination of the BvcgeA*-*RFP fusion gene. All plasmids contained temperature-sensitive replication origin pET194ts derived from pMarA and were cured at 50°C. (**B**) Plasmid restriction enzyme verification. (Ba) pTN-Cas9. M: 1 kb DNA Ladder; 1: *Xho* I single digestion; 2: *Xho* I/*Kpn* I double digestion. (Bb) pTK-GFP-CotB, (Bc) pTC-RFP-CgeA. M: 1 kb DNA Ladder; 1: *Bam*H I/*Xho* I double digestion; 2: *Xho* I single digestion. (**C**) Detailed diagram of tandem and iterative genome editing of Bv916 using the three-plasmid containing CRISPR-Cas9 platform. (**D**) Western blot analysis. (Da) Cas9 protein detection by western blot. M: Protein molecular weight marker; 1: Bv916; 2: BvCgeA-RFP; 3: BvCotB-GFP. (Db) Western blot analysis of BvCotB-GFP. M: Protein molecular weight marker; 1: Bv916; 2: BvCotB-GFP. (Dc) Western blot analysis of BvCgeA-RFP. M: Protein molecular weight marker; 1: Bv916; 2: BvCgeA-RFP. (**E**) Fluorescence observation of BvCotB-GFP and BvcgeA-RFP fusions. The BvCotB-GFP, BvCgeA-RFP, and Bv-GFP-RFP strains were induced to sporulate by nutrient starvation. Shown are GFP fluorescence images (Ea), RFP fluorescence images (Eb), and merged GFP and RFP images (Ec).

Based on the initial design, the codon-optimized Cas9 gene was inserted into the shuttle vector pTN, which successfully constructed the pTN-Cas9 vector (Fig. 1Aa and Ba; Fig. S10). The recombinant fragments CotB-RFP and CgeA-RFP were separately inserted into the shuttle vectors pTK and pTC, yielding pTK-CotB-GFP (Fig. 1Ab and Bb; Fig. S11) and pTC-CgeA-RFP (Fig. 1Ac and Bc; Fig. S12), respectively. These verified plasmids (pTK-CotB-GFP or pTC-CgeA-RFP) were then co-transformed with pTN-Cas9 into Bv916, generating the derivatives BvCotB-GFP and BvCotB-RFP (the operation flowchart is shown in Fig. 1C). WB analysis confirmed the expression of a 151 kDa Cas9 protein in both derivatives (Fig. 1Da). Additionally, BvCotB-GFP produced a 60 kDa CotB-GFP fusion protein (Fig. 1Db), while BvCotB-RFP exhibited a 42 kDa CgeA-RFP band (Fig. 1Dc), consistent with predictions. Fluorescence microscopy further validated the successful surface display of GFP (Fig. 1Ea) and RFP (Fig. 1Eb) on Bv916 spores. Similarly, pTK-CotB-GFP, pTC-CgeA-RFP, and pTN-Cas9 were co-transformed into Bv916, and two fluorescence signals were simultaneously observed under fluorescence microscopy (Fig. 1Ec). These results further confirm the successful construction of the three-plasmid CRISPR-Cas9 platform for Bv916.

### Efficiency of genome editing of Bv916 by developed CRISPR-Cas9 platforms

Efficiency of the gene-editing platform was calculated using the aforementioned formula, with results shown in Table 3. For every 10 µg of plasmid transformed into 10⁵ competent cells, the transformation efficiencies reached 0.05% and 0.03% for two-plasmid and three-plasmid, respectively. Under UV light, the number of luminescent colonies was observed, and the editing efficiency was calculated. The single-gene and dual-gene editing efficiencies achieved 80% and 20%, respectively. After 12 h of treatment at 50°C, the efficiency of simultaneous elimination of two plasmids and three plasmids in the transformants was 55% and 26%, respectively. As shown in Fig. 1C, the transformants that eliminate plasmids can enter the next round of double-plasmid or triple-plasmid genome editing. In summary, a three-plasmid gene editing platform was successfully constructed in Bv916 using a thermosensitive replication origin, appropriate promoters, and different expression cassettes for resistance genes. This platform can edit two genes at the same time and complete a cycle within 5 working days, significantly improving editing efficiency compared to previous single-plasmid and double-plasmid gene editing systems. Although this platform enables simultaneous editing of two genes, its editing efficiency still needs improvement compared to reported data (46).

**TABLE 3:** Efficiency of the genome editing of Bv916 and its derivatives (△ComX, △RecA, △ComX△RecA)^*a*^

Recipient strains	Gene editing types	Transformants				Transformation efficiency (%)	Total editing efficiency (%)
		Total number	Green number	Red number	Green and red (editing efficiency)		
Bv916	Two-plasmid type 1	50 ± 8	43 ± 5	–	–	0.05	86
	Two-plasmid type 2	52 ± 9	–	42 ± 5	–	0.05	81
	Three-plasmid type	25 ± 4	9 ± 3	8 ± 3	5 ± 2 (20%)	0.03	88
△ComX	Two-plasmid type 1	202 ± 20	175 ± 18	–	–	0.20	87
	Two-plasmid type 2	206 ± 21	–	170 ± 17	–	0.21	83
	Three-plasmid type	107 ± 11	38 ± 5	34 ± 5	23 ± 4 (21%)	0.11	89
△RecA	Two-plasmid type 1	52 ± 8	48 ± 6	–	–	0.50	92
	Two-plasmid type 2	54 ± 10	–	49 ± 6	–	0.50	91
	Three-plasmid type	27 ± 4	6 ± 2	4 ± 2	15 ± 4 (56%)	0.03	93
△ComX△RecA	Two-plasmid type 1	205 ± 20	195 ± 15	–	–	0.21	95
	Two-plasmid type 2	211 ± 21	–	197 ± 20	–	0.21	93
	Three-plasmid type	112 ± 12	21 ± 4	19 ± 3	68 ± 13 (61%)	0.11	96

^*a*^ Two-plasmid type 1: pTN-Cas9, pTK-CotB-GFP; two-plasmid type 2: pTN-Cas9, pTC-CgeA-RFP; three-plasmid type: pTN-Cas9, pTK-CotB–GFP, pTC-CgeA-RFP; –, not detected.

**Fig 2:**
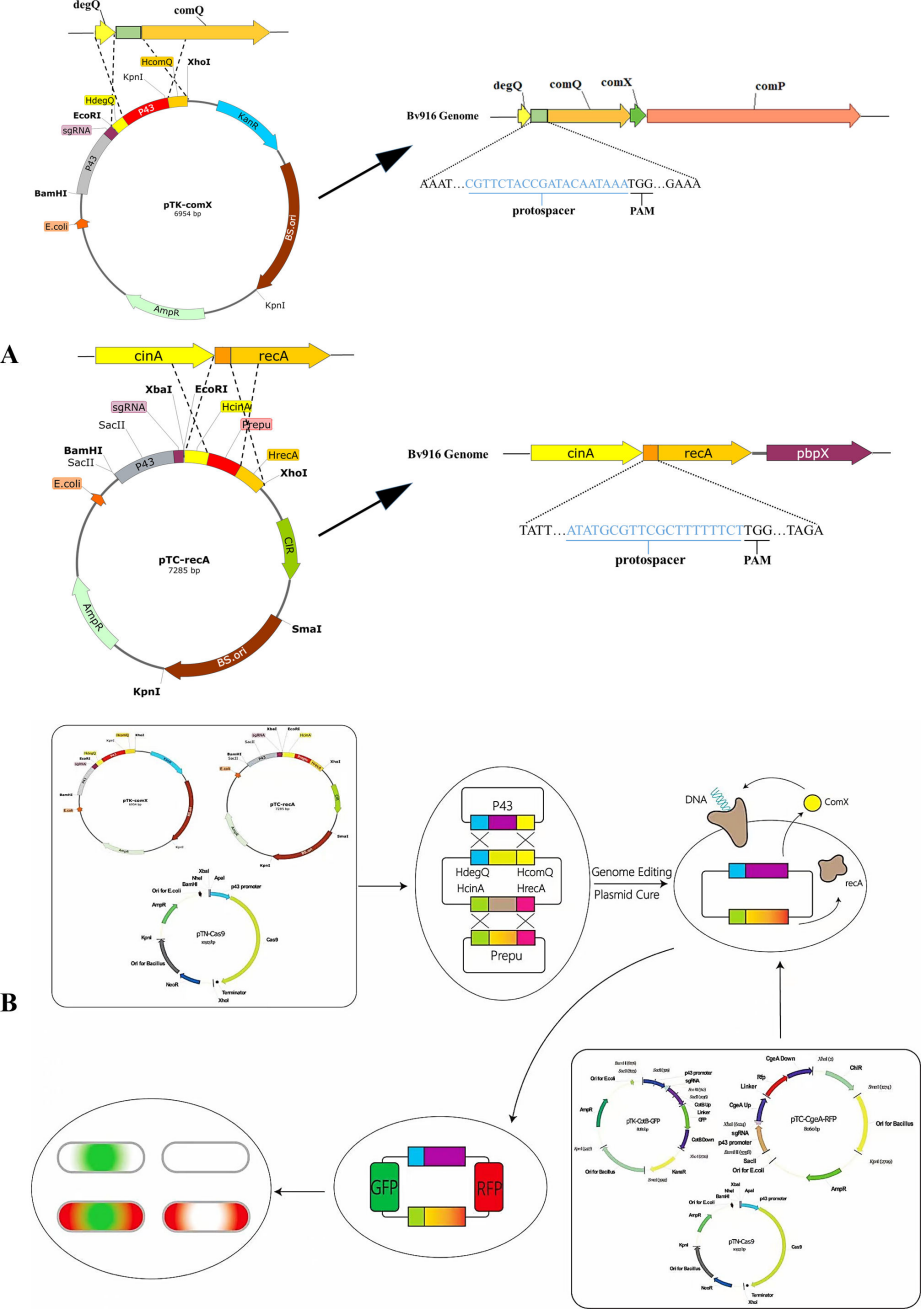
(**A**) Schematic strategy for replacing the native promoters of comX and recA with strong promoters P43 and PrepU. (**B**) Schematic diagram illustrating the principle and experimental procedure for replacing the native promoters of comX and recA in Bv916.

To further enhance the transformation and recombination efficiency of this gene editing platform, plasmids pTK-comX and/or pTK-comX were co-transformed with pTN-Cas9 into Bv916, yielding the derivative strains ΔComX, ΔRecA, and ΔComXΔRecA. This achieved the replacement of the native promoters of ComX and RecA with the strong promoters P43 and PrepU, respectively (Fig. 2A), thus achieving high expression of both ComX and RecA in the Bv916. Subsequently, the three derivative strains were used as recipients for transformation with pTN-Cas9 paired separately with pTK-GFP-CotB or pTC-RFP-CgeA, or co-transformed with all three plasmids, to evaluate the genome editing efficiency of the three-plasmid CRISPR-Cas9 system (Fig. 2B). The results demonstrated that ComX, an essential factor for competence development, increased transformation efficiency by approximately fourfold under high-expression conditions compared to Bv916, yet its recombination efficiency remained unchanged. In stark contrast, overexpression of RecA, a key recombinase in Bv916, elevated recombination efficiency to 90%, while this manipulation had no significant effect on transformation efficiency. Notably, simultaneous overexpression of ComX and RecA synergistically enhanced both transformation and recombination efficiencies. The single-gene editing efficiency reached 96%, and simultaneous dual-gene editing efficiency achieved 60%, representing a significant enhancement compared to editing in wild-type Bv916—particularly for dual-gene editing (Table 3). This finding provides a critical foundation for the broader application of this gene-editing platform.

### Exchange four native NRPS promoters for cyclic lipopeptides with strong constitutive promoters using CRISPR-Cas9 platforms

**Fig 3:**
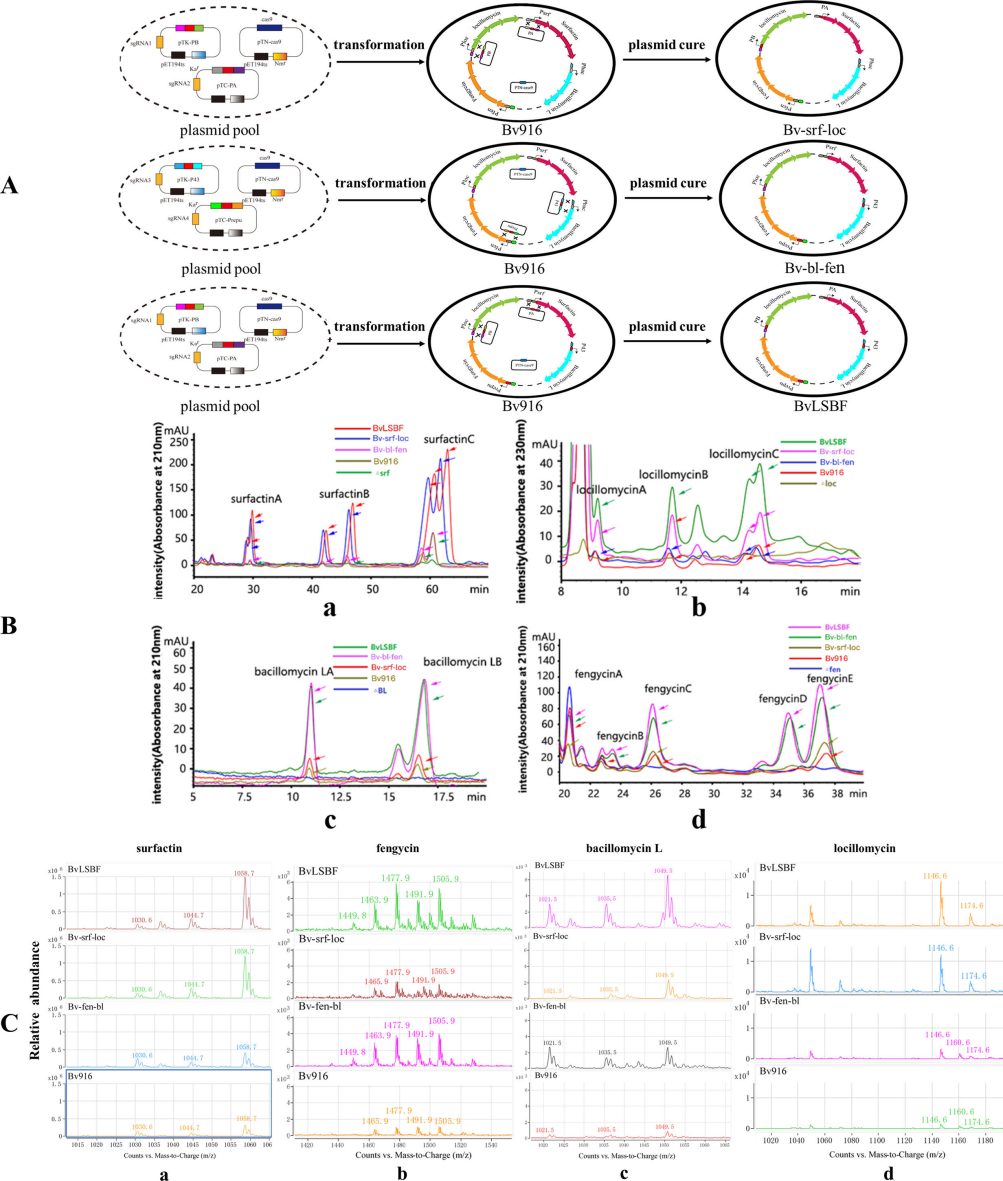
HPLC-MS analysis of four lipopeptides (surfactin, fengycin, bacillomycin L, locillomycin) produced by Bv916 and its genome-edited derivatives. (**A**) Construction of three Bv916 derivatives in which the native promoters of NRPSs for lipopeptides were replaced with strong constitutive promoters. The derivative Bv-srf-loc (Aa), in which the native promoters of surfactin and locillomycin were replaced by PA and PB promoters, respectively. The derivative Bv-bl-fen (Ab), in which the native promoters of bacillomycin L and fengycin were replaced with P43 and PrepU promoters, respectively. The derivative BvLSBF (Ac) in which the native promoters of surfactin, locillomycin, fengycin, and bacillomycin L were replaced by PA, PB, P43, and PrepU promoters, respectively. (**B**) HPLC spectrograms of four lipopeptides, including surfactin (Ba), locillomycin (Bb), bacillomycin L (Bc), and fengycin (Bd) produced by Bv916 and three derivatives. (**C**) Mass spectrometry analysis of the culture broth of Bv916 and its derivatives Bv-srf-loc, Bv-bl-fen, and BvLSBF.

To further validate the effectiveness of the three-plasmid CRISPR-Cas9 platform, we used it to replace the native promoters of four CLPS with strong promoters, thereby generating derivatives. Bv916 exhibits excellent antifungal, antibacterial, and antiviral properties as well as the ability to induce plant immunity, due to its ability to simultaneously secrete four cyclic lipopeptides, surfactin, bacillomycin L, fengycin, and locillomycin. The cyclic lipopeptides produced by *Bacillus* not only have excellent biocontrol activity against plant diseases, but also act as signaling molecules to regulate their own multicellular behavior. The endogenous promoters of their synthetic gene clusters are highly regulated in Bv916. Like other *Bacillus* strains, it typically produces about ten milligrams per liter of each type of cyclic lipopeptide or even less. Using the above three-plasmid gene editing system, after two rounds of editing, the endogenous promoters for the synthesis of four cyclic lipopeptides by Bv916 were successfully replaced with constitutive strong expression promoters. Using the three plasmids pTN-Cas9, pTC-PA-srf, and pTK-PB-loc cotransformed in Bv916, the derivative Bv-srf-loc was screened, and the endogenous promoters of surfactin and locillomycin were replaced by constitutive strong expression promoters PA and PB, respectively (Fig. 3Aa). By using the three plasmids pTN-Cas9, pTK-P43, and pTC-PrepU, the derivative Bv-bl-fen was obtained, in which the endogenous promoters of bacillomycin L and fengycin were replaced by the constitutive strong expression promoters P43 and PrepU, respectively (Fig. 3Ab). After eliminating the plasmids at a high temperature of 50°C, a second round of editing was performed by transforming pTN-Cas9, pTK-PB-loc, and pTC-PA-srf into Bv-bl-fen and obtaining the derivative BvLSBF (Fig. 3Ac). The endogenous promoters of all four cyclic lipopeptides in the derived BvLSBF genome were replaced by constitutive and highly expressed promoters. PCR and DNA sequencing analyses showed that the efficiency of using our three-plasmid gene editing platform to simultaneously edit two genes in each round was over 15%, which is further evidence that this platform can edit the genome of Bv916 and can be processed efficiently. Compared to other genetic engineering methods, genetically engineered strains constructed via this three-plasmid gene editing platform do not require the introduction of additional resistance marker genes and do not leave scars. Therefore, the derivatives Bv-srf-loc, Bv-bl-fen, and BvLSBF are environmentally friendly and ecologically harmless.

### Four families of cyclic lipopeptides produced by Bv916 and its derivatives

The concentrations of four cyclic lipopeptides of the family were monitored by HPLC-MS during the growth of domesticated Bv916 and its derivatives in stirred Erlenmeyer flasks for 48 h (Table 4 and Fig. 3B). After replacing the endogenous promoters of the four cyclic peptide gene clusters with constitutive promoters, the expression time in the derivatives was significantly prolonged compared to the parental strain, and the expression levels were also significantly increased. The yield of surfactin in the derivatives Bv-srf-loc and BvLSBF reached 780 mg/L and 710 mg/L, respectively, which is about a 6.5-fold and 5.9-fold increase compared to Bv916, respectively. The yield of locillomycin in the Bv-srf-loc and BvLSBF derivatives reached 29 and 75 mg/L, respectively, which is about a 2.6-fold and 6.8-fold increase compared to Bv916. The yield of bacillomycin L in the derivatives Bv-bl-fen and BvLSBF reached 570 and 680 mg/L, respectively, which is about a 9.2-fold and 10.9-fold increase compared to Bv916. The yield of fengycin in the derivatives Bv-bl-fen and BvLSBF reached 125 and 130 mg/L, respectively, which is about a 5.9-fold and 6.2-fold increase compared to Bv916, respectively. Mass spectrometry analysis further demonstrates that the strategy of replacing the endogenous promoters of the four cyclic lipopeptides with constitutively high-expression promoters in Bv916 can significantly increase the expression levels of all four cyclic lipopeptides (Fig. 3C).

**TABLE 4:** Production of four families of CLPs—surfactins, bacillomycin Ls, locillomycins, and fengycins—by Bv916 and its derivatives grown in LB medium

CLPs	CLP production (mg L^−1^)							
	Bv916		Bv-srf-loc		Bv-bl-fen		BvLSBF	
	12 h	48 h	12 h	48 h	12 h	48 h	12 h	48 h
Surfactins	30 ± 9	120 ± 35	310 ± 50	780 ± 120	31 ± 9	129 ± 35	300 ± 61	710 ± 140
Bacillomycin Ls	0	62 ± 23	0	75 ± 5	150 ± 20	570 ± 100	180 ± 30	680 ± 140
Locillomycins	0	11 ± 2	18 ± 5	29 ± 9	0	13 ± 4	16 ± 5	75 ± 21
Fengycins	0	21 ± 5	0	27 ± 7	32 ± 8	125 ± 25	24 ± 7	130 ± 25

### Antimicrobial activities of Bv916 and its derivatives

**Fig 4:**
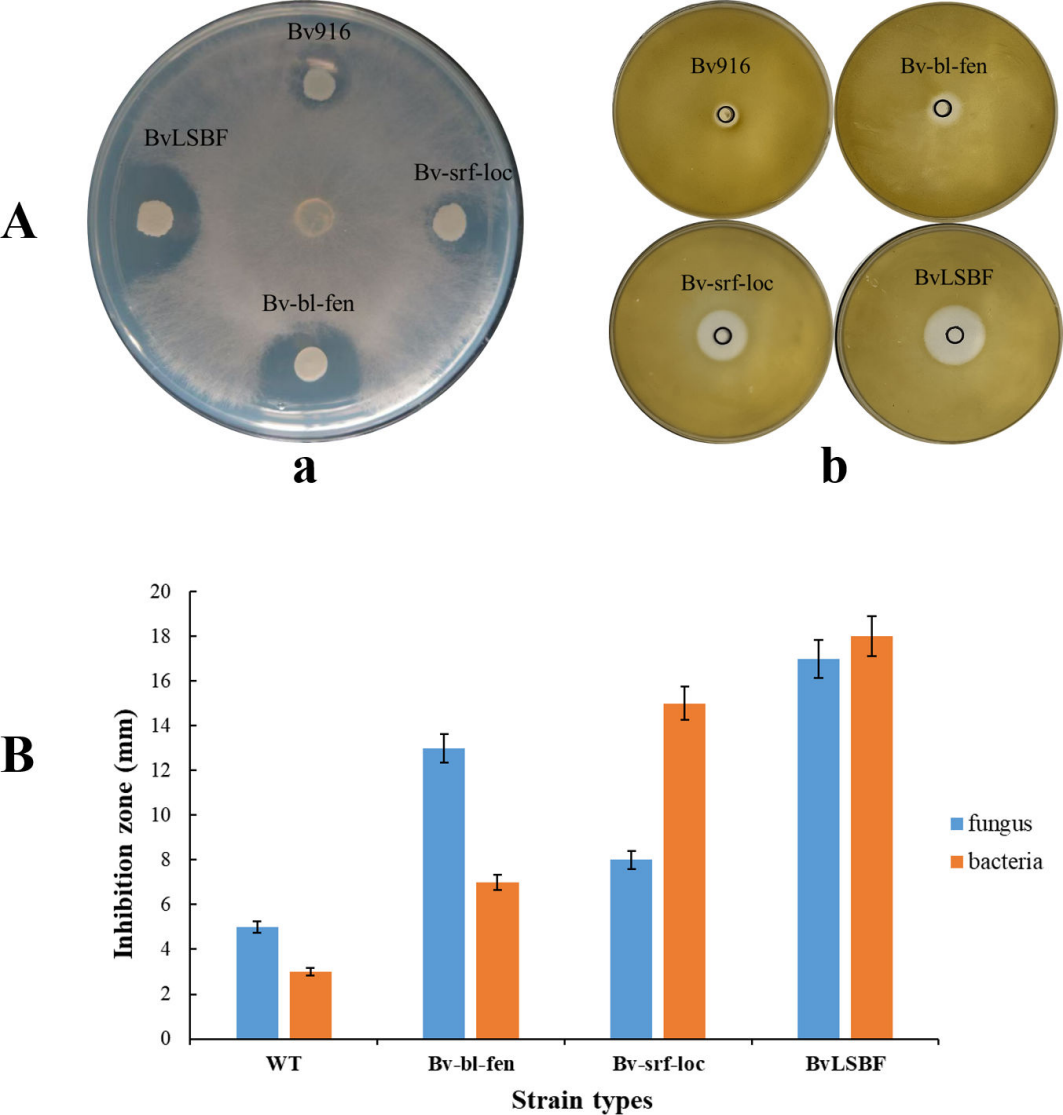
Study on the antibacterial and antifungal activity of Bv916 and its derivatives. (Aa) Antifungal activity of WT Bv916 and its derivatives (Bv-srf-loc, Bv-bl-fen, and BvLSBF) against *R. solani*. (Ab) Antibacterial activity of WT Bv916 and its derivatives (Bv-srf-loc, Bv-bl-fen, and BvLSBF) against *S. aureus*. (**B**) Measurement of the inhibition zones of WT Bv916, Bv-srf-loc, Bv-bl-fen, and BvLSBF against fungi and bacteria. Each treatment was performed in triplicate.

To further visually evaluate the antimicrobial capacity of the high-yield CLP derivatives, the antifungal and antibacterial properties of these derivatives were tested (Table 5). We concluded that the antagonistic properties of Bv916 and its derivatives are closely related to the type and production level of cyclic lipopeptides they secrete. The simultaneous overproduction of surfactin and locillomycin in the derivative Bv-srf-loc significantly increased its antibacterial activity without changing its antifungal properties. In contrast, simultaneous overproduction of bacillomycin L and fengycin in the derivative Bv-bl-fen significantly increased its antifungal activity without altering its antibacterial properties. As expected, simultaneous overproduction of all four CLPs in the derivative BvLSBF significantly increased both its antibacterial and antifungal activities (Fig. 4). Therefore, the BvLSBF derivative has significant potential for development as an effective biocontrol agent for comprehensive biological control of various fungal and bacterial diseases in plants.

**TABLE 5:** Growth inhibition activities of the Bv916 and its derivatives^*a*^

Strain	Antagonistic activity			
	Bv916	Bv-srf-loc	Bv-bl-fen	BvLSBF
*S. aureus*	±	++	±	+++
*E. coli*	±	++	±	+++
*Ralstonia solanacearum*	±	++	±	+++
*F. oxysporum*	±	±	+	+++
*F. petridiphilum*	±	±	+	+++
*R. solani*	±	±	++	+++

^*a*^ The intensity of antagonistic activity was assessed based on the size of the growth inhibition zones from the wells in which supernatant samples were deposited to the edge of the spreading fungal mycelium or cell colony. ±, 1 to 7 mm; +, 8 to 12 mm; + +, 13 to 16 mm; + + +, 17 mm or more.

### Antibiotic susceptibility test

To further evaluate the safety of the derivatives (Bv-srf-loc, Bv-bl-fen, and BvLSBF), antibiotic susceptibility testing was performed (Table 6). The results showed that no significant changes in antibiotic susceptibility were observed in the derivatives compared to the wild-type Bv916, indicating that they retained high sensitivity to all tested antibiotics (Ampicillin, Kanamycin, Erythromycin, and Chloramphenicol). This suggests that the derivatives retain the food-grade safety characteristics of the wild-type Bv916.

**TABLE 6:** Susceptibility of Bv916 and its derivatives to antibiotics^*a*^

Antibiotics	µg/disc	Strains			
		Bv916	Bv-srf-loc	Bv-bl-fen	BvLSBF
Ampicillin	10	S	S	S	S
Kanamycin	10	S	S	S	S
Erythromycin	15	S	S	S	S
Chloramphenicol	30	S	S	S	S

^*a*^ S, susceptible.

### Colonization ability and biocontrol efficacy of Bv916 and its derivatives

**Fig 5:**
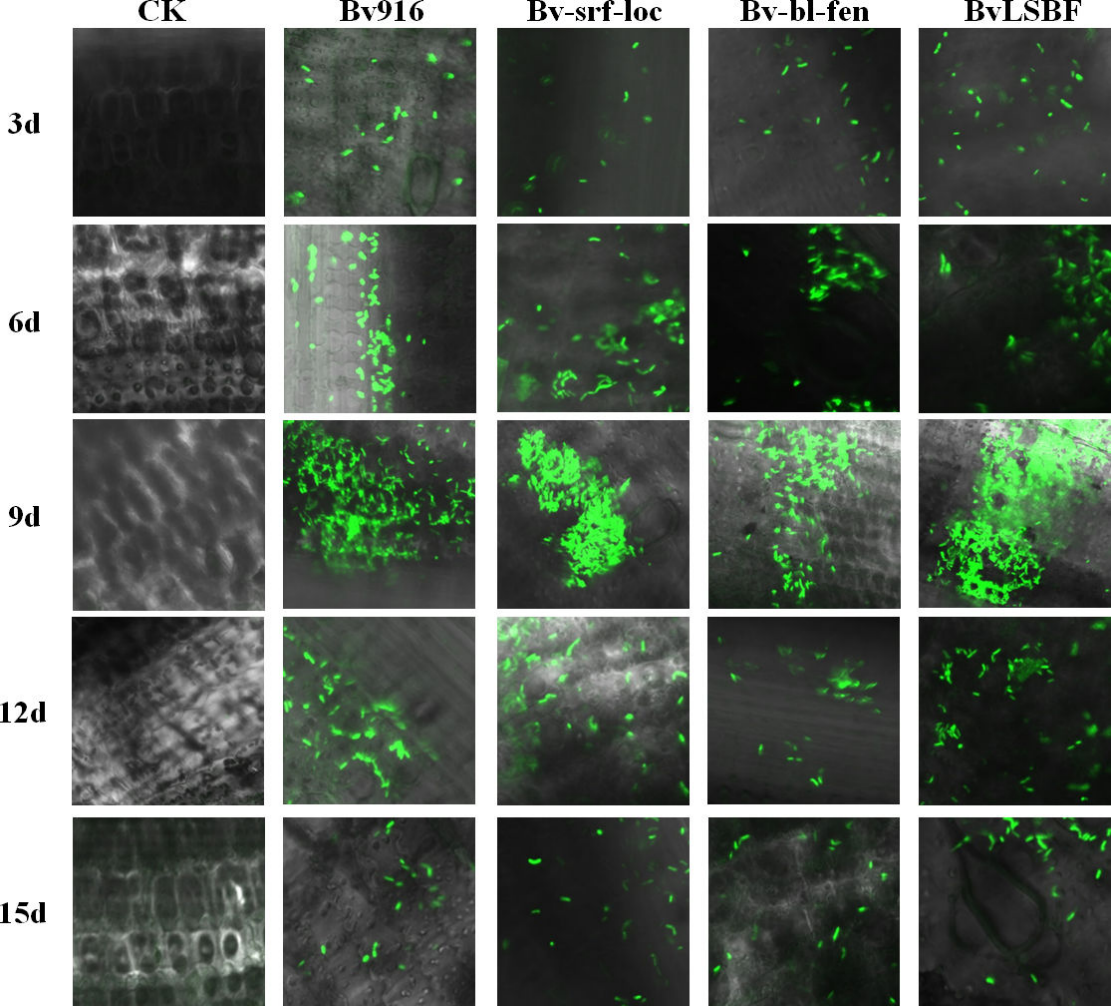
Colonization of the rice plant against rice sheath blight. The colonization patterns of WT Bv916 and its derivatives (Bv-srf-loc, Bv-bl-fen, and BvLSBF) were essentially identical. Over time, the bacterial population initially increased, peaked at approximately day 9, and subsequently declined until nearly disappearing by day 15.

To further evaluate the environmental adaptability of Bv916 and its derivatives and their potential application in plant disease control. GFP labeling technology was employed to evaluate the colonization capacity of Bv916 and its derivatives in rice stems infected with *R. solani*. Microscopic observations revealed consistent fluorescence patterns among the four bacterial strains. At day 9, cells exhibited intense green fluorescence with notable aggregation effects. Following this, the total bacterial population began to decline, and only residual bacterial cells remained for all four strains by day 15. Compared with Bv916, the three derivatives showed no significant changes in colonization capacity (Fig. 5).

**Fig 6:**
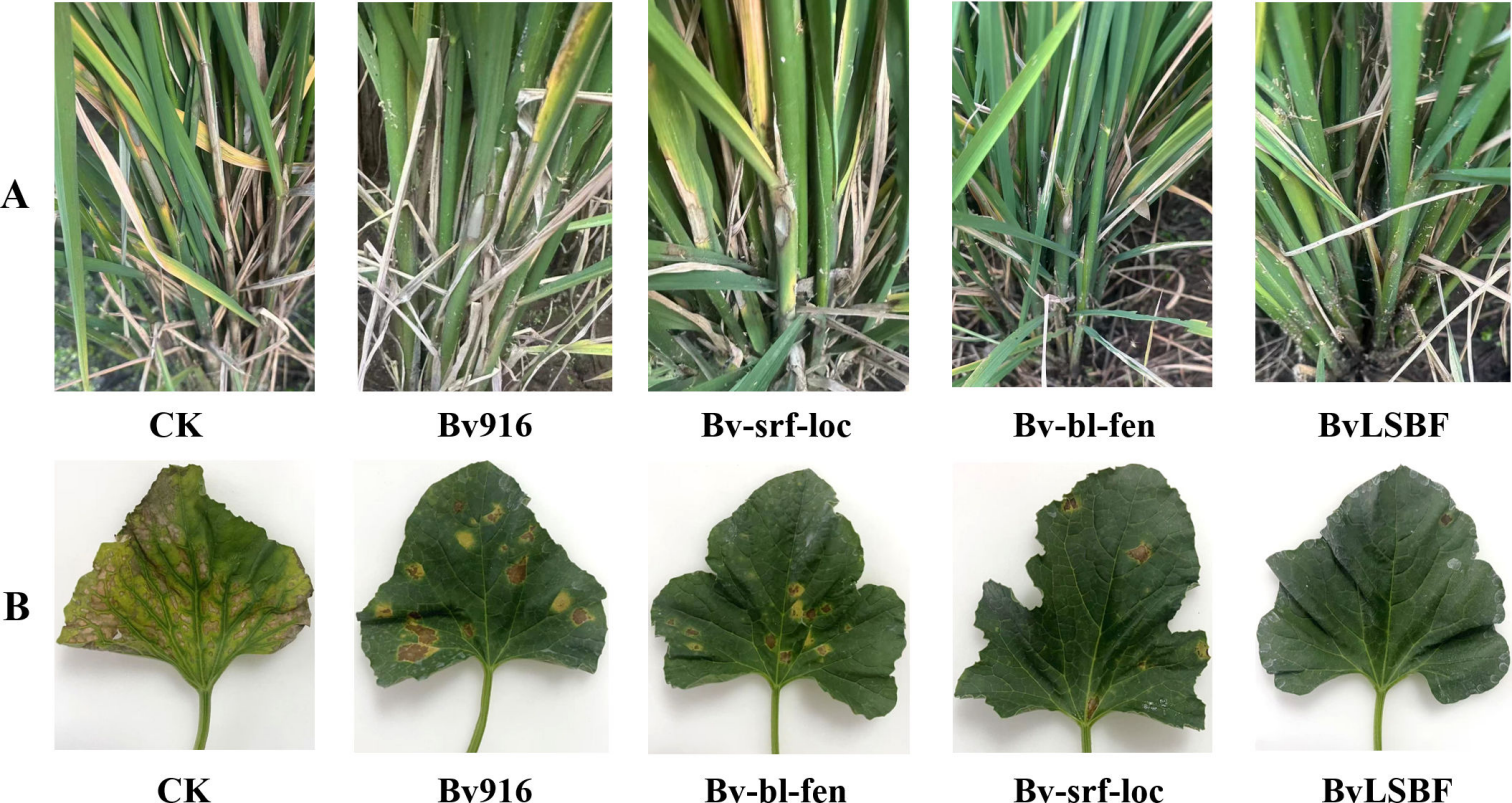
Biocontrol assays of wild-type Bv916 and its derivatives against rice sheath blight in a potted system and against angular leaf spot of muskmelon in a field system. (**A**) Changes in the lesion length of rice sheath blight under treatment with WT Bv916 and its derivatives (Bv-srf-loc, Bv-bl-fen, and BvLSBF), CK served as the control group, treated with distilled water only. (**B**) Changes in the severity of angular leaf spot under treatment with WT Bv916 and its derivatives (Bv-srf-loc, Bv-bl-fen, and BvLSBF), CK served as the control group, treated with water only.

The biocontrol efficacy of wild-type Bv916 and its derivatives (Bv-srf-loc, Bv-bl-fen, and BvLSBF) was assessed against rice sheath blight and angular leaf spot, with the results presented in Table 7. All four strains exhibited varying degrees of disease control compared to the control group. For rice sheath blight, treatment significantly reduced the lesion length compared to the control group (Fig. 6A). Among the tested strains, the derivatives Bv-bl-fen and BvLSBF displayed the most notable biocontrol effects against *R. solani*, with efficacies of 85% and 90%, respectively. For bacterial angular leaf spot of muskmelon, compared to the control group (Fig. 6B), strain Bv-srf-loc and BvLSBF fermentation broth demonstrated significant biocontrol efficacy against *Pseudomonas syringae pv. lachrymans*, reaching 87% and 91%, respectively.

**TABLE 7:** Biocontrol of rice sheath blight and angular leaf spot of Bv916 and its derivatives

Disease types	Biocontrol efficiency %			
	Bv916	Bv-bl-fen	Bv-srf-loc	BvLSBF
Rice sheath blight	50 ± 2.50	85 ± 4.25	62 ± 3.10	90 ± 4.50
Angular leaf spot	43 ± 2.15	57 ± 2.85	87 ± 4.35	91 ± 4.55

## DISCUSSION

In recent years, various powerful toolkits based on the CRISPR-Cas9 system have been developed for highly efficient genome editing in *B. subtilis* (26). Zhang et al. developed an all-in-one plasmid CRISPR-Cas9 system, which successfully knocked out five genes with an efficiency of 35% to 55%; however, this knockout efficiency remained relatively low (37). In contrast, the double plasmid CRISPR-Cas9 system exhibits higher assembly and editing efficiency. For instance, the double plasmid CRISPR-Cas9 system constructed by So et al. achieved a single-gene knockout efficiency of approximately 100%, a point mutation efficiency of ~68%, and a large-fragment genomic deletion efficiency of ~80%, along with a single-gene insertion efficiency of ~97% (46). Nevertheless, this system is incapable of simultaneously editing two genes. Moreover, in previous reports, most wild-type Bacillus strains required the support of the lambda red recombination system or chromosomal modulating peptides as well as the controlled expression of Cas9 protein and sgRNA to improve the efficiency and accuracy of gene editing (26, 37, 40, 47). To address these limitations, this study developed a three-plasmid CRISPR-Cas9 system suitable for Bv916, which demonstrates high editing efficiency, enables simultaneous editing of two genes, and features user-friendly operation (Fig. 1). The gene-editing platform features a host-specific thermosensitive replication origin, and the antibiotic resistance marker-carrying plasmid can be eliminated at 50°C, maintaining the food-grade safety characteristics of the strain. The efficiency of editing a single gene using this gene editing platform is more than 80%, while the efficiency of editing two genes at the same time is about 20%. It took approximately 5 working days to complete one round of gene editing. In the absence of the CRISPR system, the recombination efficiency of the expression vector in Bv916 was very low, only 5% (Table S1).

However, the gene editing efficiency of this platform still needs to be improved, particularly for simultaneous dual-gene editing. Studies have revealed that the pheromone ComX is essential for competence development in bacteria. When extracellular ComX reaches a critical concentration, it activates the ComPA system, which upregulates ComS. This subsequently inhibits ComK degradation (through competitive binding of ComS with ComK to proteases). As the master transcriptional regulator of genetic competence, ComK at high concentrations induces expression of ComEA, a DNA-binding protein strictly required for transformation. RecA functions as a key enzyme that facilitates sequence matching between two DNA molecules, enabling base pairing between homologous regions of these molecules (48, 49). The native promoters of ComX and RecA in Bv916 were replaced with the strong promoters P43 and PrepU, respectively (Fig. 2). The introduction of these strong promoters enabled high-level expression of ComX and RecA, thereby improving the editing efficiency of this platform. The platform achieved 96% efficiency for single-gene editing and 61% efficiency for simultaneous dual-gene editing. Even in the absence of the CRISPR system, high expression of ComX still enhances the transformation efficiency of the expression vector in Bv916, while high expression of RecA further improves its recombination efficiency (Table S1). These results further confirm that ComX is associated with bacterial transformation efficiency, while RecA is involved in bacterial recombination efficiency (48, 49). This lays a more solid foundation for the platform’s applications, but further studies are still needed to address the potential drawbacks and solutions associated with the sustained expression of derivatives.

The CLPs secreted by *Bacillus* are a type of hybrid secondary metabolite composed of hydrophilic oligopeptide heads and hydrophobic fatty acid tails via lactone or lactam bonds. These CLPs not only have antibacterial and antiviral effects and activate the plant immune system, but also act as signaling molecules that intervene in the multicellular behavior of themselves or pathogenic microorganisms, making them the primary biocontrol factor of *Bacillus* (10, 12, 50, 51). Compared to *B. subtilis*, the genome of Bv more frequently contains large NRPS and PKS gene clusters with a stronger ability to secrete and synthesize large cyclic peptides and polyketide compounds, resulting in better biological control effects on plant diseases (13, 14, 19, 52). In addition to *B. velezensis* SQR9, Bv916 is also a strain of an effective biocontrol agent. The commercialized Bv916 has been successfully used in the biological control of rice blight and rice blast, and further genetic modifications have important economic value in making it a more efficient biocontrol agent (9). To further enhance the biocontrol efficacy of Bv916 and validate the effectiveness of the aforementioned platform system, the native promoters of four CLPs were replaced with strong promoters using the established gene-editing platform. HPLC-MS (Fig. 3) and inhibition zone assays (Fig. 4) demonstrated a significant increase in their production levels. The results demonstrated that Bv-srf-loc exhibited significant antibacterial activity, while Bv-bl-fen showed stronger antifungal effects. Notably, BvLSBF, which simultaneously overexpresses all four CLPS, displayed markedly enhanced activity against both bacteria and fungi (Fig. 6). This finding aligns with previous reports indicating that Surfactins and Locillomycins primarily exhibit antibacterial properties, whereas Bacillomycin L and Fengycins display pronounced antifungal activity (9, 14). Notably, the engineered strain constructed using the three-plasmid gene editing platform also exhibits excellent safety, as the introduced gene fragments show no genetic drift and retain antibiotic sensitivity consistent with the wild-type strain (Table 6), preserving its food-safe characteristics. Additionally, its derivatives demonstrate strong environmental adaptability and robust colonization capacity (Fig. 5). Based on this platform, we also achieved the display of African swine fever virus key antigens on the *Bacillus* spore surface at the same time (53).

In summary, this study successfully constructed a three-plasmid gene editing platform based on the CRISPR-Cas9 system and used this gene editing method to achieve gene insertion, gene replacement, and gene deletion in Bv916. The obtained derivative BvLSBF through this gene editing platform, with the improvement of the production of four CLPs, maintains the environmental friendliness status, and there is the potential for wide applications in the biological control of plant diseases. It will be interesting to further optimize this three-plasmid-mediated iterative genome engineering platform and modify more loci simultaneously and make them more precise in Bv916. Modification of Bv916 by this food-grade gene editing platform may lead to advances in genome reduction, cell factory, bioremediation, industrial fermentation, and sustainable agriculture. Although this gene-editing system demonstrates potential for application in the wild-type Bv916 strain, certain limitations remain to be further improved. Firstly, the wild-type Bv916 strain exhibits low competence. Compared to the model strain *B. subtilis* 168, Bv916 exhibits relatively low editing efficiency. Secondly, although electroporation is more convenient than chemical transformation, an effective electroporation method for Bv916 has not yet been established, leaving chemical transformation as the only currently viable option. More critically, the current system relies on plasmids to deliver the sgRNA, Cas9 protein, and homologous recombination templates. Future work should focus on integrating Cas9 into the Bv916 genome and utilizing linearized sgRNA and homologous recombination fragments, thereby eliminating plasmid dependency and further optimizing the gene-editing system for wild-type strains.

## SUPPLEMENTAL MATERIAL

Supplemental material aem.01389-25-s0001.docx Table S1; Fig. S1 to S18.

ASM does not own the copyrights to Supplemental Material that may be linked to, or accessed through, an article. The authors have granted ASM a non-exclusive, world-wide license to publish the Supplemental Material files. Please contact the corresponding author directly for reuse.
