# Supplementary material for: A three-plasmid-containing CRISPR-Cas9 platform to engineer Bacillus velezensis 916 as an efficient biocontrol agent
Source: Appl Environ Microbiol. 2025 Sep 2;91(10):e01389-25. doi: 10.1128/aem.01389-25 (PMC12542745; doi:10.1128/aem.01389-25)
Supplement: Supplemental material — Table S1; Fig. S1 to S18. [file aem.01389-25-s0001.docx]

**Supporting Information**

**Supplementary Tables**

**Table S1.** **Efficiency of the genome editing of Bv916 and its derivatives (△ComX,△RecA, △ComX△RecA) without relying on the CRISPR-Cas9 system**

**Supplementary Figures**

**Fig S1. Schematic diagram of the construction process for plasmid pTN-Cas9**

**Fig S2. Schematic diagram of the construction process for plasmid pTK-CotB-GFP**

**Fig S3. Schematic diagram of the construction process for plasmid pTC-CgeA-RFP**

**Fig S4. Schematic diagram of the construction process for plasmid pTC-PA-srf**

**Fig S5. Schematic diagram of the construction process for plasmid pTK-PB-loc**

**Fig S6. Schematic diagram of the construction process for plasmid pTK-P43-bl**

**Fig S7. Schematic diagram of the construction process for plasmid pTC-PrepU-fen**

**Fig S8. Schematic diagram of the construction process for plasmid pTK-comX**

**Fig S9. Schematic diagram of the construction process for plasmid pTC-recA**

**Fig S10.** **The sequence of pTN-Cas9 with a nucleotide length of 10513 bp**

**Fig S11.** **The sequence of pTK-CotB-GFP with a nucleotide length of 8080 bp**

**Fig S12.** **The sequence of pTC-CgeA-RFP with a nucleotide length of 8307 bp**

**Fig. S13.** **The sequence of pTC-PA-srf with a nucleotide length of 7584 bp**

**Fig S14.** **The sequence of pTK-PB-loc with a nucleotide length of 7442 bp**

**Fig S15.** **The sequence of pTK-P43-bl with a nucleotide length of 7347 bp**

**Fig S16** **The sequence of pTC-PrepU-fen with a nucleotide length of 7742 bp**

**Fig S17** **The sequence of pTK-comX with a nucleotide length of 6954 bp**

**Fig S18** **The sequence of pTC-recA with a nucleotide length of 7285 bp**

**Supplementary Tables**

**Table S1. Efficiency of the genome editing of Bv916 and its derivatives (△ComX**, **△RecA**, **△ComX**△**RecA**) **without relying on the CRISPR-Cas9 system**

| **recipient strains** | **Gene edtiing Types** | **Transformants** | **Transformation efficiency** | **Recombination efficiency** | | **Total editing efficiency** |
| --- | --- | --- | --- | --- | --- | --- |
| **Bv916** | pSG1154 | 112±5 | 0.11% | 5.36% | 5.90×10^-5^ | |
|  | pSG1170 | 80±4 | 0.08% | 5.00% | 4.00×10^-5^ | |
|  | pSG1164 | 63±3 | 0.06% | 4.76% | 2.86×10^-5^ | |
| **△ComX** | pSG1154 | 449±22 | 0.45% | 5.12% | 2.30×10^-4^ | |
|  | pSG1170 | 323±15 | 0.30% | 5.26% | 1.58×10^-4^ | |
|  | pSG1164 | 251±12 | 0.25% | 5.18% | 1.30×10^-4^ | |
| **△RecA** | pSG1154 | 114±5 | 0.11% | 10.53% | 1.16×10^-4^ | |
|  | pSG1170 | 85±5 | 0.09% | 10.59% | 9.53×10^-5^ | |
|  | pSG1164 | 67±4 | 0.07% | 10.45% | 7.32×10^-5^ | |
| **△ComX**△**RecA** | pSG1154 | 454±24 | 0.45% | 10.13% | 4.56×10^-4^ | |
|  | pSG1170 | 327±16 | 0.33% | 10.39% | 3.43×10^-4^ | |
|  | pSG1164 | 258±13 | 0.26% | 10.47% | 2.72×10^-4^ | |

^a^ pSG1154 (7.6Kb, BGSC No.: ECE153), pSG1170 (6.7Kb, BGSC No.: ECE156) , pSG1164 (4.8Kb, BGSC No.: ECE155): expression vector and *gfp*-fusion ectopic integretion vector;

^b.^ Transformation efficiency (%) = **Number of** transformants / **Number of competent cells. The competent Bv916 typically yields approximately 10^5^ CFU.**;

^c.^ Pick single colonies of transformants for another round of fermentation, then perform dilution plating and record the number of luminescent colonies. Recombination efficiency (%) = Number of luminescent colony (after Dilution Plating) / Number of transformants;

^d.^ Total editing efficiency = Conversion efficiency × Recombination efficiency

**Supplementary Figures**


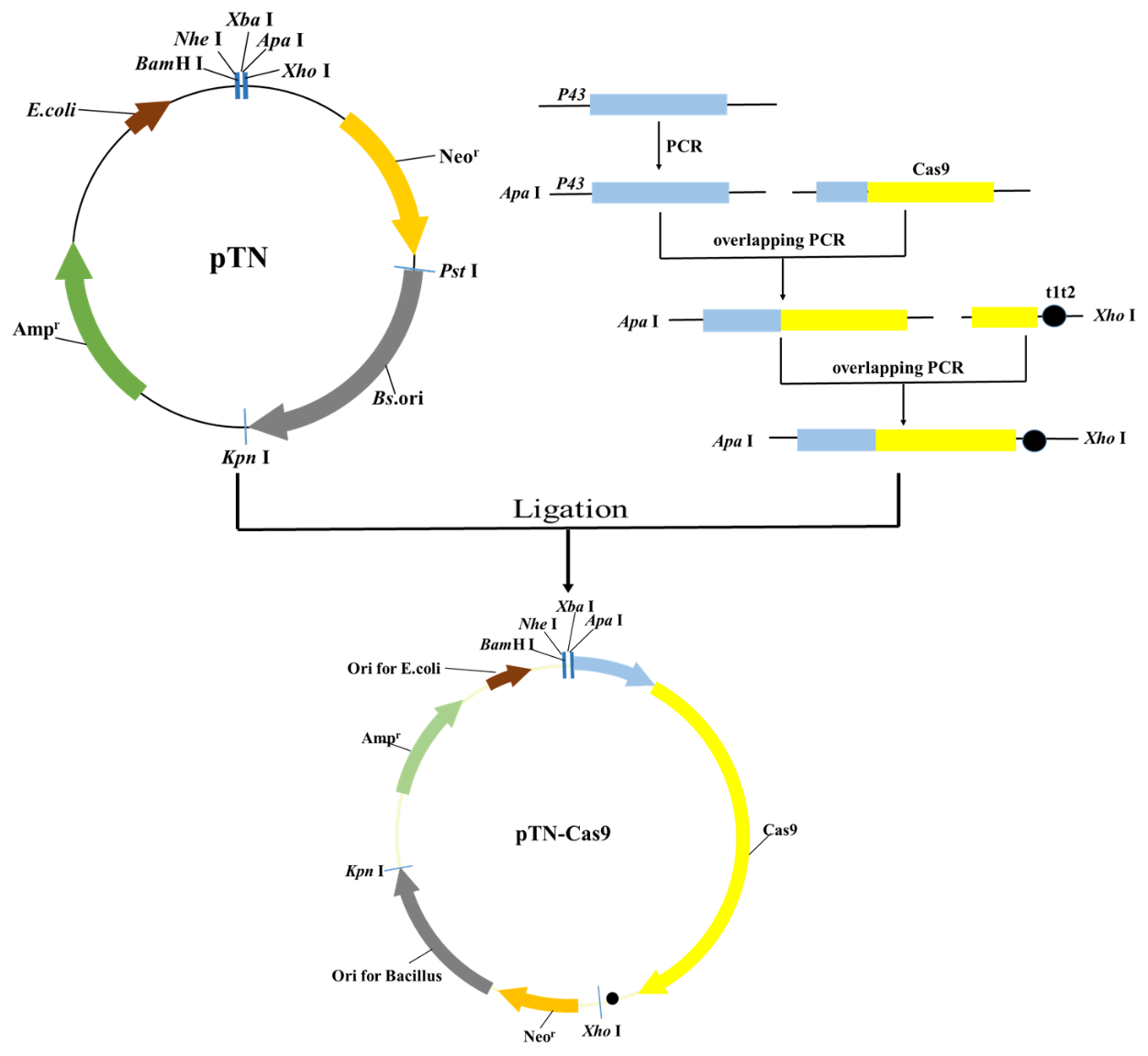


**Fig. S1 Schematic diagram of the construction process for plasmid pTN-Cas9**


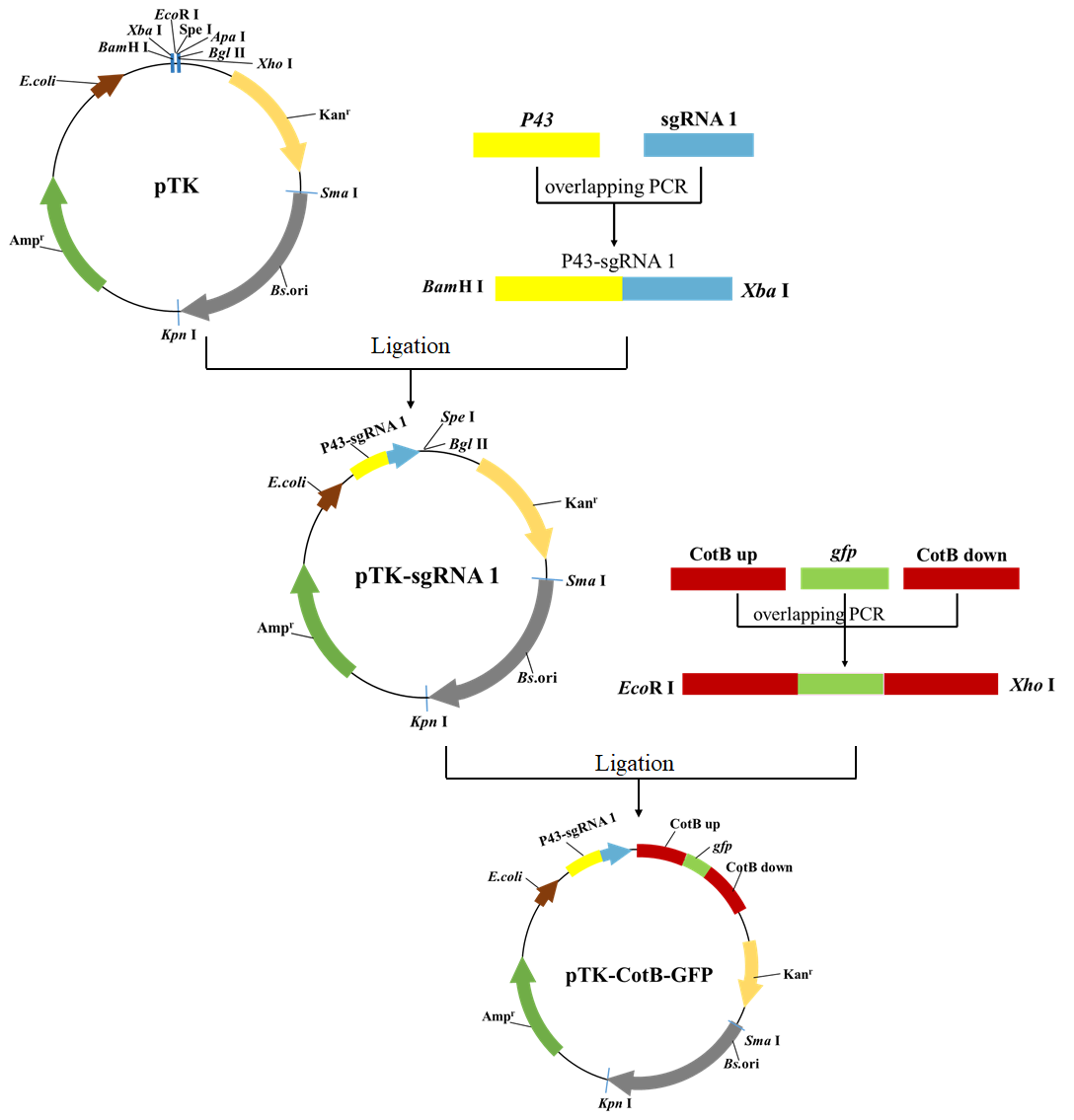


**Fig. S2 Schematic diagram of the construction process for plasmid pTK-CotB-GFP**


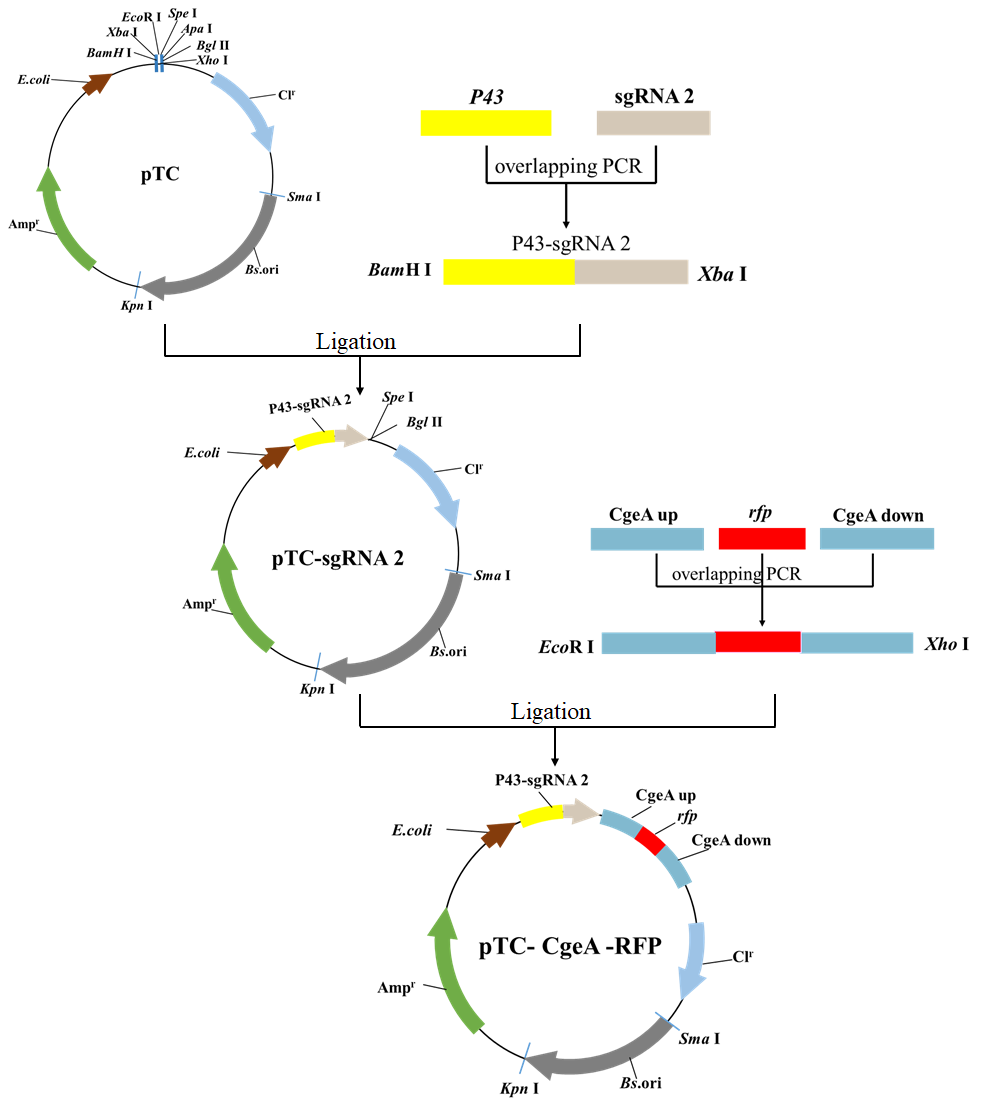


**Fig. S3 Schematic diagram of the construction process for plasmid pTC-CgeA-RFP**


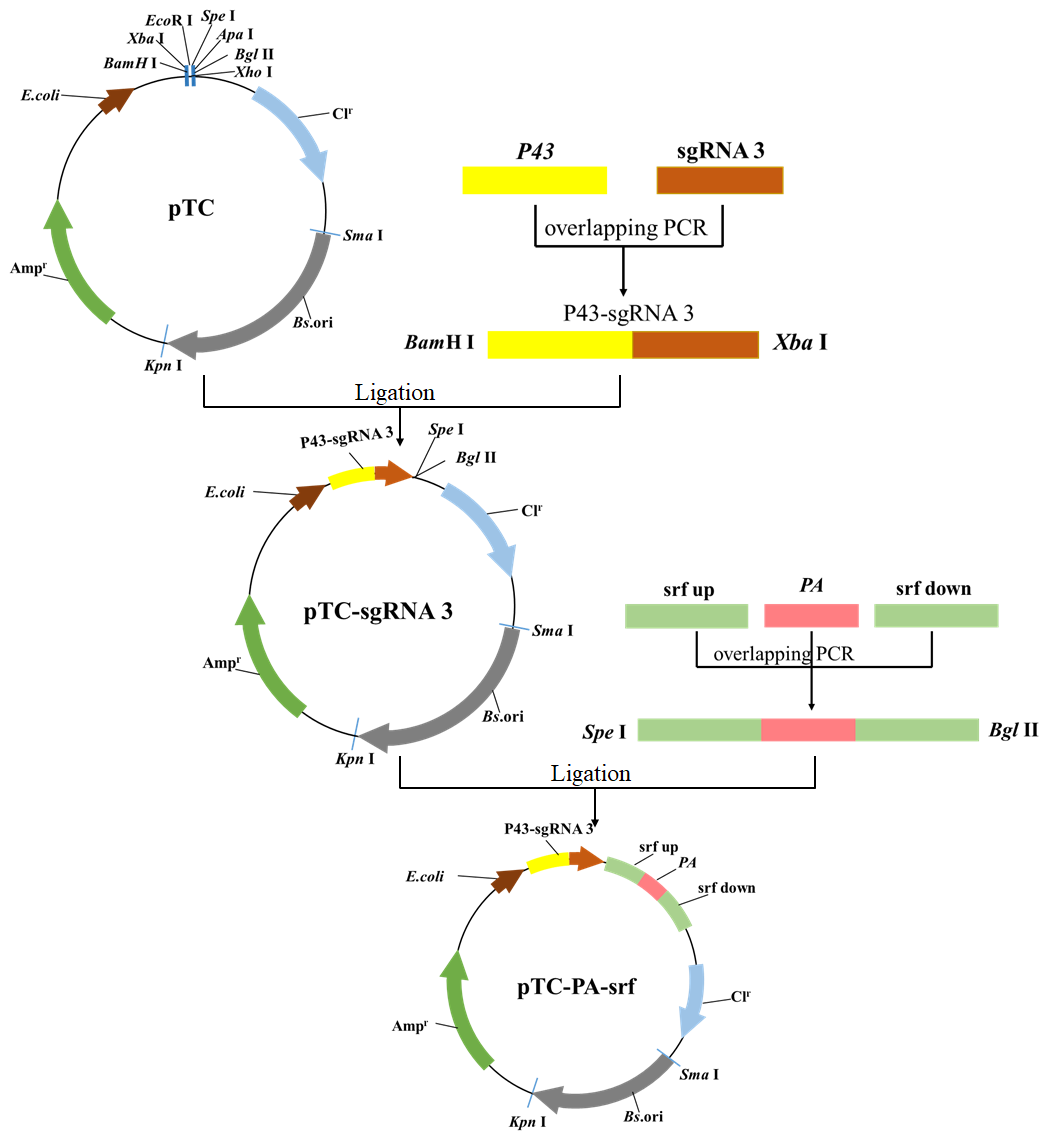


**Fig. S4 Schematic diagram of the construction process for plasmid pTC-PA-srf**


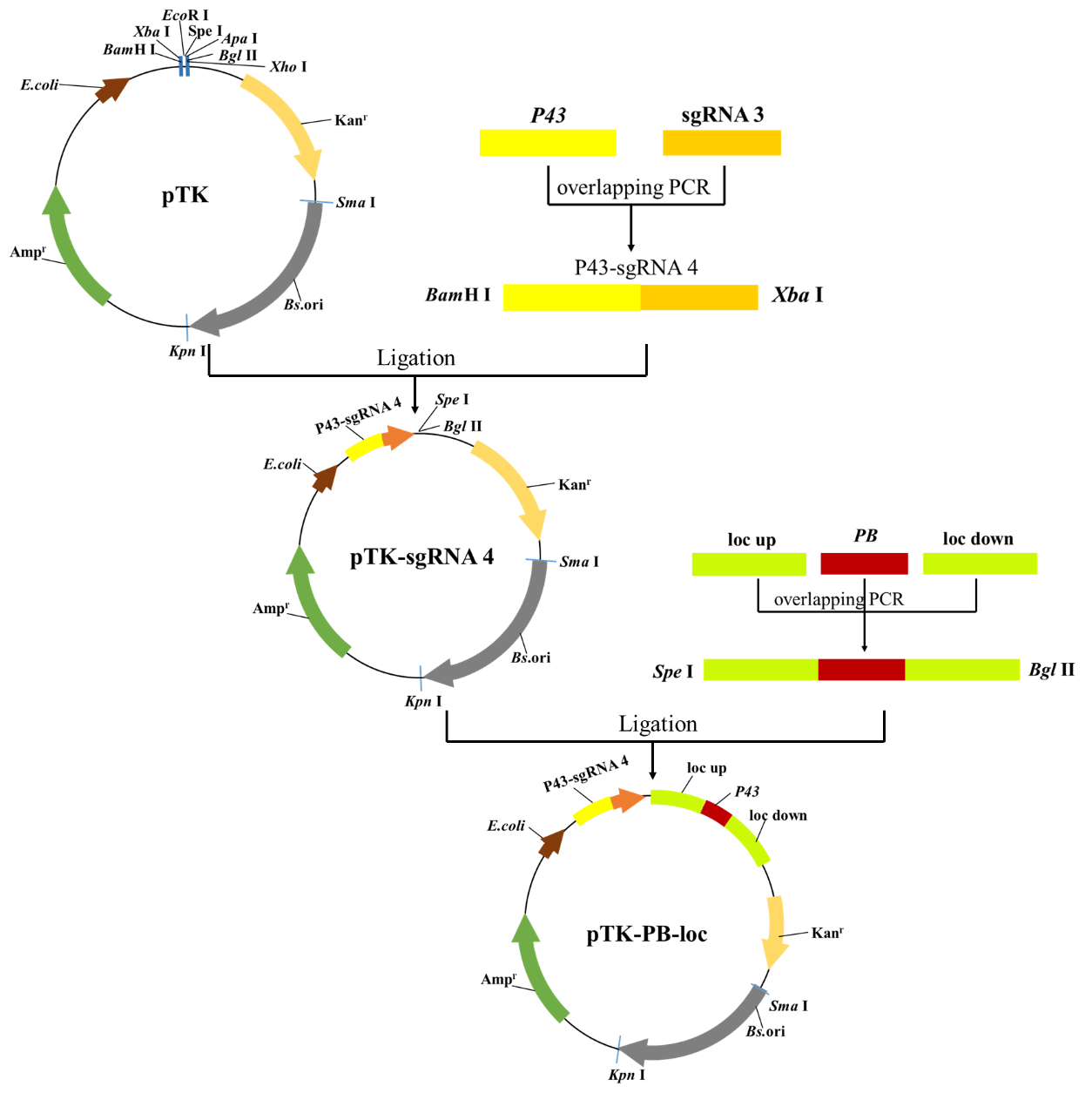


**Fig. S5 Schematic diagram of the construction process for plasmid pTK-PB-loc**


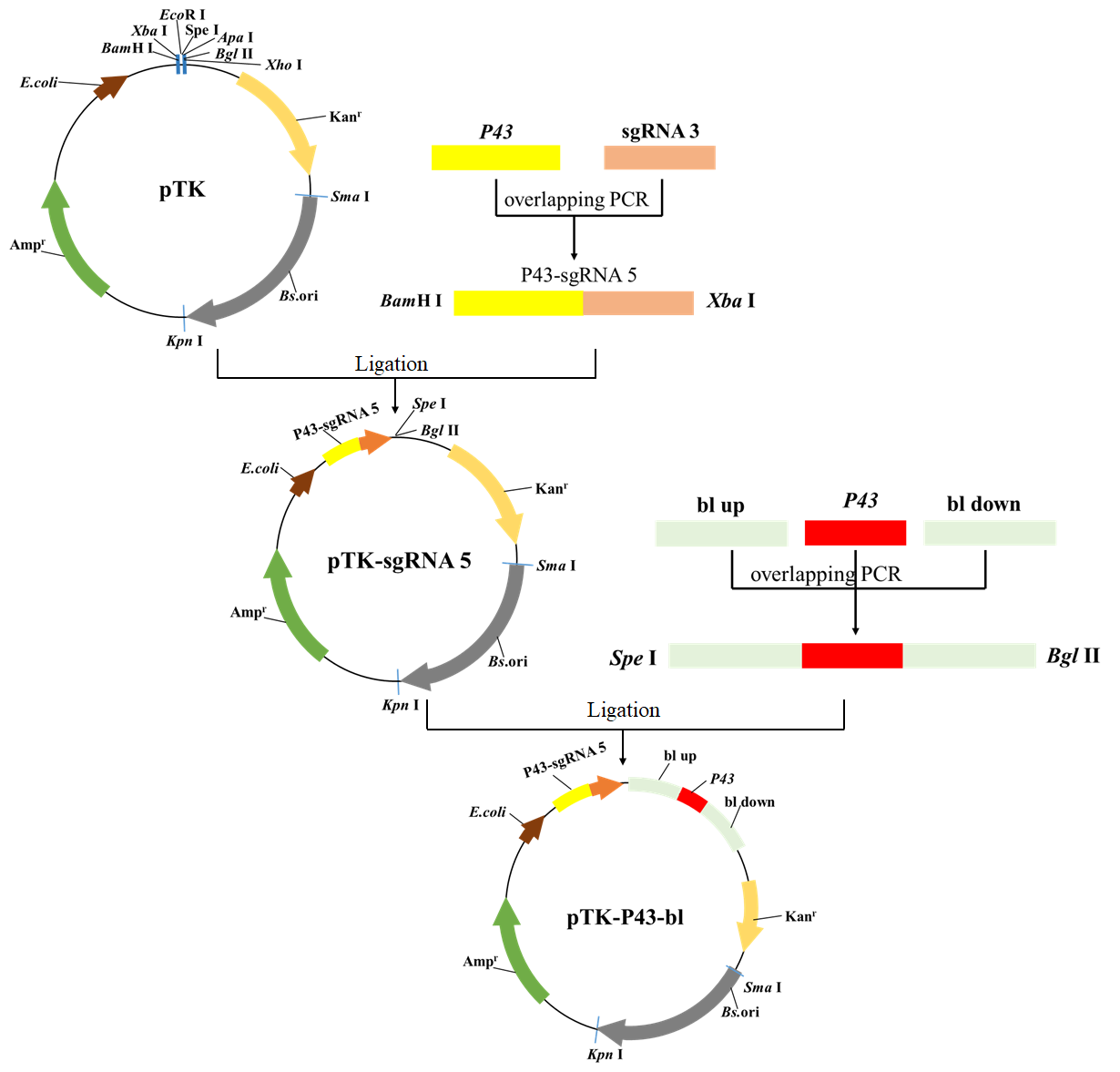


**Fig. S6 Schematic diagram of the construction process for plasmid pTK-P43-bl**


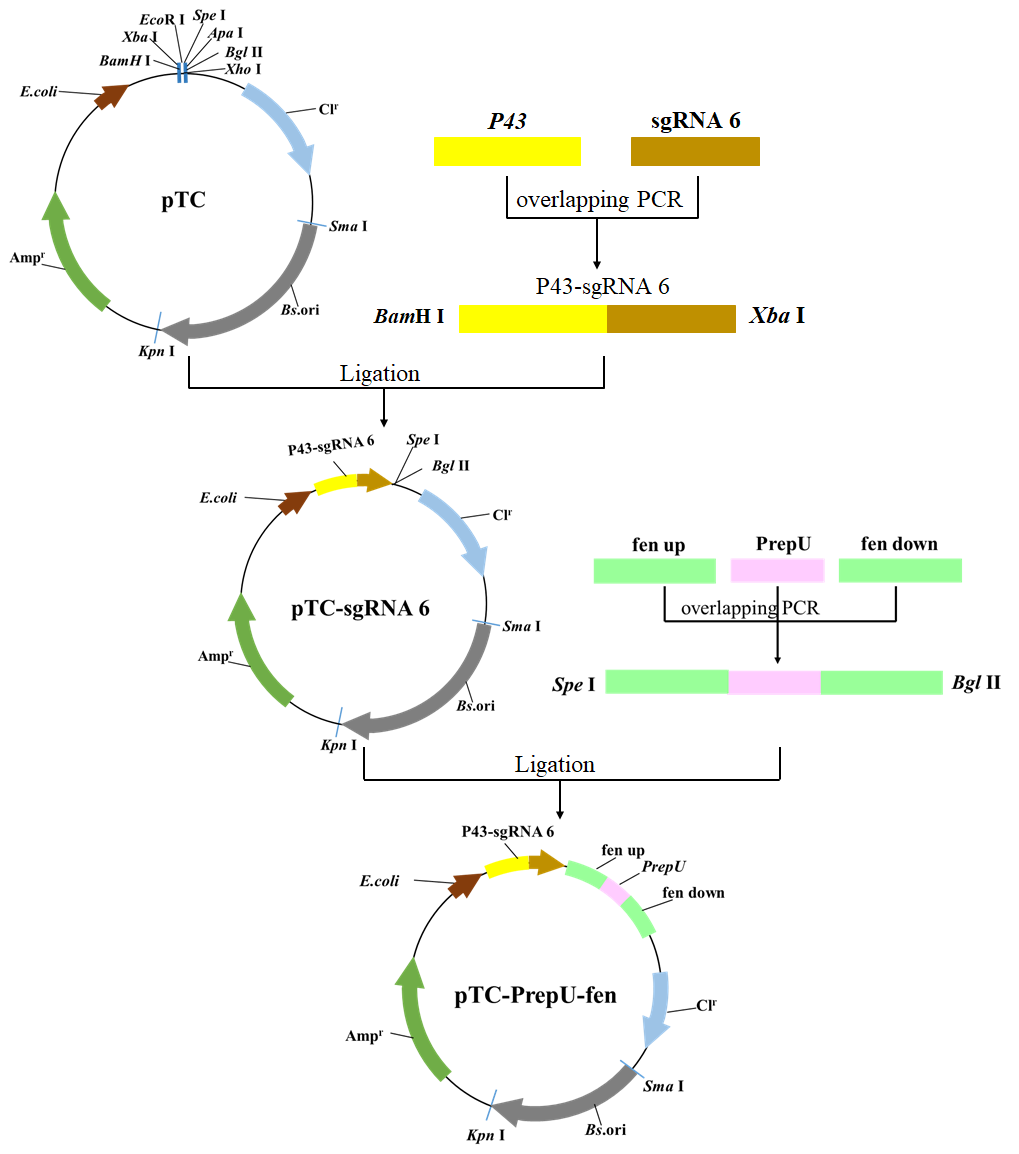


**Fig. S7 Schematic diagram of the construction process for plasmid pTC-PrepU-fen**


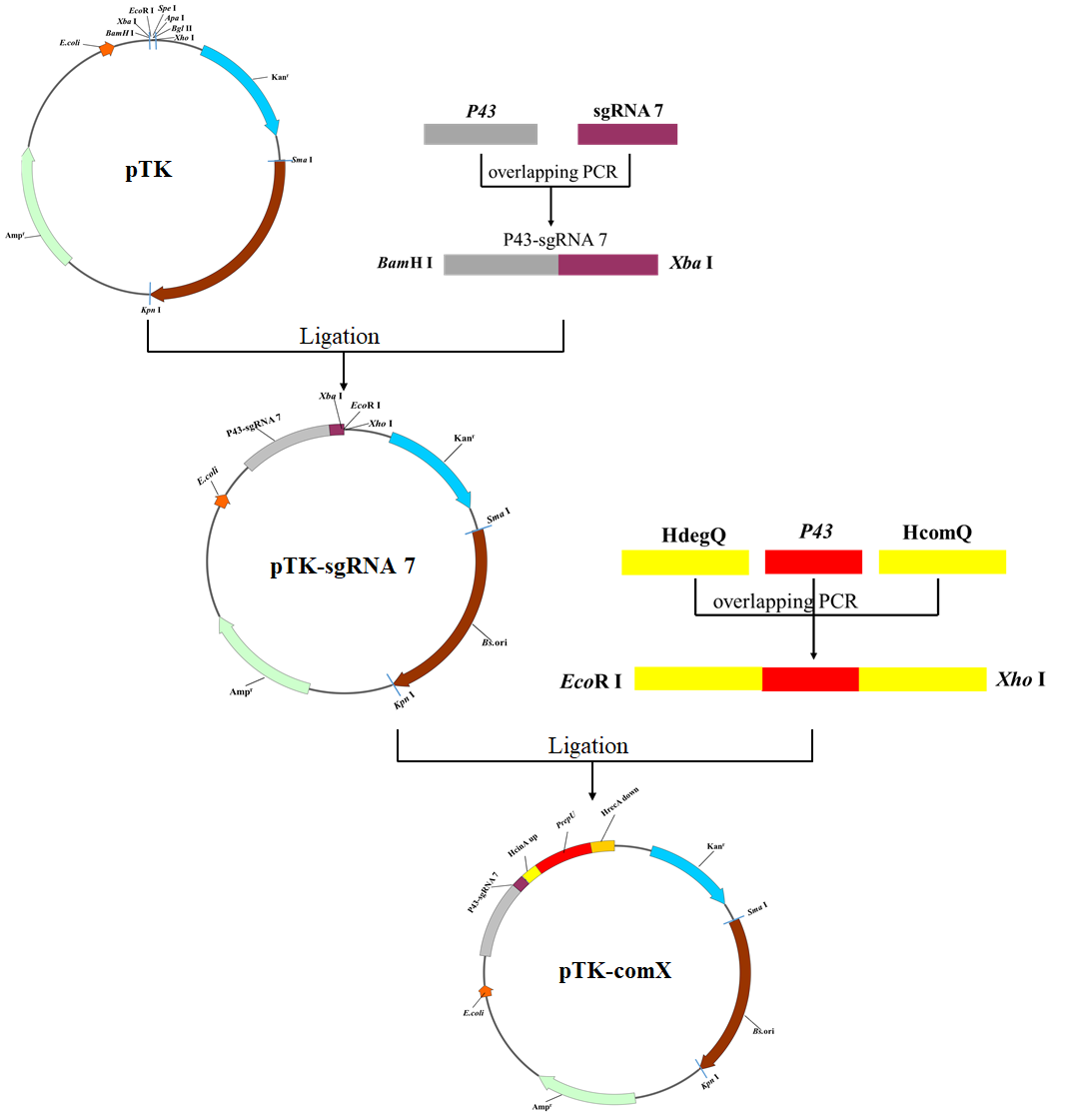


**Fig. S8 Schematic diagram of the construction process for plasmid pTK-comX**


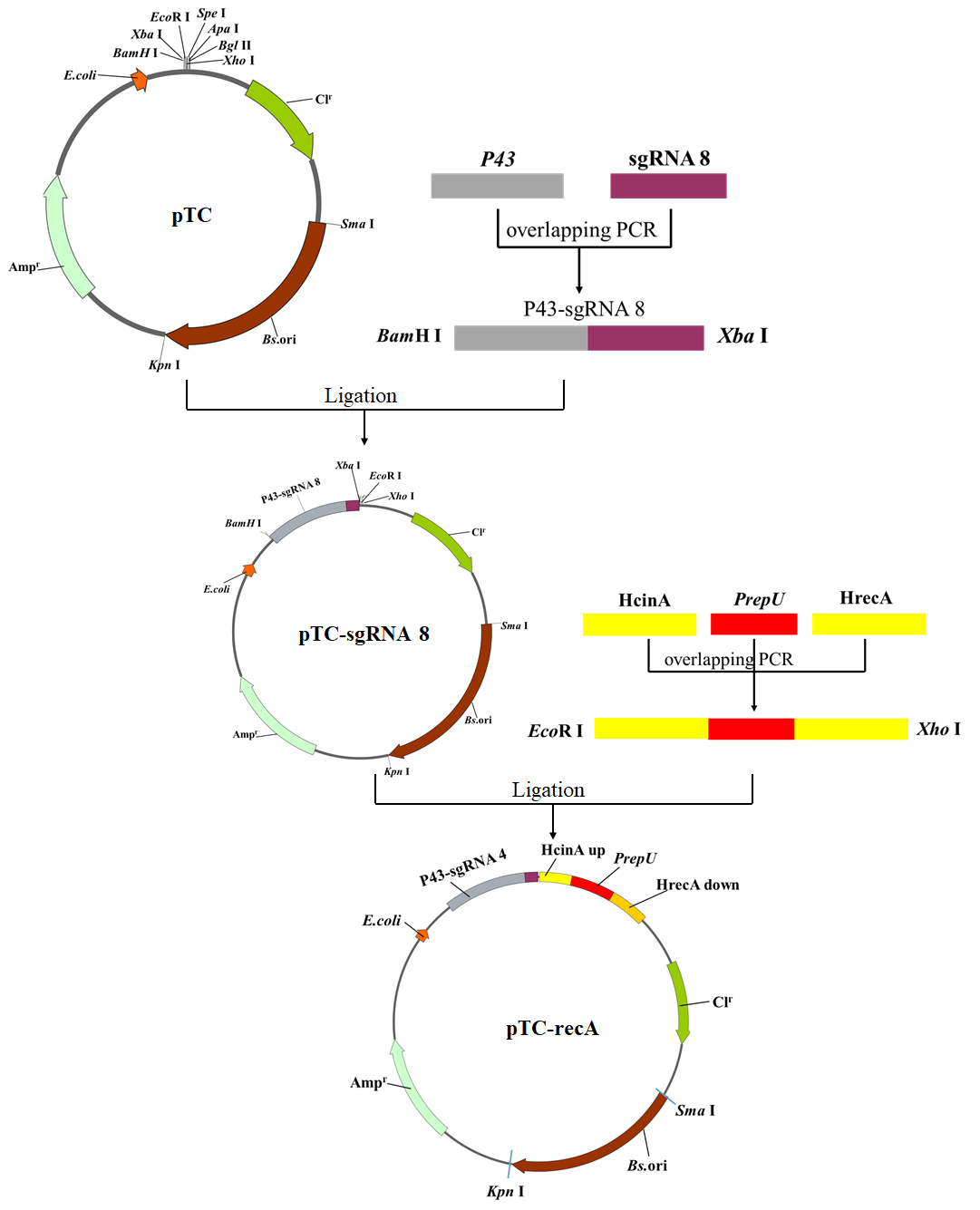


**Fig. S9 Schematic diagram of the construction process for plasmid pTC-recA**

1 GAATTCACTA GTGGGCCC**CG TAGGCGCCGA CATGCCGCCT GTTTTAGGAG ATATTATCAT**

**61 CAGCGCTGAC AGAACGAAAG AGCAGGCGGA AGAGTACGGC CATTCATTTA TGAGAGAGCT**

**121 CGGTTTTCTG GCCGTGCACG GCTTTTTGCA TTTGCTTGGC TATGATCACA TGACAAAAGA**

**181 AGAAGAGGAA GAAATGTTTT CAAAGCAAAA GGATTTGCTG GATGAGTATG GACTCACGAG**

**241 ATCATAAAAA CGAGTGGAGC AGGTTCCTGA AGAGCTTTGT CCATGCATGG AGGGGAATCT**

**301 GGAAAACGGC GCGGTCAGAG CGGAATTTTC AATTTCATAT TACCGCGGCC TGCGCCGTTA**

**361 TCGTTTGCGG TTTTCTCACC GGGCTGAGCA CAGCCGAATG GGCCGTTGTT TTGATGCTGA**

**421 TCGGAGGAAT GCTTGCTTTG GAGCTGTTAA ATACCGCGAT AGAGCATGTT GTGGATTTAG**

**481 TTACCGATCA GTATCACCCG CTCGCAAAAG CGGCAAAGGA CGCGGCCGCC GGAGCCGTTT**

**541 GCGTCTTTGC CGTGATATCG TGTATCATTG GTTTACTCAT CTTCTTGCCG AAGATATGTT**

**601 AGCAGAAGAT TCTTACAATT ATTTTACATT GCCAAAAATG GGCGTGAAAA ACCAATCATA**

**661 ATTATGTAAA ATAAAAGTGA CAGCGGTATC ATACAGGATA AAGGTAAGAG AGGAATTAAG**

**721 TACACATGGA TAAAAAATAT TCAATCGGAC TGGATATTGG CACAAACAGC GTTGGCTGGG**

**781 CGGTGATTAC AGATGAATAT AAAGTCCCGT CTAAAAAATT TAAAGTTTTA GGAAATACAG**

**841 ATCGTCATAG CATTAAAAAG AATCTTATTG GCGCATTACT GTTTGATTCT GGCGAAACAG**

**901 CGGAAGCAAC ACGTCTGAAA CGCACAGCGC GGAGAAGATA TACGCGGCGG AAAAATCGTA**

**961 TTTGCTATTT ACAAGAAATT TTTAGTAATG AAATGGCGAA AGTCGATGAT AGCTTCTTTC**

**1021 ATAGACTGGA AGAAAGCTTT TTAGTTGAAG AAGATAAAAA ACATGAACGC CATCCGATTT**

**1081 TTGGCAACAT CGTTGATGAA GTAGCATATC ATGAAAAATA TCCGACAATT TATCATCTGC**

**1141 GCAAAAAATT AGTTGATAGC ACAGATAAAG CAGATCTGAG ACTGATTTAT TTGGCGTTAG**

**1201 CACATATGAT TAAATTTCGG GGTCATTTTC TTATTGAAGG CGATCTTAAT CCTGATAATT**

**1261 CAGATGTTGA TAAATTGTTT ATTCAGTTAG TACAAACATA TAATCAACTT TTTGAAGAAA**

**1321 ACCCGATTAA CGCGAGCGGA GTTGATGCAA AAGCTATTCT GAGCGCGAGA CTTAGCAAAT**

**1381 CAAGACGGCT GGAAAATCTT ATTGCACAGC TGCCGGGCGA AAAGAAAAAT GGCTTGTTTG**

**1441 GCAATCTGAT CGCTCTGAGC TTAGGCCTGA CACCGAATTT TAAAAGCAAC TTTGATCTTG**

**1501 CTGAAGATGC TAAACTGCAA CTGTCAAAAG ATACATATGA TGATGATCTT GATAACCTGT**

**1561 TAGCACAAAT TGGCGATCAA TATGCTGATT TATTTCTGGC AGCAAAAAAT CTGTCAGATG**

**1621 CAATTTTACT TTCAGATATT CTTAGAGTCA ATACAGAAAT TACAAAAGCA CCGCTTTCAG**

**1681 CATCAATGAT TAAACGGTAT GACGAACATC ATCAAGATCT GACATTACTG AAAGCGCTGG**

**1741 TTCGCCAGCA ACTGCCTGAA AAATATAAAG AAATCTTCTT TGATCAGTCT AAAAACGGAT**

**1801 ATGCAGGATA TATTGATGGA GGAGCAAGTC AAGAAGAATT TTATAAATTT ATTAAACCGA**

**1861 TCCTTGAAAA AATGGACGGA ACAGAAGAAT TGTTAGTTAA ACTTAATAGA GAAGATTTAC**

**1921 TTCGTAAACA AAGAACATTT GATAATGGAA GCATTCCGCA TCAAATCCAT TTAGGCGAAC**

**1981 TCCATGCGAT CTTAAGACGT CAGGAAGATT TTTATCCGTT TCTGAAAGAT AATCGCGAAA**

**2041 AAATTGAAAA AATTCTTACA TTTCGGATTC CGTATTACGT TGGCCCTCTG GCGCGCGGCA**

**2101 ACAGCCGCTT TGCGTGGATG ACAAGAAAAT CAGAAGAAAC GATTACACCG TGGAACTTTG**

**2161 AAGAAGTTGT AGATAAAGGC GCTAGCGCAC AGTCATTTAT TGAAAGAATG ACAAATTTTG**

**2221 ATAAAAATCT GCCGAATGAA AAAGTCTTAC CGAAACATTC TCTGTTATAT GAATATTTTA**

**2281 CAGTGTATAA TGAACTGACG AAAGTGAAAT ATGTTACAGA AGGAATGAGA AAACCGGCAT**

**2341 TTTTATCTGG AGAACAAAAG AAAGCCATTG TTGATTTGCT GTTTAAAACA AATCGTAAAG**

**2401 TGACAGTTAA ACAGCTTAAA GAAGATTATT TTAAGAAAAT TGAATGCTTT GATTCAGTTG**

**2461 AAATTAGCGG AGTTGAAGAT AGATTTAATG CTTCATTAGG CACATATCAT GATCTTCTTA**

**2521 AAATTATTAA AGATAAAGAT TTTCTGGATA ATGAAGAAAA TGAAGATATT TTAGAAGATA**

**2581 TTGTGTTAAC ACTTACACTG TTTGAAGATA GAGAAATGAT TGAAGAAAGA CTTAAAACAT**

**2641 ATGCACACCT GTTTGATGAT AAAGTGATGA AACAACTTAA ACGTCGTAGA TATACAGGAT**

**2701 GGGGCAGATT ATCTAGAAAA TTAATTAATG GCATTAGAGA TAAACAATCA GGAAAAACGA**

**2761 TTTTAGATTT TCTGAAATCT GATGGATTTG CCAATAGAAA TTTTATGCAG TTGATTCATG**

**2821 ATGATAGCCT GACATTTAAA GAAGATATTC AAAAAGCACA AGTGAGCGGC CAAGGTGATA**

**2881 GCCTTCATGA ACATATTGCT AACTTAGCCG GCTCTCCGGC TATTAAAAAA GGAATTCTTC**

**2941 AAACAGTGAA AGTCGTAGAT GAATTAGTTA AAGTTATGGG CAGACATAAA CCGGAAAATA**

**3001 TCGTTATTGA AATGGCTAGA GAAAATCAAA CAACACAAAA AGGACAGAAA AATTCAAGAG**

**3061 AAAGAATGAA AAGAATCGAA GAAGGAATTA AAGAATTAGG CAGCCAAATT CTGAAAGAAC**

**3121 ATCCGGTTGA AAATACACAA CTGCAGAATG AAAAACTGTA TTTATATTAT CTGCAAAACG**

**3181 GAAGAGATAT GTATGTTGAT CAGGAACTTG ATATTAATAG ATTATCTGAT TATGATGTGG**

**3241 ATCATATTGT CCCGCAATCA TTTCTTAAAG ATGATTCTAT CGATAATAAA GTGCTTACAC**

**3301 GTAGCGATAA AAATCGAGGA AAATCTGATA ACGTTCCGTC TGAAGAAGTG GTTAAAAAGA**

**3361 TGAAAAATTA TTGGAGACAG TTGCTTAACG CAAAACTTAT CACACAGAGA AAATTTGATA**

**3421 ACCTGACAAA AGCCGAACGT GGCGGACTGT CAGAACTGGA TAAAGCCGGA TTTATCAAAC**

**3481 GCCAACTTGT TGAAACGAGA CAGATTACAA AACATGTTGC ACAAATTCTG GATTCTAGAA**

**3541 TGAACACGAA ATATGATGAA AACGATAAAC TTATTAGAGA AGTGAAAGTG ATTACACTTA**

**3601 AATCAAAATT AGTTTCTGAT TTTCGTAAAG ATTTTCAATT TTATAAAGTC AGAGAAATTA**

**3661 ACAATTATCA TCATGCACAT GATGCTTATT TGAATGCGGT TGTGGGCACG GCGTTAATTA**

**3721 AAAAATATCC GAAACTTGAA TCAGAATTTG TTTACGGAGA TTATAAAGTC TATGATGTGC**

**3781 GGAAAATGAT TGCAAAATCA GAACAGGAAA TCGGAAAAGC AACAGCGAAA TATTTCTTTT**

**3841 ATAGCAATAT CATGAATTTC TTTAAAACAG AAATCACCCT GGCAAATGGA GAAATCCGGA**

**3901 AACGGCCGCT GATTGAAACA AATGGCGAAA CAGGCGAAAT TGTTTGGGAT AAAGGCCGGG**

**3961 ATTTTGCCAC AGTTCGGAAA GTTTTGTCGA TGCCGCAAGT TAATATTGTG AAAAAGACAG**

**4021 AAGTACAGAC GGGCGGCTTT TCAAAAGAAT CAATTTTACC GAAAAGAAAT TCCGATAAAT**

**4081 TAATTGCACG CAAAAAAGAC TGGGATCCGA AAAAATATGG CGGCTTTGAT AGCCCTACAG**

**4141 TTGCGTATAG CGTTCTTGTG GTGGCAAAAG TGGAGAAAGG TAAAAGCAAA AAACTGAAAT**

**4201 CAGTTAAAGA ATTACTGGGC ATTACAATTA TGGAACGCTC AAGCTTTGAG AAAAATCCGA**

**4261 TCGATTTTCT GGAAGCAAAA GGCTATAAAG AAGTCAAAAA AGATCTGATT ATTAAATTGC**

**4321 CGAAATATTC ATTATTTGAA TTAGAAAACG GAAGAAAACG CATGCTGGCA AGCGCCGGCG**

**4381 AACTGCAAAA AGGCAATGAA CTGGCGCTTC CGTCTAAATA TGTCAATTTT CTTTATCTGG**

**4441 CGTCACATTA TGAAAAATTA AAAGGCAGCC CGGAAGATAA TGAACAAAAA CAACTTTTTG**

**4501 TGGAACAGCA TAAACATTAT CTTGATGAAA TCATTGAACA AATTTCAGAA TTTAGTAAAC**

**4561 GCGTCATTCT GGCGGATGCA AATCTGGATA AAGTCCTGAG CGCGTATAAT AAACATAGAG**

**4621 ATAAACCGAT TAGAGAACAA GCAGAAAATA TTATTCATTT ATTTACTTTA ACGAATCTGG**

**4681 GCGCTCCGGC AGCTTTTAAA TATTTTGACA CAACAATTGA TCGCAAAAGA TATACATCAA**

**4741 CAAAAGAAGT TCTTGATGCG ACATTAATCC ATCAGTCAAT TACAGGCCTT TATGAAACAC**

**4801 GTATTGATCT GAGCCAGCTG GGCGGCGATT AAGAGGCATC AAATAAAACG AAAGGCTCAG**

**4861 TCGAAAGACT GGGCCTTTCG TTTTATCTGT TGTTTGTCGG TGAACGCTCT CCTGAGTAGG**

**4921 ACAAATCCGC CGCTCTAGCT AAGCAGAAGG CCATCCTGAC GGATGGCCTT TTTGCGTTTC**

**4981 TACAAACTCT TGTTAACTCT AGAGCTGCCT GCCGCGTTTC GGTGATGAAG ATCTTCCCGA**

**5041 TGATTAATTA ATTCAGAACG CTCGGTTGCC GCCGGGCGTT TTTTATGCAG CAATGGCAAG**

**5101 AACGTTGCTC TAGA**CTCGAG GAGATCAGGG AATGAGTTTA TAAAATAAAA AAAGCACCTG

5161 AAAAGGTGTC TTTTTTTGAT GGTTTTGAAC TTGTTCTTTC TTATCTTGAT ACATATAGAA

5221 ATAACGTCAT TTTTATTTTA GTTGCTGAAA GGTGCGTTGA AGTGTTGGTA TGTATGTGTT

5281 TTAAAGTATT GAAAACCCTT AAAATTGGTT GCACAGAAAA ACCCCATCTG TTAAAGTTAT

5341 AAGTGACTAA ACAAATAACT AAATAGATGG GGGTTTCTTT TAATATTATG TGTCCTAATA

5401 GTAGCATTTA TTCAGATGAA AAATCAAGGG TTTTAGTGGA CAAGACAAAA AGTGGAAAAG

5461 TGAGACCATG ATGCTTAGGA AGACGAGTTA TTAATAGCTG AATAAGAACG GTGCTCTCCA

5521 AATATTCTTA TTTAGAAAAG CAAATCTAAA ATTATCTGAA AAGGGAATGA GAATA**GTGAA**

**5581 TGGACCAATA ATAATGACTA GAGAAGAAAG AATGAAGATT GTTCATGAAA TTAAGGAACG**

**5641 AATATTGGAT AAATATGGGG ATGATGTTAA GGCTATTGGT GTTTATGGCT CTCTTGGTCG**

**5701 TCAGACTGAT GGGCCCTATT CGGATATTGA GATGATGTGT GTCATGTCAA CAGAGGAAGC**

**5761 AGAGTTCAGC CATGAATGGA CAACCGGTGA GTGGAAGGTG GAAGTGAATT TTGATAGCGA**

**5821 AGAGATTCTA CTAGATTATG CATCTCAGGT GGAATCAGAT TGGCCGCTTA CACATGGTCA**

**5881 ATTTTTCTCT ATTTTGCCGA TTTATGATTC AGGTGGATAC TTAGAGAAAG TGTATCAAAC**

**5941 TGCTAAATCG GTAGAAGCCC AAACGTTCCA CGATGCGATT TGTGCCCTTA TCGTAGAAGA**

**6001 GCTGTTTGAA TATGCAGGCA AATGGCGTAA TATTCGTGTG CAAGGACCGA CAACATTTCT**

**6061 ACCATCCTTG ACTGTACAGG TAGCAATGGC AGGTGCCATG TTGATTGGTC TGCATCATCG**

**6121 CATCTGTTAT ACGACGAGCG CTTCGGTCTT AACTGAAGCA GTTAAGCAAT CAGATCTTCC**

**6181 TTCAGGTTAT GACCATCTGT GCCAGTTCGT AATGTCTGGT CAACTTTCCG ACTCTGAGAA**

**6241 ACTTCTGGAA TCGCTAGAGA ATTTCTGGAA TGGGATTCAG GAGTGGACAG AACGACACGG**

**6301 ATATATAGTG GATGTGTCAA AACGCATACC ATTTTGA**ACG ATGACCTCTA ATAATTGTTA

6361 ATCATGTTGG TTACGTATTT ATTAACTTCT CCTAGTATTA GTAATTATCC TGCAG**GTCCA**

**6421 GAAGGTCGAT AGAAAGCGTG AGAAACAGCG TACAGACGAT TTAGAGATGT AGAGGTACTT**

**6481 TTATGCCGAG AAAACTTTTT GCGTGTGACA GTCCTTAAAA TATACTTAGA GCGTAAGCGA**

**6541 AAGTAGTAGC GACAGCTATT AACTTTCGGT TGCAAAGCTC TAGGATTTTT AATGGACGCA**

**6601 GCGCATCACA CGCAAAAAGG AAATTGGAAT AAATGCGAAA TTTGAGATGT TAATTAAAGA**

**6661 CCTTTTTGAG GTCTTTTTTT CTTAGATTTT TGGGGTTATT TAGGGGAGAA AACATAGGGG**

**6721 GGTACTACGA CCTCCCCCCT AGGTGTCCAT TGTCCATTGT CCAAACAAAT AAATAAATAT**

**6781 TGGGTTTTTA ATGTTAAAAG GTTGTTTTTT ATGTTAAAGT GAAAAAAACA GATGTTGGGA**

**6841 GGTACAGTGA TGGTTGTAGA TAGAAAAGAA GAGAAAAAAG TTGCTGTTAC TTTAAGACTT**

**6901 ACAACAGAAG AAAATGAGAT ATTAAATAGA ATCAAAGAAA AATATAATAT TAGCAAATCA**

**6961 GATGCAACCG GTATTCTAAT AAAAAAATAT GCAAAGGAGG AATACGGTGC ATTTTAAACA**

**7021 AAAAAAGATA GACAGCACTG GCATGCTGCC TATCTATGAC TAAATTTTGT TAAGTGTATT**

**7081 AGCACCGTTA TTATATCATG AGCGAAAATG TAATAAAAGA AACTGAAAAC AAGAAAAATT**

**7141 CAAGAGGACG TAATTGGACA TTTGTTTTAT ATCCAGAATC AGCAAAAGCC GAGTGGTTAG**

**7201 AGTATTTAAA AGAGTTACAC ATTCAATTTG TAGTGTCTCC ATTACATGAT AGGGATACTG**

**7261 ATACAGAAGG TAGGATGAAA AAAGAGCATT ATCATATTCT AGTGATGTAT GAGGGTAATA**

**7321 AATCTTATGA ACAGATAAAA ATAATTAACA GAAGAATTGA ATGCGACTAT TCCGCAGATT**

**7381 GCAGGAAGTG TGAAAGGTCT TGTGAGATAT ATGCTTCACA TGGACGATCC TAATAAATTT**

**7441 AAATATCAAA AAGAAGATAT GATAGTTTAT GGCGGTGTAG ATGTTGATGA ATTATTAAAG**

**7501 AAAACAACAA CAGATAGATA TAAATTAATT AAAGAAATGA TTGAGTTTAT TGATGAACAA**

**7561 GGAATCGTAG AATTTAAGAG TTTAATGGAT TATGCAATGA AGTTTAAATT TGATGATTGG**

**7621 TTCCCGCTTT TATGTGATAA CTCGGCGTAT GTTATTCAAG AATATATAAA ATCAAATCGG**

**7681 TATAAATCTG ACCGATAGAT TTTGAATTTA GGTGTCACAA GACACTCTTT TTTCGCACCA**

**7741 GCGAAAACTG GTTTAAGCCG ACTGCGCAAA AGACATAATC GATTCACAAA AAATAGGCAC**

**7801 ACGAAAAACA AGTTAAGGGA TGCAGTTTAT GCATCCCTTA ACGGTACC**AC TGGCCGTCGT

7861 TTTACAACGT CGTGACTGGG AAAACCCTGG CGTTACCCAA CTTAATCGCC TTGCAGCACA

7921 TCCCCCTTTC GCCAGCTGGC GTAATAGCGA AGAGGCCCGC ACCGATCGCC CTTCCCAACA

7981 GTTGCGCAGC CTGAATGGCG AATGGCGCCT GATGCGGTAT TTTCTCCTTA CGCATCTGTG

8041 CGGTATTTCA CACCGCATAT GGTGCACTCT CAGTACAATC TGCTCTGATG CCGCATAGTT

8101 AAGCCAGCCC CGACACCCGC CAACACCCGC TGACGCGCCC TGACGGGCTT GTCTGCTCCC

8161 GGCATCCGCT TACAGACAAG CTGTGACCGT CTCCGGGAGC TGCATGTGTC AGAGGTTTTC

8221 ACCGTCATCA CCGAAACGCG CGAGACGAAA GGGCCTCGTG ATACGCCTAT TTTTATAGGT

8281 TAATGTCATG ATAATAATGG TTTCTTAGAC GTCAGGTGGC ACTTTTCGGG GAAATGTGCG

8341 CGGAACCCCT ATTTGTTTAT TTTTCTAAAT ACATTCAAAT ATGTATCCGC TCATGAGACA

8401 ATAACCCTGA TAAATGCTTC AATAATATTG AAAAAGGAAG AGT**ATGAGTA TTCAACATTT**

**8461 CCGTGTCGCC CTTATTCCCT TTTTTGCGGC ATTTTGCCTT CCTGTTTTTG CTCACCCAGA**

**8521 AACGCTGGTG AAAGTAAAAG ATGCTGAAGA TCAGTTGGGT GCACGAGTGG GTTACATCGA**

**8581 ACTGGATCTC AACAGCGGTA AGATCCTTGA GAGTTTTCGC CCCGAAGAAC GTTTTCCAAT**

**8641 GATGAGCACT TTTAAAGTTC TGCTATGTGG CGCGGTATTA TCCCGTATTG ACGCCGGGCA**

**8701 AGAGCAACTC GGTCGCCGCA TACACTATTC TCAGAATGAC TTGGTTGAGT ACTCACCAGT**

**8761 CACAGAAAAG CATCTTACGG ATGGCATGAC AGTAAGAGAA TTATGCAGTG CTGCCATAAC**

**8821 CATGAGTGAT AACACTGCGG CCAACTTACT TCTGACAACG ATCGGAGGAC CGAAGGAGCT**

**8881 AACCGCTTTT TTGCACAACA TGGGGGATCA TGTAACTCGC CTTGATCGTT GGGAACCGGA**

**8941 GCTGAATGAA GCCATACCAA ACGACGAGCG TGACACCACG ATGCCTGTAG CAATGGCAAC**

**9001 AACGTTGCGC AAACTATTAA CTGGCGAACT ACTTACTCTA GCTTCCCGGC AACAATTAAT**

**9061 AGACTGGATG GAGGCGGATA AAGTTGCAGG ACCACTTCTG CGCTCGGCCC TTCCGGCTGG**

**9121 CTGGTTTATT GCTGATAAAT CTGGAGCCGG TGAGCGTGGG TCTCGCGGTA TCATTGCAGC**

**9181 ACTGGGGCCA GATGGTAAGC CCTCCCGTAT CGTAGTTATC TACACGACGG GGAGTCAGGC**

**9241 AACTATGGAT GAACGAAATA GACAGATCGC TGAGATAGGT GCCTCACTGA TTAAGCATTG**

**9301 GTAA**CTGTCA GACCAAGTTT ACTCATATAT ACTTTAGATT GATTTAAAAC TTCATTTTTA

9361 ATTTAAAAGG ATCTAGGTGA AGATCCTTTT TGATAATCTC ATGACCAAAA TCCCTTAACG

9421 TGAGTTTTCG TTCCACTGAG CGTCAGACCC CGTAGAAAAG ATCAAAGGAT CTTCTTGAGA

9481 TCCTTTTTTT CTGCGCGTAA TCTGCTGCTT GCAAACAAAA AAACCACCGC TACCAGCGGT

9541 GGTTTGTTTG CCGGATCAAG AGCTACCAAC TCTTTTTCCG AAGGTAACTG GCTTCAGCAG

9601 AGCGCAGATA CCAAATACTG TTCTTCTAGT GTAGCCGTAG TTAGGCCACC ACTTCAAGAA

9661 CTCTGTAGCA CCGCCTACAT ACCTCGCTCT GCTAATCCTG TTACCAGTGG CTGCTGCCAG

9721 TGGCGATAAG TCGTGTCTTA CCGGGTTGGA CTCAAGACGA TAGTTACCGG ATAAGGCGCA

9781 GCGGTCGGGC TGAACGGGGG GTTCGTGCAC ACAGCCCAGC TTGGAGCGAA CGACCTACAC

9841 CGAACTGAGA TACCTACAGC GTGAGCTATG AGAAAGCGCC ACGCTTCCCG AAGGGAGAAA

9901 GGCGGACAGG TATCCGGTAA GCGGCAGGGT CGGAACAGGA GAGCGCACGA GGGAGCTTCC

9961 AGGGGGAAAC GCCTGGTATC TTTATAGTCC TGTCGGGTTT CGCCACCTCT GACTTGAGCG

10021 TCGATTTTTG TGATGCTCGT CAGGGGGGCG GAGCCTATGG AAAAACGCCA GCAACGCGGC

10081 CTTTTTACGG TTCCTGGCCT TTTGCTGGCC TTTTGCTCAC ATGTTCTTTC CTGCGTTATC

10141 CCCTGA**TTCT GTGGATAACC GTATTACCGC CTTTGAGTGA GCTGATACCG CTCGCCGCAG**

**10201 CCGAACGACC GAGCGCAGCG AGTCAGTGAG CGAGGAAGCG GAAGA**GCGCC CAATACGCAA

10261 ACCGCCTCTC CCCGCGCGTT GGCCGATTCA TTAATGCAGC TGGCACGACA GGTTTCCCGA

10321 CTGGAAAGCG GGCAGTGAGC GCAACGCAAT TAATGTGAGT TAGCTCACTC ATTAGGCACC

10381 CCAGGCTTTA CACTTTATGC TTCCGGCTCG TATGTTGTGT GGAATTGTGA GCGGATAACA

10441 ATTTCACACA GGAAACAGCT ATGACCATGA TTACGCCAAG CTTGCATGCC CGCGGGGATC

10501 CCCATGGTCT AGA

**Fig S10** **The sequence of pTN-Cas9 with a nucleotide length of 10513 bp**. The bolded segments marked in gray, red, black, blue, yellow, orange and purple represent gene sequences of *P43* promoter from Bv916 (CP009611), *Cas9*, transcription terminator *T1T2*, *Neo^r^*, thermosensitive replication origin for *Bacillus*, *Amp^r^* and replication origin for *Escherichia coli*, respectively.

1 **CGTAGGCGCC GACATGCCGC CTGTTTTAGG AGATATTATC ATCAGCGCTG ACAGAACGAA**

**61 AGAGCAGGCG GAAGAGTACG GCCATTCATT TATGAGAGAG CTCGGTTTTC TGGCCGTGCA**

**121 CGGCTTTTTG CATTTGCTTG GCTATGATCA CATGACAAAA GAAGAAGAGG AAGAAATGTT**

**181 TTCAAAGCAA AAGGATTTGC TGGATGAGTA TGGACTCACG AGATCATAAA AACGAGTGGA**

**241 GCAGGTTCCT GAAGAGCTTT GTCCATGCAT GGAGGGGAAT CTGGAAAACG GCGCGGTCAG**

**301 AGCGGAATTT TCAATTTCAT ATTACCGCGG CCTGCGCCGT TATCGTTTGC GGTTTTCTCA**

**361 CCGGGCTGAG CACAGCCGAA TGGGCCGTTG TTTTGATGCT GATCGGAGGA ATGCTTGCTT**

**421 TGGAGCTGTT AAATACCGCG ATAGAGCATG TTGTGGATTT AGTTACCGAT CAGTATCACC**

**481 CGCTCGCAAA AGCGGCAAAG GACGCGGCCG CCGGAGCCGT TTGCGTCTTT GCCGTGATAT**

**541 CGTGTATCAT TGGTTTACTC ATCTTCTTGC CGAAGATATG TTAGCAGAAG ATTCTTACAA**

**601 TTATTTTACA TTGCCAAAAA TGGGCGTGAA AAACCAATCA TAATTATGTA AAATAAAATT**

**661 CCTGTTATCT AATAGCCCAA AATCTCGATC TTTATCGTTC AATTTTATTC CGATCAGGCA**

**721 ATAGTTGAAC TTTTTCACCG TGGCTCAGCC ACGAAAAAAA** GAATTC**TGAT CAGCCGGATG**

**781 ACCAATCGCA CGGTGCAATT GAATCAGGGA GGTCCGGAAT CGAAAAGAGG AAAGCTTCAC**

**841 GAAACAGGGG ATGATTTTCT CGTGCTGGAA ACGGAAGATG ACGGAATTGT ATATTTTAAC**

**901 GCCGACCATG TCAAAAGCAT CAGCGCGGAA CAAGAAGAGG ACGGCCAAGA GGAGGAGCGC**

**961 CCGGAATTTG AAATGGCTGA TGATTTTCAC GGCATATTTA AACGCCTGAT CCACAAATGG**

**1021 GTATCCATCA ACCGCGGAGG TCCTGAAGCC GTTGAAGGTA TACTTGTGGA TAATTCCGAC**

**1081 GGCCACTACA CTCTTGTGAA AGATAAAGAA GTGCTGCGGA TTTATCCTTT TCATATCAAG**

**1141 AGCATCAGCG AAGGGGCAAA AGGAGCAGCT AAAAAAGAGG AGAATAAAGA CGAAGAGAAT**

**1201 AAAGGTGAGA AAGAGAGCGC CGAAGAAGAA AAGCCGCATG AAGAATCACG CGAAGAGCAG**

**1261 CGTACTTCCT CTAAAAAGTC GAAAAGATCA TCAAGATCCG TTCGATCCTC AAAACGGGAG**

**1321 AAAGAGGTCT CTTATAGCTA CGCCACTGTA TTAAGGACAA TAGATTATCG GTGGAAACAC**

**1381 GGCCGTAAA**G GTGGTGGTGG TGGTAAA**ATG GTTTCTAAAG GTGAAGAATT ATTCACTGGT**

**1441 GTTGTTCCAA TCTTAGTTGA ATTAGATGGT GATGTAAACG GTCATAAATT CTCTGTTTCT**

**1501 GGTGAAGGTG AAGGTGATGC AACATACGGT AAATTAACTT TAAAATTCAT CTGTACTACT**

**1561 GGTAAATTAC CAGTTCCATG GCCAACTTTA GTTACTACTT TAACTTACGG TGTACAATGT**

**1621 TTCTCTCGTT ACCCAGATCA CATGAAACAA CATGATTTCT TCAAATCTGC TATGCCAGAA**

**1681 GGTTACGTTC AAGAACGTAC TATCTTCTTC AAAGATGATG GTAACTACAA AACTCGTGCT**

**1741 GAAGTAAAAT TCGAGGGTGA CACTCTTGTA AACCGTATCG AATTAAAAGG TATCGATTTC**

**1801 AAAGAAGATG GTAACATCTT AGGACACAAA CTTGAATACA ACTACAACTC TCACAACGTA**

**1861 TACATCATGG CAGATAAACA AAAGAACGGT ATCAAAGTAA ACTTCAAAAT CCGTCACAAC**

**1921 ATCGAGGACG GTTCTGTTCA ATTAGCTGAT CACTACCAAC AAAACACACC AATCGGAGAT**

**1981 GGTCCAGTAT TACTTCCAGA CAACCACTAC TTATCTACTC AATCTGCATT ATCTAAAGAT**

**2041 CCAAACGAAA AACGTGATCA CATGGTTTTA TTAGAATTTG TTACTGCTGC TGGTATCACT**

**2101 TTAGGTATGG ATGAATTATA CAAATAATTG GAAAGATTGT CCGTAAACGC GGGAGAGGGG**

**2161 GGAATGAAAT GGCTGGATTA AGCAGATTTC TGGATAAACA AGTCGATATT GAGATATCGG**

**2221 GGAATACGAC ATTTTCAGGA ACTCTTTTGG ATATCGGTCA GGATATCATT GTGATTCATG**

**2281 ACGGCCGGAC GTTTCTTTAT ATTCCGCTGC TGCATCTTCA GAGGATGACA TTGACGCTCC**

**2341 CGGACGAAGA GAATAAAGAA CCGTACGCAA GAAAAAAAAG TCCGCATCCC GAAAAACAGG**

**2401 AACATTCCTT TTCTTATCGG AATACCCTCC AGCATATAAA AGGCAGATTC ACAGAAATAT**

**2461 TTGTTACGGG AGACCGTTCC ATTCATGGGT ATGTGACAAG TGTTTTAAAT GATTATTTTG**

**2521 TTTTCTTCTC TCCGGTTTAT AAGACTCTTT TCATCTCGAT GCATCATTTG AAATGGCTGA**

**2581 CACCCTATTC CGATGAGCAG ACGCCGTATA CATTAAACAG TTCAGAGCTG CCGGTTGTTC**

**2641 CCGCTAATGT GCCCCTTGTC CGGAATTTTG AGGAGCAATT AAAAAGATAT ATCGGGAAGC**

**2701 TGATTATTCT GGATTTAG**CT CGAGTAACAG GTTGGCTGAT AAGTCCCCGG TCTGCCACAT

2761 AGATGGCGTC GCTAGTATTA AATGCATATT ATTTTTATAT AGTACCAACC TTCAAATGAT

2821 TCCCTATAGC TTGTAAATTC TATCATAATT GTGGTTTCAA AATCGGCTCC GTCGATACTA

2881 TGTTATACGC CAACTTTGAA AACAACTTTG AAAAAGCTGT TTTCTGGTAT TTAAGGTTTT

2941 AGAATGCAAG GAACAGTGAA TTGGAGTTCG TCTTGTTATA ATTAGCTTCT TGGGGTATCT

3001 TTAAATACTG TAGAAAAGAG GAAGGAAATA ATAA**ATGGCT AAAATGAGAA TATCACCGGA**

**3061 ATTGAAAAAA CTGATCGAAA AATACCGCTG CGTAAAAGAT ACGGAAGGAA TGTCTCCTGC**

**3121 TAAGGTATAT AAGCTGGTGG GAGAAAATGA AAACCTATAT TTAAAAATGA CGGACAGCCG**

**3181 GTATAAAGGG ACCACCTATG ATGTAGAACG GGAAAAGGAC ATGATGCTAT GGCTGGAAGG**

**3241 AAAGCTGCCT GTTCCAAAGG TCCTGCACTT TGAACGGCAT GATGGCTGGA GCAATCTGCT**

**3301 CATGAGTGAG GCCGATGGCG TCCTTTGCTC GGAAGAGTAT GAAGATGAAC AAAGCCCTGA**

**3361 AAAGATTATC GAGCTGTATG CGGAGTGCAT CAGGCTCTTT CACTCCATCG ACATATCGGA**

**3421 TTGTCCCTAT ACGAATAGCT TAGACAGCCG CTTAGCCGAA TTGGATTACT TACTGAATAA**

**3481 CGATCTGGCC GATGTGGATT GCGAAAACTG GGAAGAAGAC ACTCCATTTA AAGATCCGCG**

**3541 CGAGCTGTAT GATTTTTTAA AGACGGAAAA GCCCGAAGAG GAACTTGTCT TTTCCCACGG**

**3601 CGACCTGGGA GACAGCAACA TCTTTGTGAA AGATGGCAAA GTAAGTGGCT TTATTGATCT**

**3661 TGGGAGAAGC GGCAGGGCGG ACAAGTGGTA TGACATTGCC TTCTGCGTCC GGTCGATCAG**

**3721 GGAGGATATC GGGGAAGAAC AGTATGTCGA GCTATTTTTT GACTTACTGG GGATCAAGCC**

**3781 TGATTGGGAG AAAATAAAAT ATTATATTTT ACTGGATGAA TTGTTTTAG**T ACCTAGAATT

3841 TAGATGTCTA AAAAGCTTTA ACTACAAGCT TTTTAGACAT CTAATCTTTT CTGAAGTACA

3901 TCCGCAACTG TCCATACTCT GATGTTTTAT ATCTTTTCTA AAAGTTCGCT AGATAGGGGT

3961 CCCGAGCGCC TACGAGGAAT TTGTATCGCC CGGG **GTCCAG AAGGTCGATA GAAAGCGTGA**

**4021 GAAACAGCGT ACAGACGATT TAGAGATGTA GAGGTACTTT TATGCCGAGA AAACTTTTTG**

**4081 CGTGTGACAG TCCTTAAAAT ATACTTAGAG CGTAAGCGAA AGTAGTAGCG ACAGCTATTA**

**4141 ACTTTCGGTT GCAAAGCTCT AGGATTTTTA ATGGACGCAG CGCATCACAC GCAAAAAGGA**

**4201 AATTGGAATA AATGCGAAAT TTGAGATGTT AATTAAAGAC CTTTTTGAGG TCTTTTTTTC**

**4261 TTAGATTTTT GGGGTTATTT AGGGGAGAAA ACATAGGGGG GTACTACGAC CTCCCCCCTA**

**4321 GGTGTCCATT GTCCATTGTC CAAACAAATA AATAAATATT GGGTTTTTAA TGTTAAAAGG**

**4381 TTGTTTTTTA TGTTAAAGTG AAAAAAACAG ATGTTGGGAG GTACAGTGAT GGTTGTAGAT**

**4441 AGAAAAGAAG AGAAAAAAGT TGCTGTTACT TTAAGACTTA CAACAGAAGA AAATGAGATA**

**4501 TTAAATAGAA TCAAAGAAAA ATATAATATT AGCAAATCAG ATGCAACCGG TATTCTAATA**

**4561 AAAAAATATG CAAAGGAGGA ATACGGTGCA TTTTAAACAA AAAAAGATAG ACAGCACTGG**

**4621 CATGCTGCCT ATCTATGACT AAATTTTGTT AAGTGTATTA GCACCGTTAT TATATCATGA**

**4681 GCGAAAATGT AATAAAAGAA ACTGAAAACA AGAAAAATTC AAGAGGACGT AATTGGACAT**

**4741 TTGTTTTATA TCCAGAATCA GCAAAAGCCG AGTGGTTAGA GTATTTAAAA GAGTTACACA**

**4801 TTCAATTTGT AGTGTCTCCA TTACATGATA GGGATACTGA TACAGAAGGT AGGATGAAAA**

**4861 AAGAGCATTA TCATATTCTA GTGATGTATG AGGGTAATAA ATCTTATGAA CAGATAAAAA**

**4921 TAATTAACAG AAGAATTGAA TGCGACTATT CCGCAGATTG CAGGAAGTGT GAAAGGTCTT**

**4981 GTGAGATATA TGCTTCACAT GGACGATCCT AATAAATTTA AATATCAAAA AGAAGATATG**

**5041 ATAGTTTATG GCGGTGTAGA TGTTGATGAA TTATTAAAGA AAACAACAAC AGATAGATAT**

**5101 AAATTAATTA AAGAAATGAT TGAGTTTATT GATGAACAAG GAATCGTAGA ATTTAAGAGT**

**5161 TTAATGGATT ATGCAATGAA GTTTAAATTT GATGATTGGT TCCCGCTTTT ATGTGATAAC**

**5221 TCGGCGTATG TTATTCAAGA ATATATAAAA TCAAATCGGT ATAAATCTGA CCGATAGATT**

**5281 TTGAATTTAG GTGTCACAAG ACACTCTTTT TTCGCACCAG CGAAAACTGG TTTAAGCCGA**

**5341 CTGCGCAAAA GACATAATCG ATTCACAAAA AATAGGCACA CGAAAAACAA GTTAAGGGAT**

**5401 GCAGTTTATG CATCCCTTAA C**GGTACCACT GGCCGTCGTT TTACAACGTC GTGACTGGGA

5461 AAACCCTGGC GTTACCCAAC TTAATCGCCT TGCAGCACAT CCCCCTTTCG CCAGCTGGCG

5521 TAATAGCGAA GAGGCCCGCA CCGATCGCCC TTCCCAACAG TTGCGCAGCC TGAATGGCGA

5581 ATGGCGCCTG ATGCGGTATT TTCTCCTTAC GCATCTGTGC GGTATTTCAC ACCGCATATG

5641 GTGCACTCTC AGTACAATCT GCTCTGATGC CGCATAGTTA AGCCAGCCCC GACACCCGCC

5701 AACACCCGCT GACGCGCCCT GACGGGCTTG TCTGCTCCCG GCATCCGCTT ACAGACAAGC

5761 TGTGACCGTC TCCGGGAGCT GCATGTGTCA GAGGTTTTCA CCGTCATCAC CGAAACGCGC

5821 GAGACGAAAG GGCCTCGTGA TACGCCTATT TTTATAGGTT AATGTCATGA TAATAATGGT

5881 TTCTTAGACG TCAGGTGGCA CTTTTCGGGG AAATGTGCGC GGAACCCCTA TTTGTTTATT

5941 TTTCTAAATA CATTCAAATA TGTATCCGCT CATGAGACAA TAACCCTGAT AAATGCTTCA

6001 ATAATATTGA AAAAGGAAGA GT**ATGAGTAT TCAACATTTC CGTGTCGCCC TTATTCCCTT**

**6061 TTTTGCGGCA TTTTGCCTTC CTGTTTTTGC TCACCCAGAA ACGCTGGTGA AAGTAAAAGA**

**6121 TGCTGAAGAT CAGTTGGGTG CACGAGTGGG TTACATCGAA CTGGATCTCA ACAGCGGTAA**

**6181 GATCCTTGAG AGTTTTCGCC CCGAAGAACG TTTTCCAATG ATGAGCACTT TTAAAGTTCT**

**6241 GCTATGTGGC GCGGTATTAT CCCGTATTGA CGCCGGGCAA GAGCAACTCG GTCGCCGCAT**

**6301 ACACTATTCT CAGAATGACT TGGTTGAGTA CTCACCAGTC ACAGAAAAGC ATCTTACGGA**

**6361 TGGCATGACA GTAAGAGAAT TATGCAGTGC TGCCATAACC ATGAGTGATA ACACTGCGGC**

**6421 CAACTTACTT CTGACAACGA TCGGAGGACC GAAGGAGCTA ACCGCTTTTT TGCACAACAT**

**6481 GGGGGATCAT GTAACTCGCC TTGATCGTTG GGAACCGGAG CTGAATGAAG CCATACCAAA**

**6541 CGACGAGCGT GACACCACGA TGCCTGTAGC AATGGCAACA ACGTTGCGCA AACTATTAAC**

**6601 TGGCGAACTA CTTACTCTAG CTTCCCGGCA ACAATTAATA GACTGGATGG AGGCGGATAA**

**6661 AGTTGCAGGA CCACTTCTGC GCTCGGCCCT TCCGGCTGGC TGGTTTATTG CTGATAAATC**

**6721 TGGAGCCGGT GAGCGTGGGT CTCGCGGTAT CATTGCAGCA CTGGGGCCAG ATGGTAAGCC**

**6781 CTCCCGTATC GTAGTTATCT ACACGACGGG GAGTCAGGCA ACTATGGATG AACGAAATAG**

**6841 ACAGATCGCT GAGATAGGTG CCTCACTGAT TAAGCATTGG TAA**CTGTCAG ACCAAGTTTA

6901 CTCATATATA CTTTAGATTG ATTTAAAACT TCATTTTTAA TTTAAAAGGA TCTAGGTGAA

6961 GATCCTTTTT GATAATCTCA TGACCAAAAT CCCTTAACGT GAGTTTTCGT TCCACTGAGC

7021 GTCAGACCCC GTAGAAAAGA TCAAAGGATC TTCTTGAGAT CCTTTTTTTC TGCGCGTAAT

7081 CTGCTGCTTG CAAACAAAAA AACCACCGCT ACCAGCGGTG GTTTGTTTGC CGGATCAAGA

7141 GCTACCAACT CTTTTTCCGA AGGTAACTGG CTTCAGCAGA GCGCAGATAC CAAATACTGT

7201 TCTTCTAGTG TAGCCGTAGT TAGGCCACCA CTTCAAGAAC TCTGTAGCAC CGCCTACATA

7261 CCTCGCTCTG CTAATCCTGT TACCAGTGGC TGCTGCCAGT GGCGATAAGT CGTGTCTTAC

7321 CGGGTTGGAC TCAAGACGAT AGTTACCGGA TAAGGCGCAG CGGTCGGGCT GAACGGGGGG

7381 TTCGTGCACA CAGCCCAGCT TGGAGCGAAC GACCTACACC GAACTGAGAT ACCTACAGCG

7441 TGAGCTATGA GAAAGCGCCA CGCTTCCCGA AGGGAGAAAG GCGGACAGGT ATCCGGTAAG

7501 CGGCAGGGTC GGAACAGGAG AGCGCACGAG GGAGCTTCCA GGGGGAAACG CCTGGTATCT

7561 TTATAGTCCT GTCGGGTTTC GCCACCTCTG ACTTGAGCGT CGATTTTTGT GATGCTCGTC

7621 AGGGGGGCGG AGCCTATGGA AAAACGCCAG CAACGCGGCC TTTTTACGGT TCCTGGCCTT

7681 TTGCTGGCCT TTTGCTCACA TGTTCTTTCC TGCGTTATCC CCTGA**TTCTG TGGATAACCG**

**7741 TATTACCGCC TTTGAGTGAG CTGATACCGC TCGCCGCAGC CGAACGACCG AGCGCAGCGA**

**7801 GTCAGTGAGC GAGGAAGCGG AAGA**GCGCCC AATACGCAAA CCGCCTCTCC CCGCGCGTTG

7861 GCCGATTCAT TAATGCAGCT GGCACGACAG GTTTCCCGAC TGGAAAGCGG GCAGTGAGCG

7921 CAACGCAATT AATGTGAGTT AGCTCACTCA TTAGGCACCC CAGGCTTTAC ACTTTATGCT

7981 TCCGGCTCGT ATGTTGTGTG GAATTGTGAG CGGATAACAA TTTCACACAG GAAACAGCTA

8041 TGACCATGAT TACGCCAAGC TTGCATGCCC GCGGGGATCC

**Fig S11** **The sequence of pTK-CotB-GFP with a nucleotide length of 8080 bp**. The bolded segments marked in gray, black, dark blue, green, blue, yellow, orange and purple represent gene sequences of *P43* promoter from Bv916 (CP009611), *sgRNA,* *CotB target,* *gfp*, *Kan^r^*, thermosensitive replication origin for *Bacillus*, *Amp^r^* and replication origin for *Escherichia coli*, respectively.

1 GGATCC**CGTA GGCGCCGACA TGCCGCCTGT TTTAGGAGAT ATTATCATCA GCGCTGACAG**

**61 AACGAAAGAG CAGGCGGAAG AGTACGGCCA TTCATTTATG AGAGAGCTCG GTTTTCTGGC**

**121 CGTGCACGGC TTTTTGCATT TGCTTGGCTA TGATCACATG ACAAAAGAAG AAGAGGAAGA**

**181 AATGTTTTCA AAGCAAAAGG ATTTGCTGGA TGAGTATGGA CTCACGAGAT CATAAAAACG**

**241 AGTGGAGCAG GTTCCTGAAG AGCTTTGTCC ATGCATGGAG GGGAATCTGG AAAACGGCGC**

**301 GGTCAGAGCG GAATTTTCAA TTTCATATTA CCGCGGCCTG CGCCGTTATC GTTTGCGGTT**

**361 TTCTCACCGG GCTGAGCACA GCCGAATGGG CCGTTGTTTT GATGCTGATC GGAGGAATGC**

**421 TTGCTTTGGA GCTGTTAAAT ACCGCGATAG AGCATGTTGT GGATTTAGTT ACCGATCAGT**

**481 ATCACCCGCT CGCAAAAGCG GCAAAGGACG CGGCCGCCGG AGCCGTTTGC GTCTTTGCCG**

**541 TGATATCGTG TATCATTGGT TTACTCATCT TCTTGCCGAA GATATGTTAG CAGAAGATTC**

**601 TTACAATTAT TTTACATTGC CAAAAATGGG CGTGAAAAAC CAATCATAAT TATGTAAAAT**

**661 AAACTTTTAA GGCAATGTGG CCGCAAAATC TCGATCTTTA TCGTTCAATT TTATTCCGAT**

**721 CAGGCAATAG TTGAACTTTT TCACCGTGGC TCAGCCACGA AAAAAA**TCTA GAGAATTC**TC**

**781 TAGACTCCTT CATCTTTTAA TCTATACAAC GTATGTCATA CTCTTACAAT CCGCATAGGC**

**841 AAACAAAGCC TGATTCGAAA ATTCAGCTGT TTGATTAAAT ATTGAAAAAA ACACAAGCAA**

**901 AACGGCATTC GACTTATACC AATCTCTTCA ATCAGGAATA TGAATAAACG TGAGGTTGAA**

**961 ATTGTTAAAG GAGGTGTATG TCCGCATGAG CGAAGATCGT TTGGAGCTTA AAAAGAACTT**

**1021 ATTGAAAACT CTTTTAGCCC CTGTATTTGA CAACAGCGCT TTATCTGGCG GTACAGATGG**

**1081 AGGAGATAAC GTGAAAAACA GCGTAAAAGA ATTATTGGAA GCGGCAATTG ATGCAAAAGT**

**1141 AGATGAAGCA GCGGTGAAAG ACAGTCTCAG CGCACAATCT GTATTGGGAA GCGGTATTTC**

**1201 ATTAGCGGCG AGATGGATTT TTGCAAGAAT TCAGCCCGGC ACTGTCATCA GCATTGTGAT**

**1261 GGATTCAGGA GACATGATCG GCCCGGTTCA ATTTGTTGCC TTTGACGAAA TTCACGGAAT**

**1321 CGTTTTTGTT ACGCAGGAAA ATTCCGTTAC ACCGGCAGGC TCTGCTACAA CATTGCTTGA**

**1381 CGTCGACAAA GTTGAAAGTG TCACGTTCAC TTCG**GGGGGC GGTGGTGGTA AA**ATGGCTTC**

**1441 TTCTGAAAAC GTAATCACTG AATTCATGCG CTTCAAAGTT CGTATGGAAG GTACTGTAAA**

**1501 CGGTCACGAA TTCGAAATCG AAGGTGAAGG TGAAGGACGT CCATACGAAG GTCACAACAC**

**1561 TGTTAAACTT AAAGTTACTA AAGGTGGTCC ATTACCATTC GCTTGGGATA TCCTTTCTCC**

**1621 ACAATTCCAA TACGGTTCTA AAGTATACGT TAAACACCCA GCAGATATCC CTGACTACAA**

**1681 AAAACTTTCT TTCCCAGAAG GTTTCAAATG GGAACGTGTT ATGAACTTCG AAGATGGTGG**

**1741 TGTAGCTACT GTTACTCAAG ATTCTTCTCT TCAAGATGGT TGTTTCATTT ACAAAGTTAA**

**1801 ATTCATCGGT GTAAACTTCC CTTCTGATGG TCCAGTAATG CAAAAGAAAA CTATGGGTTG**

**1861 GGAAGCTTCT ACTGAACGTT TATACCCACG TGACGGTGTT TTAAAAGGTG AAACTCACAA**

**1921 AGCTCTTAAA CTTAAAGACG GTGGTCACTA CTTAGTTGAA TTCAAATCTA TCTACATGGC**

**1981 TAAAAAACCT GTTCAACTTC CAGGTTACTA CTACGTTGAC GCAAAACTTG ATATCACTTC**

**2041 TCACAACGAA GATTACACTA TCGTTGAACA ATACGAACGT ACTGAAGGTC GTCACCACTT**

**2101 ATTCTTATAA** **CAGCATATGA ACGTTTTATA TATCCGTTCA GGATATAAAG GCATATACAC**

**2161 GTATTTTGAC CGCTGGATCG AGGAAGGCTT TTTACAGTCT TCCGTCCGCT TTTTTTCTGT**

**2221 TTCTGAGTTT GAGGAAGATA CCATCCGCAG TATACGGGCA TTCAAACCAG ACATCAGTCT**

**2281 GATGATGACG GGAGACCGTA TTCCGTCTGA ATGGCTTCAA TGGCTGAAGA CAGAACAGAT**

**2341 TCCCGTTTAT CTATGGATGA CGGAAGATCC TTTTTATTTC GATGTGAGCA TGTCAATCGC**

**2401 CCCTTTCGCC GATGCCGTTT TAACGATAGA ACAAAACGCG CTTGATGCTT ACCGGCAAAT**

**2461 GGGCTTACAG CACGTATATT ATGTCCCGAT CCCCGTTAAT CAGCGGCTTT TTAAGAAACA**

**2521 ACCTGCTGAA GCTTCTCTTC ACACAAATCT GCTTCTGATC GGATACCCGT ACCCGAACCG**

**2581 CGTTGAACTG GTGAAAGCAG CGGCGAAGCT TCCGTATACG CTTCGTATCA TCGGGAAGGG**

**2641 ATGGCGCAGA CATCTGCCTA AAAAAATCGC CAGGCAGCAA AATGTGCTCG TAATGGATGA**

**2701 ATGGATTGCA CCCGAA**CTCG AGTTAAGCCA GCCCCGACAC CCGCCAACAC CCGCTGACGC

2761 GCCCTGACGG GCTTGTCTGC TCCCGGCATC CGCTTACAGA CAAGCTGTGA CCGTCTCCGG

2821 GAGCTGCATG TGTCAGAGGT TTTCACCGTC ATCACCGAAA CGCGCGAGAC GAAAGGGCCT

2881 CGTGATACGC CTATTTTTAT AGGTTAATGT CATGATAATA ATGGTTTCTT AGACGTCAGG

2941 TGGCACTTTT CGGGGAAATG TGCGCGGAAC CCCTATTTGT TTATTTTTCT AAATACATTC

3001 AAATATGTAT CCGCTCATGA GACAATAACC CTGATAAATG CTTCAATAAT AAAAAAGGAT

3061 TGATTCTAAT GAAGAAAGCA GACAAGTAAG CCTCCTAAAT TCACTTTAGA TAAAAATTTA

3121 GGAGGCATAT CAA**ATGAACT TTAATAAAAT TGATTTAGAC AATTGGAAGA GAAAAGAGAT**

**3181 ATTTAATCAT TATTTGAACC AACAAACGAC TTTTAGTATA ACCACAGAAA TTGATATTAG**

**3241 TGTTTTATAC CGAAACATAA AACAAGAAGG ATATAAATTT TACCCTGCAT TTATTTTCTT**

**3301 AGTGACAAGG GTGATAAACT CAAATACAGC TTTTAGAACT GGTTACAATA GCGACGGAGA**

**3361 GTTAGGTTAT TGGGATAAGT TAGAGCCACT TTATACAATT TTTGATGGTG TATCTAAAAC**

**3421 ATTCTCTGGT ATTTGGACTC CTGTAAAGAA TGACTTCAAA GAGTTTTATG ATTTATACCT**

**3481 TTCTGATGTA GAGAAATATA ATGGTTCGGG GAAATTGTTT CCCAAAACAC CTATACCTGA**

**3541 AAATGCTTTT TCTCTTTCTA TTATTCCATG GACTTCATTT ACTGGGTTTA ACTTAAATAT**

**3601 CAATAATAAT AGTAATTACC TTCTACCCAT TATTACAGCA GGAAAATTCA TTAATAAAGG**

**3661 TAATTCAATA TATTTACCGC TATCTTTACA GGTACATCAT TCTGTTTGTG ATGGTTATCA**

**3721 TGCAGGATTG TTTATGAACT CTATTCAGGA ATTGTCAGAT AGGCCTAATG ACTGGCTTTT**

**3781 ATAATATGAG ATAA**TGCCGA CTGTACTTTT TACAGTCGGT TTTCTAATGT CACTAACCTG

3841 CCCCGTTAGT TGAAGAAGGT TTTTATATTA CAGCTCCAGA TCCATATCCT TCTTTTTCTG

3901 AACCGACTTC TCCTTTTTCG CTTCTTTATT CCAATTGCTT TATTGACGTT GAGCCTCGGA

3961 ACCCTTAACA ATCCCAAAAC TTGTCGAATG GTCGGCTTAA TAGCTCACGC TATGCCGACA

4021 TTCGTCTGCA AGTTTAGTTA AGGGTTCTTC TCAACGCACA ATAAATTTTC TCGGCATAAA

4081 TGCGTGGTCT AATTTTTATT TTTAATAACC TTGATAGCAA AAAATGCCAT TCCAATACAA

4141 AACCACATAC CTATAATCGA TAACCACATA ACAGTCATAA AACCACTCCT TTTTAACAAA

4201 CTTTATCACA AGAAATATTT ACCCGGG**GTC CAGAAGGTCG ATAGAAAGCG TGAGAAACAG**

**4261 CGTACAGACG ATTTAGAGAT GTAGAGGTAC TTTTATGCCG AGAAAACTTT TTGCGTGTGA**

**4321 CAGTCCTTAA AATATACTTA GAGCGTAAGC GAAAGTAGTA GCGACAGCTA TTAACTTTCG**

**4381 GTTGCAAAGC TCTAGGATTT TTAATGGACG CAGCGCATCA CACGCAAAAA GGAAATTGGA**

**4441 ATAAATGCGA AATTTGAGAT GTTAATTAAA GACCTTTTTG AGGTCTTTTT TTCTTAGATT**

**4501 TTTGGGGTTA TTTAGGGGAG AAAACATAGG GGGGTACTAC GACCTCCCCC CTAGGTGTCC**

**4561 ATTGTCCATT GTCCAAACAA ATAAATAAAT ATTGGGTTTT TAATGTTAAA AGGTTGTTTT**

**4621 TTATGTTAAA GTGAAAAAAA CAGATGTTGG GAGGTACAGT GATGGTTGTA GATAGAAAAG**

**4681 AAGAGAAAAA AGTTGCTGTT ACTTTAAGAC TTACAACAGA AGAAAATGAG ATATTAAATA**

**4741 GAATCAAAGA AAAATATAAT ATTAGCAAAT CAGATGCAAC CGGTATTCTA ATAAAAAAAT**

**4801 ATGCAAAGGA GGAATACGGT GCATTTTAAA CAAAAAAAGA TAGACAGCAC TGGCATGCTG**

**4861 CCTATCTATG ACTAAATTTT GTTAAGTGTA TTAGCACCGT TATTATATCA TGAGCGAAAA**

**4921 TGTAATAAAA GAAACTGAAA ACAAGAAAAA TTCAAGAGGA CGTAATTGGA CATTTGTTTT**

**4981 ATATCCAGAA TCAGCAAAAG CCGAGTGGTT AGAGTATTTA AAAGAGTTAC ACATTCAATT**

**5041 TGTAGTGTCT CCATTACATG ATAGGGATAC TGATACAGAA GGTAGGATGA AAAAAGAGCA**

**5101 TTATCATATT CTAGTGATGT ATGAGGGTAA TAAATCTTAT GAACAGATAA AAATAATTAA**

**5161 CAGAAGAATT GAATGCGACT ATTCCGCAGA TTGCAGGAAG TGTGAAAGGT CTTGTGAGAT**

**5221 ATATGCTTCA CATGGACGAT CCTAATAAAT TTAAATATCA AAAAGAAGAT ATGATAGTTT**

**5281 ATGGCGGTGT AGATGTTGAT GAATTATTAA AGAAAACAAC AACAGATAGA TATAAATTAA**

**5341 TTAAAGAAAT GATTGAGTTT ATTGATGAAC AAGGAATCGT AGAATTTAAG AGTTTAATGG**

**5401 ATTATGCAAT GAAGTTTAAA TTTGATGATT GGTTCCCGCT TTTATGTGAT AACTCGGCGT**

**5461 ATGTTATTCA AGAATATATA AAATCAAATC GGTATAAATC TGACCGATAG ATTTTGAATT**

**5521 TAGGTGTCAC AAGACACTCT TTTTTCGCAC CAGCGAAAAC TGGTTTAAGC CGACTGCGCA**

**5581 AAAGACATAA TCGATTCACA AAAAATAGGC ACACGAAAAA CAAGTTAAGG GATGCAGTTT**

**5641 ATGCATCCCT TAA**CGGTACC ACTGGCCGTC GTTTTACAAC GTCGTGACTG GGAAAACCCT

5701 GGCGTTACCC AACTTAATCG CCTTGCAGCA CATCCCCCTT TCGCCAGCTG GCGTAATAGC

5761 GAAGAGGCCC GCACCGATCG CCCTTCCCAA CAGTTGCGCA GCCTGAATGG CGAATGGCGC

5821 CTGATGCGGT ATTTTCTCCT TACGCATCTG TGCGGTATTT CACACCGCAT ATGGTGCACT

5881 CTCAGTACAA TCTGCTCTGA TGCCGCATAG TTAAGCCAGC CCCGACACCC GCCAACACCC

5941 GCTGACGCGC CCTGACGGGC TTGTCTGCTC CCGGCATCCG CTTACAGACA AGCTGTGACC

6001 GTCTCCGGGA GCTGCATGTG TCAGAGGTTT TCACCGTCAT CACCGAAACG CGCGAGACGA

6061 AAGGGCCTCG TGATACGCCT ATTTTTATAG GTTAATGTCA TGATAATAAT GGTTTCTTAG

6121 ACGTCAGGTG GCACTTTTCG GGGAAATGTG CGCGGAACCC CTATTTGTTT ATTTTTCTAA

6181 ATACATTCAA ATATGTATCC GCTCATGAGA CAATAACCCT GATAAATGCT TCAATAATAT

6241 TGAAAAAGGA AGAGT **ATGAG TATTCAACAT TTCCGTGTCG CCCTTATTCC CTTTTTTGCG**

**6301 GCATTTTGCC TTCCTGTTTT TGCTCACCCA GAAACGCTGG TGAAAGTAAA AGATGCTGAA**

**6361 GATCAGTTGG GTGCACGAGT GGGTTACATC GAACTGGATC TCAACAGCGG TAAGATCCTT**

**6421 GAGAGTTTTC GCCCCGAAGA ACGTTTTCCA ATGATGAGCA CTTTTAAAGT TCTGCTATGT**

**6481 GGCGCGGTAT TATCCCGTAT TGACGCCGGG CAAGAGCAAC TCGGTCGCCG CATACACTAT**

**6541 TCTCAGAATG ACTTGGTTGA GTACTCACCA GTCACAGAAA AGCATCTTAC GGATGGCATG**

**6601 ACAGTAAGAG AATTATGCAG TGCTGCCATA ACCATGAGTG ATAACACTGC GGCCAACTTA**

**6661 CTTCTGACAA CGATCGGAGG ACCGAAGGAG CTAACCGCTT TTTTGCACAA CATGGGGGAT**

**6721 CATGTAACTC GCCTTGATCG TTGGGAACCG GAGCTGAATG AAGCCATACC AAACGACGAG**

**6781 CGTGACACCA CGATGCCTGT AGCAATGGCA ACAACGTTGC GCAAACTATT AACTGGCGAA**

**6841 CTACTTACTC TAGCTTCCCG GCAACAATTA ATAGACTGGA TGGAGGCGGA TAAAGTTGCA**

**6901 GGACCACTTC TGCGCTCGGC CCTTCCGGCT GGCTGGTTTA TTGCTGATAA ATCTGGAGCC**

**6961 GGTGAGCGTG GGTCTCGCGG TATCATTGCA GCACTGGGGC CAGATGGTAA GCCCTCCCGT**

**7021 ATCGTAGTTA TCTACACGAC GGGGAGTCAG GCAACTATGG ATGAACGAAA TAGACAGATC**

**7081 GCTGAGATAG GTGCCTCACT GATTAAGCAT TGGTAA**CTGT CAGACCAAGT TTACTCATAT

7141 ATACTTTAGA TTGATTTAAA ACTTCATTTT TAATTTAAAA GGATCTAGGT GAAGATCCTT

7201 TTTGATAATC TCATGACCAA AATCCCTTAA CGTGAGTTTT CGTTCCACTG AGCGTCAGAC

7261 CCCGTAGAAA AGATCAAAGG ATCTTCTTGA GATCCTTTTT TTCTGCGCGT AATCTGCTGC

7321 TTGCAAACAA AAAAACCACC GCTACCAGCG GTGGTTTGTT TGCCGGATCA AGAGCTACCA

7381 ACTCTTTTTC CGAAGGTAAC TGGCTTCAGC AGAGCGCAGA TACCAAATAC TGTTCTTCTA

7441 GTGTAGCCGT AGTTAGGCCA CCACTTCAAG AACTCTGTAG CACCGCCTAC ATACCTCGCT

7501 CTGCTAATCC TGTTACCAGT GGCTGCTGCC AGTGGCGATA AGTCGTGTCT TACCGGGTTG

7561 GACTCAAGAC GATAGTTACC GGATAAGGCG CAGCGGTCGG GCTGAACGGG GGGTTCGTGC

7621 ACACAGCCCA GCTTGGAGCG AACGACCTAC ACCGAACTGA GATACCTACA GCGTGAGCTA

7681 TGAGAAAGCG CCACGCTTCC CGAAGGGAGA AAGGCGGACA GGTATCCGGT AAGCGGCAGG

7741 GTCGGAACAG GAGAGCGCAC GAGGGAGCTT CCAGGGGGAA ACGCCTGGTA TCTTTATAGT

7801 CCTGTCGGGT TTCGCCACCT CTGACTTGAG CGTCGATTTT TGTGATGCTC GTCAGGGGGG

7861 CGGAGCCTAT GGAAAAACGC CAGCAACGCG GCCTTTTTAC GGTTCCTGGC CTTTTGCTGG

7921 CCTTTTGCTC ACATGTTCTT TCCTGCGTTA TCCCCTGA**TT CTGTGGATAA CCGTATTACC**

**7981 GCCTTTGAGT GAGCTGATAC CGCTCGCCGC AGCCGAACGA CCGAGCGCAG CGAGTCAGTG**

**8041 AGCGAGGAAG CGGAAGA**GCG CCCAATACGC AAACCGCCTC TCCCCGCGCG TTGGCCGATT

8101 CATTAATGCA GCTGGCACGA CAGGTTTCCC GACTGGAAAG CGGGCAGTGA GCGCAACGCA

8161 ATTAATGTGA GTTAGCTCAC TCATTAGGCA CCCCAGGCTT TACACTTTAT GCTTCCGGCT

8221 CGTATGTTGT GTGGAATTGT GAGCGGATAA CAATTTCACA CAGGAAACAG CTATGACCAT

8281 GATTACGCCA AGCTTGCATG CCCGCGG

**Fig S12** **The sequence of pTC-CgeA-RFP with a nucleotide length of 8307 bp**. The bolded segments marked in gray, black, dark blue, red, green, yellow, orange and purple represent gene sequences of *P43* promoter from Bv916 (CP009611), *sgRNA,* *CgeA target,* *rfp*, *Cl^r^*, thermosensitive replication origin for *Bacillus*, *Amp^r^* and replication origin for *Escherichia coli*, respectively.

1 GGATCC**CGTA GGCGCCGACA TGCCGCCTGT TTTAGGAGAT ATTATCATCA GCGCTGACAG**

**61 AACGAAAGAG CAGGCGGAAG AGTACGGCCA TTCATTTATG AGAGAGCTCG GTTTTCTGGC**

**121 CGTGCACGGC TTTTTGCATT TGCTTGGCTA TGATCACATG ACAAAAGAAG AAGAGGAAGA**

**181 AATGTTTTCA AAGCAAAAGG ATTTGCTGGA TGAGTATGGA CTCACGAGAT CATAAAAACG**

**241 AGTGGAGCAG GTTCCTGAAG AGCTTTGTCC ATGCATGGAG GGGAATCTGG AAAACGGCGC**

**301 GGTCAGAGCG GAATTTTCAA TTTCATATTA CCGCGGCCTG CGCCGTTATC GTTTGCGGTT**

**361 TTCTCACCGG GCTGAGCACA GCCGAATGGG CCGTTGTTTT GATGCTGATC GGAGGAATGC**

**421 TTGCTTTGGA GCTGTTAAAT ACCGCGATAG AGCATGTTGT GGATTTAGTT ACCGATCAGT**

**481 ATCACCCGCT CGCAAAAGCG GCAAAGGACG CGGCCGCCGG AGCCGTTTGC GTCTTTGCCG**

**541 TGATATCGTG TATCATTGGT TTACTCATCT TCTTGCCGAA GATATGTTAG CAGAAGATTC**

**601 TTACAATTAT TTTACATTGC CAAAAATGGG CGTGAAAAAC CAATCATAAT TATGTAAAAT**

**661 AAACTAACTT AGCCCTAGCA AAACAAAATC TCGATCTTTA TCGTTCAATT TTATTCCGAT**

**721 CAGGCAATAG TTGAACTTTT TCACCGTGGC TCAGCCACGA AAAAAA**TCTA GAGAATTCAC

781 TAGT**GCCGAA TGGATGAGAA AACGTTTAAT TGCGAAAAGG AATTGACGCT TGCCGTGATA**

**841 GGCGGGAAGT GGAAAATGCT GATTATGTGG CACTTGGGAA AAGAAGGGAC GAAGCGGTTT**

**901 AATGAGCTGA AAGCTTTAAT TCCGGATATT ACGCACAAAA TTCTTGTGAA TCAGCTCAGG**

**961 GAGCTGGAGC AGGATCTGAT CGTTCACAGG GAAGTTTACC CTGTCGTCCC TCCGAAAGTG**

**1021 GAGTATTCTT TAACGGCGCA AGGAGAAAGC CTTATGCCGA TTCTGGACGC CATGTATAAG**

**1081 TGGGGAAAAG ATTATATGGA ATTAATCAAC ATTGATAAAA CTGCAATAAA GGAATCTTTT**

**1141 TGAAGTGCTC TATGTAAAAT AGAGTGCTTT TTTTGCGGTT TAATGAAATC ATATTGCGAA**

**1201 CATTCGGACC GTCTGTTTCG GTTCTTGCGC TGTTTTTTCC GGGTCAGCTT ACGTACGTAA**

**1261 ATTTTTTTCA TTCTGCCATA ACTGGATATT CGACAGATTT TCATTATAGT TTAAAGATTT**

**1321 TTAATTTCGC TTCAGCCCAA CTGCTATTGT TGCAGCCATA ACAGCACAAA TACAGAAAGC**

**1381 GAAATTACCA CCCACGTCGA AATCGATGCA AAACGCTACG GTTTTGAAAG AGATTCCGTT**

**1441 ATTTTGCTGG AGCAAATTCG GACGATTGAC AAGCAAAGGT TAACGGATAA GATTACTCAT**

**1501 CTGGATGATG AAATGATGGA TAAGGTTGAT GAAGCCTTAC AAATCAGTTT GGCACTCATT**

**1561 GATTTTTAGA CATATTTGCA GGTTGCTCAA ATAGAGCAAC TTTTTTTGTT TTCAAAAAAC**

**1621 ATAAACGATA TAATAGTGAA ATAACGAAAA AATATGTTGT TTTTTATTGG GAGGTAAGCG**

**1681 AATTTGGAAA TAACTTTTTA TCCTTTAACA AATGCTCAAA AACGTATCTG GTATACAGAA**

**1741 AAATTCTATC CGAACACAAG TATTTCAAAT CTTGCCGGGT TCGGGAAACT CATTTCTGAA**

**1801 GACGGCGTAC AGGCTCATTA CGTTGAGAAA GCGATACAGG AATTCGTCCG GCGGTATGAG**

**1861 TCGATGAGAA TACGTCTGCG GCTTGATGAT GAGGGGGAGC CCGTTCAATA TGTGAGCGAA**

**1921 TACCGTCCGC TCTCTATCGG GCACACAGAT ATCAGGCAAG CCGGCTGCTC TGCGGACGAG**

**1981 CTGTCAA**AGA TCTCTCGAGT TAAGCCAGCC CCGACACCCG CCAACACCCG CTGACGCGCC

2041 CTGACGGGCT TGTCTGCTCC CGGCATCCGC TTACAGACAA GCTGTGACCG TCTCCGGGAG

2101 CTGCATGTGT CAGAGGTTTT CACCGTCATC ACCGAAACGC GCGAGACGAA AGGGCCTCGT

2161 GATACGCCTA TTTTTATAGG TTAATGTCAT GATAATAATG GTTTCTTAGA CGTCAGGTGG

2221 CACTTTTCGG GGAAATGTGC GCGGAACCCC TATTTGTTTA TTTTTCTAAA TACATTCAAA

2281 TATGTATCCG CTCATGAGAC AATAACCCTG ATAAATGCTT CAATAATAAA AAAGGATTGA

2341 TTCTAATGAA GAAAGCAGAC AAGTAAGCCT CCTAAATTCA CTTTAGATAA AAATTTAGGA

2401 GGCATATCAA **ATGAACTTTA ATAAAATTGA TTTAGACAAT TGGAAGAGAA AAGAGATATT**

**2461 TAATCATTAT TTGAACCAAC AAACGACTTT TAGTATAACC ACAGAAATTG ATATTAGTGT**

**2521 TTTATACCGA AACATAAAAC AAGAAGGATA TAAATTTTAC CCTGCATTTA TTTTCTTAGT**

**2581 GACAAGGGTG ATAAACTCAA ATACAGCTTT TAGAACTGGT TACAATAGCG ACGGAGAGTT**

**2641 AGGTTATTGG GATAAGTTAG AGCCACTTTA TACAATTTTT GATGGTGTAT CTAAAACATT**

**2701 CTCTGGTATT TGGACTCCTG TAAAGAATGA CTTCAAAGAG TTTTATGATT TATACCTTTC**

**2761 TGATGTAGAG AAATATAATG GTTCGGGGAA ATTGTTTCCC AAAACACCTA TACCTGAAAA**

**2821 TGCTTTTTCT CTTTCTATTA TTCCATGGAC TTCATTTACT GGGTTTAACT TAAATATCAA**

**2881 TAATAATAGT AATTACCTTC TACCCATTAT TACAGCAGGA AAATTCATTA ATAAAGGTAA**

**2941 TTCAATATAT TTACCGCTAT CTTTACAGGT ACATCATTCT GTTTGTGATG GTTATCATGC**

**3001 AGGATTGTTT ATGAACTCTA TTCAGGAATT GTCAGATAGG CCTAATGACT GGCTTTTATA**

**3061 ATATGAGATA A**TGCCGACTG TACTTTTTAC AGTCGGTTTT CTAATGTCAC TAACCTGCCC

3121 CGTTAGTTGA AGAAGGTTTT TATATTACAG CTCCAGATCC ATATCCTTCT TTTTCTGAAC

3181 CGACTTCTCC TTTTTCGCTT CTTTATTCCA ATTGCTTTAT TGACGTTGAG CCTCGGAACC

3241 CTTAACAATC CCAAAACTTG TCGAATGGTC GGCTTAATAG CTCACGCTAT GCCGACATTC

3301 GTCTGCAAGT TTAGTTAAGG GTTCTTCTCA ACGCACAATA AATTTTCTCG GCATAAATGC

3361 GTGGTCTAAT TTTTATTTTT AATAACCTTG ATAGCAAAAA ATGCCATTCC AATACAAAAC

3421 CACATACCTA TAATCGATAA CCACATAACA GTCATAAAAC CACTCCTTTT TAACAAACTT

3481 TATCACAAGA AATATTTACC CGGG**GTCCAG AAGGTCGATA GAAAGCGTGA GAAACAGCGT**

**3541 ACAGACGATT TAGAGATGTA GAGGTACTTT TATGCCGAGA AAACTTTTTG CGTGTGACAG**

**3601 TCCTTAAAAT ATACTTAGAG CGTAAGCGAA AGTAGTAGCG ACAGCTATTA ACTTTCGGTT**

**3661 GCAAAGCTCT AGGATTTTTA ATGGACGCAG CGCATCACAC GCAAAAAGGA AATTGGAATA**

**3721 AATGCGAAAT TTGAGATGTT AATTAAAGAC CTTTTTGAGG TCTTTTTTTC TTAGATTTTT**

**3781 GGGGTTATTT AGGGGAGAAA ACATAGGGGG GTACTACGAC CTCCCCCCTA GGTGTCCATT**

**3841 GTCCATTGTC CAAACAAATA AATAAATATT GGGTTTTTAA TGTTAAAAGG TTGTTTTTTA**

**3901 TGTTAAAGTG AAAAAAACAG ATGTTGGGAG GTACAGTGAT GGTTGTAGAT AGAAAAGAAG**

**3961 AGAAAAAAGT TGCTGTTACT TTAAGACTTA CAACAGAAGA AAATGAGATA TTAAATAGAA**

**4021 TCAAAGAAAA ATATAATATT AGCAAATCAG ATGCAACCGG TATTCTAATA AAAAAATATG**

**4081 CAAAGGAGGA ATACGGTGCA TTTTAAACAA AAAAAGATAG ACAGCACTGG CATGCTGCCT**

**4141 ATCTATGACT AAATTTTGTT AAGTGTATTA GCACCGTTAT TATATCATGA GCGAAAATGT**

**4201 AATAAAAGAA ACTGAAAACA AGAAAAATTC AAGAGGACGT AATTGGACAT TTGTTTTATA**

**4261 TCCAGAATCA GCAAAAGCCG AGTGGTTAGA GTATTTAAAA GAGTTACACA TTCAATTTGT**

**4321 AGTGTCTCCA TTACATGATA GGGATACTGA TACAGAAGGT AGGATGAAAA AAGAGCATTA**

**4381 TCATATTCTA GTGATGTATG AGGGTAATAA ATCTTATGAA CAGATAAAAA TAATTAACAG**

**4441 AAGAATTGAA TGCGACTATT CCGCAGATTG CAGGAAGTGT GAAAGGTCTT GTGAGATATA**

**4501 TGCTTCACAT GGACGATCCT AATAAATTTA AATATCAAAA AGAAGATATG ATAGTTTATG**

**4561 GCGGTGTAGA TGTTGATGAA TTATTAAAGA AAACAACAAC AGATAGATAT AAATTAATTA**

**4621 AAGAAATGAT TGAGTTTATT GATGAACAAG GAATCGTAGA ATTTAAGAGT TTAATGGATT**

**4681 ATGCAATGAA GTTTAAATTT GATGATTGGT TCCCGCTTTT ATGTGATAAC TCGGCGTATG**

**4741 TTATTCAAGA ATATATAAAA TCAAATCGGT ATAAATCTGA CCGATAGATT TTGAATTTAG**

**4801 GTGTCACAAG ACACTCTTTT TTCGCACCAG CGAAAACTGG TTTAAGCCGA CTGCGCAAAA**

**4861 GACATAATCG ATTCACAAAA AATAGGCACA CGAAAAACAA GTTAAGGGAT GCAGTTTATG**

**4921 CATCCCTTAA C**GGTACCACT GGCCGTCGTT TTACAACGTC GTGACTGGGA AAACCCTGGC

4981 GTTACCCAAC TTAATCGCCT TGCAGCACAT CCCCCTTTCG CCAGCTGGCG TAATAGCGAA

5041 GAGGCCCGCA CCGATCGCCC TTCCCAACAG TTGCGCAGCC TGAATGGCGA ATGGCGCCTG

5101 ATGCGGTATT TTCTCCTTAC GCATCTGTGC GGTATTTCAC ACCGCATATG GTGCACTCTC

5161 AGTACAATCT GCTCTGATGC CGCATAGTTA AGCCAGCCCC GACACCCGCC AACACCCGCT

5221 GACGCGCCCT GACGGGCTTG TCTGCTCCCG GCATCCGCTT ACAGACAAGC TGTGACCGTC

5281 TCCGGGAGCT GCATGTGTCA GAGGTTTTCA CCGTCATCAC CGAAACGCGC GAGACGAAAG

5341 GGCCTCGTGA TACGCCTATT TTTATAGGTT AATGTCATGA TAATAATGGT TTCTTAGACG

5401 TCAGGTGGCA CTTTTCGGGG AAATGTGCGC GGAACCCCTA TTTGTTTATT TTTCTAAATA

5461 CATTCAAATA TGTATCCGCT CATGAGACAA TAACCCTGAT AAATGCTTCA ATAATATTGA

5521 AAAAGGAAGA GT**ATGAGTAT TCAACATTTC CGTGTCGCCC TTATTCCCTT TTTTGCGGCA**

**5581 TTTTGCCTTC CTGTTTTTGC TCACCCAGAA ACGCTGGTGA AAGTAAAAGA TGCTGAAGAT**

**5641 CAGTTGGGTG CACGAGTGGG TTACATCGAA CTGGATCTCA ACAGCGGTAA GATCCTTGAG**

**5701 AGTTTTCGCC CCGAAGAACG TTTTCCAATG ATGAGCACTT TTAAAGTTCT GCTATGTGGC**

**5761 GCGGTATTAT CCCGTATTGA CGCCGGGCAA GAGCAACTCG GTCGCCGCAT ACACTATTCT**

**5821 CAGAATGACT TGGTTGAGTA CTCACCAGTC ACAGAAAAGC ATCTTACGGA TGGCATGACA**

**5881 GTAAGAGAAT TATGCAGTGC TGCCATAACC ATGAGTGATA ACACTGCGGC CAACTTACTT**

**5941 CTGACAACGA TCGGAGGACC GAAGGAGCTA ACCGCTTTTT TGCACAACAT GGGGGATCAT**

**6001 GTAACTCGCC TTGATCGTTG GGAACCGGAG CTGAATGAAG CCATACCAAA CGACGAGCGT**

**6061 GACACCACGA TGCCTGTAGC AATGGCAACA ACGTTGCGCA AACTATTAAC TGGCGAACTA**

**6121 CTTACTCTAG CTTCCCGGCA ACAATTAATA GACTGGATGG AGGCGGATAA AGTTGCAGGA**

**6181 CCACTTCTGC GCTCGGCCCT TCCGGCTGGC TGGTTTATTG CTGATAAATC TGGAGCCGGT**

**6241 GAGCGTGGGT CTCGCGGTAT CATTGCAGCA CTGGGGCCAG ATGGTAAGCC CTCCCGTATC**

**6301 GTAGTTATCT ACACGACGGG GAGTCAGGCA ACTATGGATG AACGAAATAG ACAGATCGCT**

**6361 GAGATAGGTG CCTCACTGAT TAAGCATTGG TAA**CTGTCAG ACCAAGTTTA CTCATATATA

6421 CTTTAGATTG ATTTAAAACT TCATTTTTAA TTTAAAAGGA TCTAGGTGAA GATCCTTTTT

6481 GATAATCTCA TGACCAAAAT CCCTTAACGT GAGTTTTCGT TCCACTGAGC GTCAGACCCC

6541 GTAGAAAAGA TCAAAGGATC TTCTTGAGAT CCTTTTTTTC TGCGCGTAAT CTGCTGCTTG

6601 CAAACAAAAA AACCACCGCT ACCAGCGGTG GTTTGTTTGC CGGATCAAGA GCTACCAACT

6661 CTTTTTCCGA AGGTAACTGG CTTCAGCAGA GCGCAGATAC CAAATACTGT TCTTCTAGTG

6721 TAGCCGTAGT TAGGCCACCA CTTCAAGAAC TCTGTAGCAC CGCCTACATA CCTCGCTCTG

6781 CTAATCCTGT TACCAGTGGC TGCTGCCAGT GGCGATAAGT CGTGTCTTAC CGGGTTGGAC

6841 TCAAGACGAT AGTTACCGGA TAAGGCGCAG CGGTCGGGCT GAACGGGGGG TTCGTGCACA

6901 CAGCCCAGCT TGGAGCGAAC GACCTACACC GAACTGAGAT ACCTACAGCG TGAGCTATGA

6961 GAAAGCGCCA CGCTTCCCGA AGGGAGAAAG GCGGACAGGT ATCCGGTAAG CGGCAGGGTC

7021 GGAACAGGAG AGCGCACGAG GGAGCTTCCA GGGGGAAACG CCTGGTATCT TTATAGTCCT

7081 GTCGGGTTTC GCCACCTCTG ACTTGAGCGT CGATTTTTGT GATGCTCGTC AGGGGGGCGG

7141 AGCCTATGGA AAAACGCCAG CAACGCGGCC TTTTTACGGT TCCTGGCCTT TTGCTGGCCT

7201 TTTGCTCACA TGTTCTTTCC TGCGTTATCC CCTGA**TTCTG TGGATAACCG TATTACCGCC**

**7261 TTTGAGTGAG CTGATACCGC TCGCCGCAGC CGAACGACCG AGCGCAGCGA GTCAGTGAGC**

**7321 GAGGAAGCGG AAGA**GCGCCC AATACGCAAA CCGCCTCTCC CCGCGCGTTG GCCGATTCAT

7381 TAATGCAGCT GGCACGACAG GTTTCCCGAC TGGAAAGCGG GCAGTGAGCG CAACGCAATT

7441 AATGTGAGTT AGCTCACTCA TTAGGCACCC CAGGCTTTAC ACTTTATGCT TCCGGCTCGT

7501 ATGTTGTGTG GAATTGTGAG CGGATAACAA TTTCACACAG GAAACAGCTA TGACCATGAT

7561 TACGCCAAGC TTGCATGCCC GCGG

**Fig S13** **The sequence of pTC-PA-srf with a nucleotide length of 7584 bp**. The bolded segments marked in gray, black, dark blue, red, green, yellow, orange and purple represent gene sequences of *P43* promoter from Bv916 (CP009611), *sgRNA,* *srf target,* *PA* promoter from *Bacillus subtilis* regulatory gene (*rsbU*), *Cl^r^*, thermosensitive replication origin for *Bacillus*, *Amp^r^* and replication origin for *Escherichia coli*, respectively.

1 GGATCC**CGTA GGCGCCGACA TGCCGCCTGT TTTAGGAGAT ATTATCATCA GCGCTGACAG**

**61 AACGAAAGAG CAGGCGGAAG AGTACGGCCA TTCATTTATG AGAGAGCTCG GTTTTCTGGC**

**121 CGTGCACGGC TTTTTGCATT TGCTTGGCTA TGATCACATG ACAAAAGAAG AAGAGGAAGA**

**181 AATGTTTTCA AAGCAAAAGG ATTTGCTGGA TGAGTATGGA CTCACGAGAT CATAAAAACG**

**241 AGTGGAGCAG GTTCCTGAAG AGCTTTGTCC ATGCATGGAG GGGAATCTGG AAAACGGCGC**

**301 GGTCAGAGCG GAATTTTCAA TTTCATATTA CCGCGGCCTG CGCCGTTATC GTTTGCGGTT**

**361 TTCTCACCGG GCTGAGCACA GCCGAATGGG CCGTTGTTTT GATGCTGATC GGAGGAATGC**

**421 TTGCTTTGGA GCTGTTAAAT ACCGCGATAG AGCATGTTGT GGATTTAGTT ACCGATCAGT**

**481 ATCACCCGCT CGCAAAAGCG GCAAAGGACG CGGCCGCCGG AGCCGTTTGC GTCTTTGCCG**

**541 TGATATCGTG TATCATTGGT TTACTCATCT TCTTGCCGAA GATATGTTAG CAGAAGATTC**

**601 TTACAATTAT TTTACATTGC CAAAAATGGG CGTGAAAAAC CAATCATAAT TATGTAAAAT**

**661 AAACGTATGT CTCAGATTAT ATGCAAAATC TCGATCTTTA TCGTTCAATT TTATTCCGAT**

**721 CAGGCAATAG TTGAACTTTT TCACCGTGGC TCAGCCACGA AAAAAA**TCTA GAGAATTCAC

781 TAGT**GAGCTT GACGAAGAAA TCGTTCTAGC AAAAAGGAAT CATGGATCTT ATTGGAGTGT**

**841 GCTTGTATCG GATCAACAGA AATTGCCGAA TGGTGTAACG GCCAACACCA TTTCCATAGA**

**901 AGAAATCAGC GTCTTTTTAA CGAGGAGTGA AGACATTGAG AGGTCTGTTA CTGACTAACT**

**961 ATTACTTGGT TTATCGAAGT TTCTTTGCAT ACACGGGGTT GGCCATATTG GTATCAGCAG**

**1021 TCATTTTTTA TAGGGCATAA CGTGCCGATT TGTTTTTCTA AATGGTATTT AGGGTCATCT**

**1081 TTCATCAGAT TCTTCTTCCA ACGATAATAA GATGCTCGTG AAATACCTAA ATGAATACAG**

**1141 ATATCCTGTA TGGTCATTGT GCCGCACAAT GTTTCTACAA GCTCGCTGGC GTTTCGCTAT**

**1201 CAGCTTCCTT TCCAATTCGT TGTACTTTTT TAACACTTCA TTCTGTTGTC TCAGATAACG**

**1261 ATTTTCTGCC TGCAGTTTCT CTAATTAGGA AGAATACTTT GGACCTTTTC CATAAGTATA**

**1321 TTGCTTTCCG ACAGGTTGTT CGAATCGGTT ATTACGATGT TTTATGCGAA TTACAATATG**

**1381 GACAAGCATC AATTTACATA TGCTTCAGCG GGACATGAAC CGGGATTCTA TTATTCTCAA**

**1441 AAAGACAACA CGTTTTACGA TTTAGAGGCC AAAGGACTCG TTCTCGGCAT CTCGCAGGAC**

**1501 TATGACTACA AACAATTCGA TCAGCATCTG GAAAAGGGCG ATATGATTGT TTTATTTTCT**

**1561 GACGGCGTCA CAGAATGCAG AACGGAAAAC GGTTTCTTGG AGCGTCCTGA TCTGCAGAAG**

**1621 CTCATTGAGG AACATATGTG TTCCTCTGCG CAGGAAATGG TCAAAAACAT TTATGACAGC**

**1681 CTCCTCAAAT TGCAGGATTT TCAGCTTCAC GATGATTTTA CGTTAATTGT TTTGCGGAGA**

**1741 AAGGTTTAAC GTCTGTCAGA CGAGGGTATA AAGCAACTAG TGATTTGAAG GAAAATTTGA**

**1801 GGTGATACGA** **ATATCAATCG TCATTTCATT CGGGGGGGAT AAAACAATGA CTTTATTAAG**

**1861 TGCTATTCAA AAAAAAGCAA GTGAAGAAAC CAAAGGAATT ACTTTTGTTA AAAATAGAAA**

**1921 AAAAGAAAAG CGTGTATCAT ATAAAGAATT GTTGATGACG AGTTTGAGGA TATTAGGTTT**

**1981 TTTACAAAGT AAAGGAGTTA CTTATAAGGA TGAGATAGTT TTTCAATTAA AAGATAATGA**

**2041 GGACTTCATC AATGTATTCT GGGCGGGAAT ACTTGGTGGT** AGATCTCTCG AGTAACAGGT

2101 TGGCTGATAA GTCCCCGGTC TGCCACATAG ATGGCGTCGC TAGTATTAAA TGCATATTAT

2161 TTTTATATAG TACCAACCTT CAAATGATTC CCTATAGCTT GTAAATTCTA TCATAATTGT

2221 GGTTTCAAAA TCGGCTCCGT CGATACTATG TTATACGCCA ACTTTGAAAA CAACTTTGAA

2281 AAAGCTGTTT TCTGGTATTT AAGGTTTTAG AATGCAAGGA ACAGTGAATT GGAGTTCGTC

2341 TTGTTATAAT TAGCTTCTTG GGGTATCTTT AAATACTGTA GAAAAGAGGA AGGAAATAAT

2401 AA **ATGGCTAA AATGAGAATA TCACCGGAAT TGAAAAAACT GATCGAAAAA TACCGCTGCG**

**2461 TAAAAGATAC GGAAGGAATG TCTCCTGCTA AGGTATATAA GCTGGTGGGA GAAAATGAAA**

**2521 ACCTATATTT AAAAATGACG GACAGCCGGT ATAAAGGGAC CACCTATGAT GTAGAACGGG**

**2581 AAAAGGACAT GATGCTATGG CTGGAAGGAA AGCTGCCTGT TCCAAAGGTC CTGCACTTTG**

**2641 AACGGCATGA TGGCTGGAGC AATCTGCTCA TGAGTGAGGC CGATGGCGTC CTTTGCTCGG**

**2701 AAGAGTATGA AGATGAACAA AGCCCTGAAA AGATTATCGA GCTGTATGCG GAGTGCATCA**

**2761 GGCTCTTTCA CTCCATCGAC ATATCGGATT GTCCCTATAC GAATAGCTTA GACAGCCGCT**

**2821 TAGCCGAATT GGATTACTTA CTGAATAACG ATCTGGCCGA TGTGGATTGC GAAAACTGGG**

**2881 AAGAAGACAC TCCATTTAAA GATCCGCGCG AGCTGTATGA TTTTTTAAAG ACGGAAAAGC**

**2941 CCGAAGAGGA ACTTGTCTTT TCCCACGGCG ACCTGGGAGA CAGCAACATC TTTGTGAAAG**

**3001 ATGGCAAAGT AAGTGGCTTT ATTGATCTTG GGAGAAGCGG CAGGGCGGAC AAGTGGTATG**

**3061 ACATTGCCTT CTGCGTCCGG TCGATCAGGG AGGATATCGG GGAAGAACAG TATGTCGAGC**

**3121 TATTTTTTGA CTTACTGGGG ATCAAGCCTG ATTGGGAGAA AATAAAATAT TATATTTTAC**

**3181 TGGATGAATT GTTTTAG**TAC CTAGAATTTA GATGTCTAAA AAGCTTTAAC TACAAGCTTT

3241 TTAGACATCT AATCTTTTCT GAAGTACATC CGCAACTGTC CATACTCTGA TGTTTTATAT

3301 CTTTTCTAAA AGTTCGCTAG ATAGGGGTCC CGAGCGCCTA CGAGGAATTT GTATCGCCCG

3361 GG**GTCCAGAA GGTCGATAGA AAGCGTGAGA AACAGCGTAC AGACGATTTA GAGATGTAGA**

**3421 GGTACTTTTA TGCCGAGAAA ACTTTTTGCG TGTGACAGTC CTTAAAATAT ACTTAGAGCG**

**3481 TAAGCGAAAG TAGTAGCGAC AGCTATTAAC TTTCGGTTGC AAAGCTCTAG GATTTTTAAT**

**3541 GGACGCAGCG CATCACACGC AAAAAGGAAA TTGGAATAAA TGCGAAATTT GAGATGTTAA**

**3601 TTAAAGACCT TTTTGAGGTC TTTTTTTCTT AGATTTTTGG GGTTATTTAG GGGAGAAAAC**

**3661 ATAGGGGGGT ACTACGACCT CCCCCCTAGG TGTCCATTGT CCATTGTCCA AACAAATAAA**

**3721 TAAATATTGG GTTTTTAATG TTAAAAGGTT GTTTTTTATG TTAAAGTGAA AAAAACAGAT**

**3781 GTTGGGAGGT ACAGTGATGG TTGTAGATAG AAAAGAAGAG AAAAAAGTTG CTGTTACTTT**

**3841 AAGACTTACA ACAGAAGAAA ATGAGATATT AAATAGAATC AAAGAAAAAT ATAATATTAG**

**3901 CAAATCAGAT GCAACCGGTA TTCTAATAAA AAAATATGCA AAGGAGGAAT ACGGTGCATT**

**3961 TTAAACAAAA AAAGATAGAC AGCACTGGCA TGCTGCCTAT CTATGACTAA ATTTTGTTAA**

**4021 GTGTATTAGC ACCGTTATTA TATCATGAGC GAAAATGTAA TAAAAGAAAC TGAAAACAAG**

**4081 AAAAATTCAA GAGGACGTAA TTGGACATTT GTTTTATATC CAGAATCAGC AAAAGCCGAG**

**4141 TGGTTAGAGT ATTTAAAAGA GTTACACATT CAATTTGTAG TGTCTCCATT ACATGATAGG**

**4201 GATACTGATA CAGAAGGTAG GATGAAAAAA GAGCATTATC ATATTCTAGT GATGTATGAG**

**4261 GGTAATAAAT CTTATGAACA GATAAAAATA ATTAACAGAA GAATTGAATG CGACTATTCC**

**4321 GCAGATTGCA GGAAGTGTGA AAGGTCTTGT GAGATATATG CTTCACATGG ACGATCCTAA**

**4381 TAAATTTAAA TATCAAAAAG AAGATATGAT AGTTTATGGC GGTGTAGATG TTGATGAATT**

**4441 ATTAAAGAAA ACAACAACAG ATAGATATAA ATTAATTAAA GAAATGATTG AGTTTATTGA**

**4501 TGAACAAGGA ATCGTAGAAT TTAAGAGTTT AATGGATTAT GCAATGAAGT TTAAATTTGA**

**4561 TGATTGGTTC CCGCTTTTAT GTGATAACTC GGCGTATGTT ATTCAAGAAT ATATAAAATC**

**4621 AAATCGGTAT AAATCTGACC GATAGATTTT GAATTTAGGT GTCACAAGAC ACTCTTTTTT**

**4681 CGCACCAGCG AAAACTGGTT TAAGCCGACT GCGCAAAAGA CATAATCGAT TCACAAAAAA**

**4741 TAGGCACACG AAAAACAAGT TAAGGGATGC AGTTTATGCA TCCCTTAAC**G GTACCACTGG

4801 CCGTCGTTTT ACAACGTCGT GACTGGGAAA ACCCTGGCGT TACCCAACTT AATCGCCTTG

4861 CAGCACATCC CCCTTTCGCC AGCTGGCGTA ATAGCGAAGA GGCCCGCACC GATCGCCCTT

4921 CCCAACAGTT GCGCAGCCTG AATGGCGAAT GGCGCCTGAT GCGGTATTTT CTCCTTACGC

4981 ATCTGTGCGG TATTTCACAC CGCATATGGT GCACTCTCAG TACAATCTGC TCTGATGCCG

5041 CATAGTTAAG CCAGCCCCGA CACCCGCCAA CACCCGCTGA CGCGCCCTGA CGGGCTTGTC

5101 TGCTCCCGGC ATCCGCTTAC AGACAAGCTG TGACCGTCTC CGGGAGCTGC ATGTGTCAGA

5161 GGTTTTCACC GTCATCACCG AAACGCGCGA GACGAAAGGG CCTCGTGATA CGCCTATTTT

5221 TATAGGTTAA TGTCATGATA ATAATGGTTT CTTAGACGTC AGGTGGCACT TTTCGGGGAA

5281 ATGTGCGCGG AACCCCTATT TGTTTATTTT TCTAAATACA TTCAAATATG TATCCGCTCA

5341 TGAGACAATA ACCCTGATAA ATGCTTCAAT AATATTGAAA AAGGAAGAGT **ATGAGTATTC**

**5401 AACATTTCCG TGTCGCCCTT ATTCCCTTTT TTGCGGCATT TTGCCTTCCT GTTTTTGCTC**

**5461 ACCCAGAAAC GCTGGTGAAA GTAAAAGATG CTGAAGATCA GTTGGGTGCA CGAGTGGGTT**

**5521 ACATCGAACT GGATCTCAAC AGCGGTAAGA TCCTTGAGAG TTTTCGCCCC GAAGAACGTT**

**5581 TTCCAATGAT GAGCACTTTT AAAGTTCTGC TATGTGGCGC GGTATTATCC CGTATTGACG**

**5641 CCGGGCAAGA GCAACTCGGT CGCCGCATAC ACTATTCTCA GAATGACTTG GTTGAGTACT**

**5701 CACCAGTCAC AGAAAAGCAT CTTACGGATG GCATGACAGT AAGAGAATTA TGCAGTGCTG**

**5761 CCATAACCAT GAGTGATAAC ACTGCGGCCA ACTTACTTCT GACAACGATC GGAGGACCGA**

**5821 AGGAGCTAAC CGCTTTTTTG CACAACATGG GGGATCATGT AACTCGCCTT GATCGTTGGG**

**5881 AACCGGAGCT GAATGAAGCC ATACCAAACG ACGAGCGTGA CACCACGATG CCTGTAGCAA**

**5941 TGGCAACAAC GTTGCGCAAA CTATTAACTG GCGAACTACT TACTCTAGCT TCCCGGCAAC**

**6001 AATTAATAGA CTGGATGGAG GCGGATAAAG TTGCAGGACC ACTTCTGCGC TCGGCCCTTC**

**6061 CGGCTGGCTG GTTTATTGCT GATAAATCTG GAGCCGGTGA GCGTGGGTCT CGCGGTATCA**

**6121 TTGCAGCACT GGGGCCAGAT GGTAAGCCCT CCCGTATCGT AGTTATCTAC ACGACGGGGA**

**6181 GTCAGGCAAC TATGGATGAA CGAAATAGAC AGATCGCTGA GATAGGTGCC TCACTGATTA**

**6241 AGCATTGGTA A**CTGTCAGAC CAAGTTTACT CATATATACT TTAGATTGAT TTAAAACTTC

6301 ATTTTTAATT TAAAAGGATC TAGGTGAAGA TCCTTTTTGA TAATCTCATG ACCAAAATCC

6361 CTTAACGTGA GTTTTCGTTC CACTGAGCGT CAGACCCCGT AGAAAAGATC AAAGGATCTT

6421 CTTGAGATCC TTTTTTTCTG CGCGTAATCT GCTGCTTGCA AACAAAAAAA CCACCGCTAC

6481 CAGCGGTGGT TTGTTTGCCG GATCAAGAGC TACCAACTCT TTTTCCGAAG GTAACTGGCT

6541 TCAGCAGAGC GCAGATACCA AATACTGTTC TTCTAGTGTA GCCGTAGTTA GGCCACCACT

6601 TCAAGAACTC TGTAGCACCG CCTACATACC TCGCTCTGCT AATCCTGTTA CCAGTGGCTG

6661 CTGCCAGTGG CGATAAGTCG TGTCTTACCG GGTTGGACTC AAGACGATAG TTACCGGATA

6721 AGGCGCAGCG GTCGGGCTGA ACGGGGGGTT CGTGCACACA GCCCAGCTTG GAGCGAACGA

6781 CCTACACCGA ACTGAGATAC CTACAGCGTG AGCTATGAGA AAGCGCCACG CTTCCCGAAG

6841 GGAGAAAGGC GGACAGGTAT CCGGTAAGCG GCAGGGTCGG AACAGGAGAG CGCACGAGGG

6901 AGCTTCCAGG GGGAAACGCC TGGTATCTTT ATAGTCCTGT CGGGTTTCGC CACCTCTGAC

6961 TTGAGCGTCG ATTTTTGTGA TGCTCGTCAG GGGGGCGGAG CCTATGGAAA AACGCCAGCA

7021 ACGCGGCCTT TTTACGGTTC CTGGCCTTTT GCTGGCCTTT TGCTCACATG TTCTTTCCTG

7081 CGTTATCCCC TGA**TTCTGTG GATAACCGTA TTACCGCCTT TGAGTGAGCT GATACCGCTC**

**7141 GCCGCAGCCG AACGACCGAG CGCAGCGAGT CAGTGAGCGA GGAAGCGGAA GA**GCGCCCAA

7201 TACGCAAACC GCCTCTCCCC GCGCGTTGGC CGATTCATTA ATGCAGCTGG CACGACAGGT

7261 TTCCCGACTG GAAAGCGGGC AGTGAGCGCA ACGCAATTAA TGTGAGTTAG CTCACTCATT

7321 AGGCACCCCA GGCTTTACAC TTTATGCTTC CGGCTCGTAT GTTGTGTGGA ATTGTGAGCG

7381 GATAACAATT TCACACAGGA AACAGCTATG ACCATGATTA CGCCAAGCTT GCATGCCCGC

7441 GG

**Fig S14** **The sequence of pTK-PB-loc with a nucleotide length of 7442 bp**. The bolded segments marked in gray, black, dark blue, red, blue, yellow, orange and purple represent gene sequences of *P43* promoter from Bv916 (CP009611), *sgRNA,* *loc target,* *PB* promoter from *Bacillus subtilis* positive regulator (*rsbV*), *Kan^r^*, thermosensitive replication origin for *Bacillus*, *Amp^r^* and replication origin for *Escherichia coli*, respectively.

1 GGATCC**CGTA GGCGCCGACA TGCCGCCTGT TTTAGGAGAT ATTATCATCA GCGCTGACAG**

**61 AACGAAAGAG CAGGCGGAAG AGTACGGCCA TTCATTTATG AGAGAGCTCG GTTTTCTGGC**

**121 CGTGCACGGC TTTTTGCATT TGCTTGGCTA TGATCACATG ACAAAAGAAG AAGAGGAAGA**

**181 AATGTTTTCA AAGCAAAAGG ATTTGCTGGA TGAGTATGGA CTCACGAGAT CATAAAAACG**

**241 AGTGGAGCAG GTTCCTGAAG AGCTTTGTCC ATGCATGGAG GGGAATCTGG AAAACGGCGC**

**301 GGTCAGAGCG GAATTTTCAA TTTCATATTA CCGCGGCCTG CGCCGTTATC GTTTGCGGTT**

**361 TTCTCACCGG GCTGAGCACA GCCGAATGGG CCGTTGTTTT GATGCTGATC GGAGGAATGC**

**421 TTGCTTTGGA GCTGTTAAAT ACCGCGATAG AGCATGTTGT GGATTTAGTT ACCGATCAGT**

**481 ATCACCCGCT CGCAAAAGCG GCAAAGGACG CGGCCGCCGG AGCCGTTTGC GTCTTTGCCG**

**541 TGATATCGTG TATCATTGGT TTACTCATCT TCTTGCCGAA GATATGTTAG CAGAAGATTC**

**601 TTACAATTAT TTTACATTGC CAAAAATGGG CGTGAAAAAC CAATCATAAT TATGTAAAAT**

**661 AAA****GGGGACA AGTTATACTA GCCCAAAATC TCGATCTTTA TCGTTCAATT TTATTCCGAT**

**721 CAGGCAATAG TTGAACTTTT TCACCGTGGC TCAGCCACGA AAAAAA**TCTA GAGAATTCAC

781 TAGT**GCTGAC TGCGCCATTT ATCGCAATGA AGCATGCATT TCCCATTATG AAAAAACAGC**

**841 AATTCGGTCG AATCATTAAT ATGGCGTCTG TCAACGGACT GATCGGTTTT CACGGGAAAG**

**901 CGGCATATAA CAGCGCCAAG CACGGCGTCA TCGGTTTAAC GAAAGTCGGC GCACTTGAAG**

**961 GCGCGGCTGA CGGCATTACC GTCAATGCGC TTTGTCCGGG ATATGTGGAC ACTCAGCTTG**

**1021 TCCGCAATCA GTTAAAGGAC ATTTCCGCAA CAAGGAATGT ACCGTACGAG CGGGTGCTTG**

**1081 AGGACGTCAT CTTTCCGCTC GTTCCCCAAA AACGGCTGCT GTCAGTGAAG GAAATCGCTG**

**1141 ATTACGCCGT ATTTTTGGCC AGCGACAAAG CAAAAGGGGT CACAGGTCAG GCCGTAGTCA**

**1201 TGGATGGCGG GTATACCGCT CAATAGTTCT TATACTTTTT AAAAAAACAG CCGCTCTGAA**

**1261 CGGCTGTTTT TTTATGCCTG AAAACCTTGT TCACTCAATA GGATGGACTC AAAAGATCAT**

**1321 AGAAATGAGC TGAAGCGATT CTTCAAAAGC TTCGTGCATG CAGGCCGGGG CATCTGGGAA**

**1381 ACAGCGCGGA CGGAGCGGAA TTTCCAATTT CATGCCGCAG CCGCCTGTGC TGTTCTCATT**

**1441 TGCGGCTTCC TTGTAGAGCT CAGCATGATT GAGTGGATGA TTATATTCCT TTTGATAGGC**

**1501 GGTATGTTTT CGCTTGAGCT TTTAAATACA GCCATTGAAC ATACGGTTGA TTTAATAACT**

**1561 GACAAACATC ACCCTCTTGC TAAAGCGGCC AAGGACGCTG CCGCCGGGGC TGTTTGCGTT**

**1621 TTTGCCGTGA TTTCGTGTAT CATTGGTTTA CTTATTTTTT TGCCAAAGCT GTAATGGCTG**

**1681 AAAATTCTTA CATTTATTTT ACATTTTTAG AAATGGGCGT GAAAAAAAGC GCGCGATTAT**

**1741 GTAAAATATA AAGTGATAGC GGTACCATTA TAGGTAAGAG AGGAATGTAC ACAACAATCT**

**1801 TGCCTTTTTA TTTCCTGGAC AAGGGTCTCA ATTTGTAGGA ATGGGCAAAC AATTTTGGAA**

**1861 TGATTTTGTG CTCGCAAAGA GATTGTTTGA AGAAGCGAGC GATGCGATCT CCTTGGATGT**

**1921 AAAAAAACTG TGTTTTAACG GAGATATGAA TGAATTGACA AAGACAATGA ACGCGCAGCC**

**1981 CGCTA**AGATC TCTCGAGTAA CAGGTTGGCT GATAAGTCCC CGGTCTGCCA CATAGATGGC

2041 GTCGCTAGTA TTAAATGCAT ATTATTTTTA TATAGTACCA ACCTTCAAAT GATTCCCTAT

2101 AGCTTGTAAA TTCTATCATA ATTGTGGTTT CAAAATCGGC TCCGTCGATA CTATGTTATA

2161 CGCCAACTTT GAAAACAACT TTGAAAAAGC TGTTTTCTGG TATTTAAGGT TTTAGAATGC

2221 AAGGAACAGT GAATTGGAGT TCGTCTTGTT ATAATTAGCT TCTTGGGGTA TCTTTAAATA

2281 CTGTAGAAAA GAGGAAGGAA ATAATAA**ATG GCTAAAATGA GAATATCACC GGAATTGAAA**

**2341 AAACTGATCG AAAAATACCG CTGCGTAAAA GATACGGAAG GAATGTCTCC TGCTAAGGTA**

**2401 TATAAGCTGG TGGGAGAAAA TGAAAACCTA TATTTAAAAA TGACGGACAG CCGGTATAAA**

**2461 GGGACCACCT ATGATGTAGA ACGGGAAAAG GACATGATGC TATGGCTGGA AGGAAAGCTG**

**2521 CCTGTTCCAA AGGTCCTGCA CTTTGAACGG CATGATGGCT GGAGCAATCT GCTCATGAGT**

**2581 GAGGCCGATG GCGTCCTTTG CTCGGAAGAG TATGAAGATG AACAAAGCCC TGAAAAGATT**

**2641 ATCGAGCTGT ATGCGGAGTG CATCAGGCTC TTTCACTCCA TCGACATATC GGATTGTCCC**

**2701 TATACGAATA GCTTAGACAG CCGCTTAGCC GAATTGGATT ACTTACTGAA TAACGATCTG**

**2761 GCCGATGTGG ATTGCGAAAA CTGGGAAGAA GACACTCCAT TTAAAGATCC GCGCGAGCTG**

**2821 TATGATTTTT TAAAGACGGA AAAGCCCGAA GAGGAACTTG TCTTTTCCCA CGGCGACCTG**

**2881 GGAGACAGCA ACATCTTTGT GAAAGATGGC AAAGTAAGTG GCTTTATTGA TCTTGGGAGA**

**2941 AGCGGCAGGG CGGACAAGTG GTATGACATT GCCTTCTGCG TCCGGTCGAT CAGGGAGGAT**

**3001 ATCGGGGAAG AACAGTATGT CGAGCTATTT TTTGACTTAC TGGGGATCAA GCCTGATTGG**

**3061 GAGAAAATAA AATATTATAT TTTACTGGAT GAATTGTTTT AG**TACCTAGA ATTTAGATGT

3121 CTAAAAAGCT TTAACTACAA GCTTTTTAGA CATCTAATCT TTTCTGAAGT ACATCCGCAA

3181 CTGTCCATAC TCTGATGTTT TATATCTTTT CTAAAAGTTC GCTAGATAGG GGTCCCGAGC

3241 GCCTACGAGG AATTTGTATC GCCCGGG**GTC CAGAAGGTCG ATAGAAAGCG TGAGAAACAG**

**3301 CGTACAGACG ATTTAGAGAT GTAGAGGTAC TTTTATGCCG AGAAAACTTT TTGCGTGTGA**

**3361 CAGTCCTTAA AATATACTTA GAGCGTAAGC GAAAGTAGTA GCGACAGCTA TTAACTTTCG**

**3421 GTTGCAAAGC TCTAGGATTT TTAATGGACG CAGCGCATCA CACGCAAAAA GGAAATTGGA**

**3481 ATAAATGCGA AATTTGAGAT GTTAATTAAA GACCTTTTTG AGGTCTTTTT TTCTTAGATT**

**3541 TTTGGGGTTA TTTAGGGGAG AAAACATAGG GGGGTACTAC GACCTCCCCC CTAGGTGTCC**

**3601 ATTGTCCATT GTCCAAACAA ATAAATAAAT ATTGGGTTTT TAATGTTAAA AGGTTGTTTT**

**3661 TTATGTTAAA GTGAAAAAAA CAGATGTTGG GAGGTACAGT GATGGTTGTA GATAGAAAAG**

**3721 AAGAGAAAAA AGTTGCTGTT ACTTTAAGAC TTACAACAGA AGAAAATGAG ATATTAAATA**

**3781 GAATCAAAGA AAAATATAAT ATTAGCAAAT CAGATGCAAC CGGTATTCTA ATAAAAAAAT**

**3841 ATGCAAAGGA GGAATACGGT GCATTTTAAA CAAAAAAAGA TAGACAGCAC TGGCATGCTG**

**3901 CCTATCTATG ACTAAATTTT GTTAAGTGTA TTAGCACCGT TATTATATCA TGAGCGAAAA**

**3961 TGTAATAAAA GAAACTGAAA ACAAGAAAAA TTCAAGAGGA CGTAATTGGA CATTTGTTTT**

**4021 ATATCCAGAA TCAGCAAAAG CCGAGTGGTT AGAGTATTTA AAAGAGTTAC ACATTCAATT**

**4081 TGTAGTGTCT CCATTACATG ATAGGGATAC TGATACAGAA GGTAGGATGA AAAAAGAGCA**

**4141 TTATCATATT CTAGTGATGT ATGAGGGTAA TAAATCTTAT GAACAGATAA AAATAATTAA**

**4201 CAGAAGAATT GAATGCGACT ATTCCGCAGA TTGCAGGAAG TGTGAAAGGT CTTGTGAGAT**

**4261 ATATGCTTCA CATGGACGAT CCTAATAAAT TTAAATATCA AAAAGAAGAT ATGATAGTTT**

**4321 ATGGCGGTGT AGATGTTGAT GAATTATTAA AGAAAACAAC AACAGATAGA TATAAATTAA**

**4381 TTAAAGAAAT GATTGAGTTT ATTGATGAAC AAGGAATCGT AGAATTTAAG AGTTTAATGG**

**4441 ATTATGCAAT GAAGTTTAAA TTTGATGATT GGTTCCCGCT TTTATGTGAT AACTCGGCGT**

**4501 ATGTTATTCA AGAATATATA AAATCAAATC GGTATAAATC TGACCGATAG ATTTTGAATT**

**4561 TAGGTGTCAC AAGACACTCT TTTTTCGCAC CAGCGAAAAC TGGTTTAAGC CGACTGCGCA**

**4621 AAAGACATAA TCGATTCACA AAAAATAGGC ACACGAAAAA CAAGTTAAGG GATGCAGTTT**

**4681 ATGCATCCCT TAAC**GGTACC ACTGGCCGTC GTTTTACAAC GTCGTGACTG GGAAAACCCT

4741 GGCGTTACCC AACTTAATCG CCTTGCAGCA CATCCCCCTT TCGCCAGCTG GCGTAATAGC

4801 GAAGAGGCCC GCACCGATCG CCCTTCCCAA CAGTTGCGCA GCCTGAATGG CGAATGGCGC

4861 CTGATGCGGT ATTTTCTCCT TACGCATCTG TGCGGTATTT CACACCGCAT ATGGTGCACT

4921 CTCAGTACAA TCTGCTCTGA TGCCGCATAG TTAAGCCAGC CCCGACACCC GCCAACACCC

4981 GCTGACGCGC CCTGACGGGC TTGTCTGCTC CCGGCATCCG CTTACAGACA AGCTGTGACC

5041 GTCTCCGGGA GCTGCATGTG TCAGAGGTTT TCACCGTCAT CACCGAAACG CGCGAGACGA

5101 AAGGGCCTCG TGATACGCCT ATTTTTATAG GTTAATGTCA TGATAATAAT GGTTTCTTAG

5161 ACGTCAGGTG GCACTTTTCG GGGAAATGTG CGCGGAACCC CTATTTGTTT ATTTTTCTAA

5221 ATACATTCAA ATATGTATCC GCTCATGAGA CAATAACCCT GATAAATGCT TCAATAATAT

5281 TGAAAAAGGA AGAGT**ATGAG TATTCAACAT TTCCGTGTCG CCCTTATTCC CTTTTTTGCG**

**5341 GCATTTTGCC TTCCTGTTTT TGCTCACCCA GAAACGCTGG TGAAAGTAAA AGATGCTGAA**

**5401 GATCAGTTGG GTGCACGAGT GGGTTACATC GAACTGGATC TCAACAGCGG TAAGATCCTT**

**5461 GAGAGTTTTC GCCCCGAAGA ACGTTTTCCA ATGATGAGCA CTTTTAAAGT TCTGCTATGT**

**5521 GGCGCGGTAT TATCCCGTAT TGACGCCGGG CAAGAGCAAC TCGGTCGCCG CATACACTAT**

**5581 TCTCAGAATG ACTTGGTTGA GTACTCACCA GTCACAGAAA AGCATCTTAC GGATGGCATG**

**5641 ACAGTAAGAG AATTATGCAG TGCTGCCATA ACCATGAGTG ATAACACTGC GGCCAACTTA**

**5701 CTTCTGACAA CGATCGGAGG ACCGAAGGAG CTAACCGCTT TTTTGCACAA CATGGGGGAT**

**5761 CATGTAACTC GCCTTGATCG TTGGGAACCG GAGCTGAATG AAGCCATACC AAACGACGAG**

**5821 CGTGACACCA CGATGCCTGT AGCAATGGCA ACAACGTTGC GCAAACTATT AACTGGCGAA**

**5881 CTACTTACTC TAGCTTCCCG GCAACAATTA ATAGACTGGA TGGAGGCGGA TAAAGTTGCA**

**5941 GGACCACTTC TGCGCTCGGC CCTTCCGGCT GGCTGGTTTA TTGCTGATAA ATCTGGAGCC**

**6001 GGTGAGCGTG GGTCTCGCGG TATCATTGCA GCACTGGGGC CAGATGGTAA GCCCTCCCGT**

**6061 ATCGTAGTTA TCTACACGAC GGGGAGTCAG GCAACTATGG ATGAACGAAA TAGACAGATC**

**6121 GCTGAGATAG GTGCCTCACT GATTAAGCAT TGGTAA**CTGT CAGACCAAGT TTACTCATAT

6181 ATACTTTAGA TTGATTTAAA ACTTCATTTT TAATTTAAAA GGATCTAGGT GAAGATCCTT

6241 TTTGATAATC TCATGACCAA AATCCCTTAA CGTGAGTTTT CGTTCCACTG AGCGTCAGAC

6301 CCCGTAGAAA AGATCAAAGG ATCTTCTTGA GATCCTTTTT TTCTGCGCGT AATCTGCTGC

6361 TTGCAAACAA AAAAACCACC GCTACCAGCG GTGGTTTGTT TGCCGGATCA AGAGCTACCA

6421 ACTCTTTTTC CGAAGGTAAC TGGCTTCAGC AGAGCGCAGA TACCAAATAC TGTTCTTCTA

6481 GTGTAGCCGT AGTTAGGCCA CCACTTCAAG AACTCTGTAG CACCGCCTAC ATACCTCGCT

6541 CTGCTAATCC TGTTACCAGT GGCTGCTGCC AGTGGCGATA AGTCGTGTCT TACCGGGTTG

6601 GACTCAAGAC GATAGTTACC GGATAAGGCG CAGCGGTCGG GCTGAACGGG GGGTTCGTGC

6661 ACACAGCCCA GCTTGGAGCG AACGACCTAC ACCGAACTGA GATACCTACA GCGTGAGCTA

6721 TGAGAAAGCG CCACGCTTCC CGAAGGGAGA AAGGCGGACA GGTATCCGGT AAGCGGCAGG

6781 GTCGGAACAG GAGAGCGCAC GAGGGAGCTT CCAGGGGGAA ACGCCTGGTA TCTTTATAGT

6841 CCTGTCGGGT TTCGCCACCT CTGACTTGAG CGTCGATTTT TGTGATGCTC GTCAGGGGGG

6901 CGGAGCCTAT GGAAAAACGC CAGCAACGCG GCCTTTTTAC GGTTCCTGGC CTTTTGCTGG

6961 CCTTTTGCTC ACATGTTCTT TCCTGCGTTA TCCCCTGA**TT CTGTGGATAA CCGTATTACC**

**7021 GCCTTTGAGT GAGCTGATAC CGCTCGCCGC AGCCGAACGA CCGAGCGCAG CGAGTCAGTG**

**7081 AGCGAGGAAG CGGAAGA**GCG CCCAATACGC AAACCGCCTC TCCCCGCGCG TTGGCCGATT

7141 CATTAATGCA GCTGGCACGA CAGGTTTCCC GACTGGAAAG CGGGCAGTGA GCGCAACGCA

7201 ATTAATGTGA GTTAGCTCAC TCATTAGGCA CCCCAGGCTT TACACTTTAT GCTTCCGGCT

7261 CGTATGTTGT GTGGAATTGT GAGCGGATAA CAATTTCACA CAGGAAACAG CTATGACCAT

7321 GATTACGCCA AGCTTGCATG CCCGCGG

**Fig S15** **The sequence of pTK-P43-bl with a nucleotide length of 7347 bp**. The bolded segments marked in gray, black, dark blue, red, blue, yellow, orange and purple represent gene sequences of *P43* promoter from Bv916 (CP009611), *sgRNA,* *bl target,* *P43* promoter from *Bacillus subtilis* JCL16, *Kan^r^*, thermosensitive replication origin for *Bacillus*, *Amp^r^* and replication origin for *Escherichia coli*, respectively.

1 GGAT**CCCGTA GGCGCCGACA TGCCGCCTGT TTTAGGAGAT ATTATCATCA GCGCTGACAG**

**61 AACGAAAGAG CAGGCGGAAG AGTACGGCCA TTCATTTATG AGAGAGCTCG GTTTTCTGGC**

**121 CGTGCACGGC TTTTTGCATT TGCTTGGCTA TGATCACATG ACAAAAGAAG AAGAGGAAGA**

**181 AATGTTTTCA AAGCAAAAGG ATTTGCTGGA TGAGTATGGA CTCACGAGAT CATAAAAACG**

**241 AGTGGAGCAG GTTCCTGAAG AGCTTTGTCC ATGCATGGAG GGGAATCTGG AAAACGGCGC**

**301 GGTCAGAGCG GAATTTTCAA TTTCATATTA CCGCGGCCTG CGCCGTTATC GTTTGCGGTT**

**361 TTCTCACCGG GCTGAGCACA GCCGAATGGG CCGTTGTTTT GATGCTGATC GGAGGAATGC**

**421 TTGCTTTGGA GCTGTTAAAT ACCGCGATAG AGCATGTTGT GGATTTAGTT ACCGATCAGT**

**481 ATCACCCGCT CGCAAAAGCG GCAAAGGACG CGGCCGCCGG AGCCGTTTGC GTCTTTGCCG**

**541 TGATATCGTG TATCATTGGT TTACTCATCT TCTTGCCGAA GATATGTTAG CAGAAGATTC**

**601 TTACAATTAT TTTACATTGC CAAAAATGGG CGTGAAAAAC CAATCATAAT TATGTAAAAT**

**661 AAA****GAATAGG AGAATTTAAC TTTCAAAATC TCGATCTTTA TCGTTCAATT TTATTCCGAT**

**721 CAGGCAATAG TTGAACTTTT TCACCGTGGC TCAGCCACGA AAAAAA**TCTA GAGAATTCAC

781 TAGT**GAATTA TTGATCCCGT TTATGAAACT GAGCAACAAC GGGCACGCCG AAATTCTGAT**

**841 CAAGGAAATG GGAAAAGTGA AAAAAGGGGA GGGCAGCTGG GAAAAGGGCT TGGACGTGAT**

**901 GAAATCGGAG CTCAAATCGT TCGGGCTCAA TCCGGATAAA TTGATTGCAA GAGACGGATC**

**961 CGGCGTTTCT CATATTAACG GCGTATCCGC CGGCCAAATC GGAGAGCTTT TGTATGCGGT**

**1021 TCAGAAAGAA AAGTGGTACC CGGCTTTCCT TCGTTCGCTC CCTGTTGCCG GCGCAAGCGA**

**1081 CAGAATGACG GGAGGAACCC TTCGGAACCG GCTGAAGAAT ACTCCGGCAG AAGGCAAAAT**

**1141 TAAAGCGAAA ACCGGGTCAC TAACATCAGT CAGTTCGATT GCCGGCTATG CAGACACAAA**

**1201 AACAGGAGAT ACGCTTATTT TTTCCGTGCT TCAAAACGGG CTGCTTGACG AAGATGACGG**

**1261 AAAAGACATT GAAGACAAAA TCGCAGTCGT GCTGGCAAAT CAATAACATG GTCTCACTTT**

**1321 TCCACTTTTT GTCTTGTCCA CTAAAACCCT TGATTTTTCA TCTGAATAAA TGCTACTATT**

**1381 AGGACACATA ATATTAAAAG AAACCCCCAT CTATTTAGTT ATTTGTTTAG TCACTTATAA**

**1441 CTTTAACAGA TGGGGTTTTT CTGTGCAACC AATTTTAAGG GTTTTCAATA CTTTAAAACA**

**1501 CATACATACC AACACTTCAA CGCACCTTTC AGCAACTAAA ATAAAAATGA CGTTATTTCT**

**1561 ATATGTATCA AGATAAGAAA GAACAAGTTC AAAACCATCA AAAAAAGACA CCTTTTCAGG**

**1621 TGCTTTTTTT ATTTTATAAA CTCATTCCCT GATCTCGAGA ACACTGTTTA TTCTTTAACT**

**1681 CACGCACAAA GAAGAGTGTG GTTTACGGAG CTGCTCGAAC CGGGCACAAG TATCTGCAAT**

**1741 CTCGCGGCAT GCGTCAAATT CAGGGGGGAC ATCGATTTTG ACGTACTGCG CCATGCTTTA**

**1801 GATTTTTCTA TCTCGCAAAA TGATTCGCTC AGGTTTCAAC TGACAGAAGG GGACGGATCA**

**1861 GAACCCCAGC TGTATCTTGC GGGGCATCGG CCGATTTCTC TAGAGACTGT TGATTTTACT**

**1921 CATACTGATC AGGCAGAGCG GGACGCATGG ATTGACACGC AGACCCGTGT TCCGTTCAAG**

**1981 CTGTTTCATT CACCTCTGTA TCAATTTACT CTGCTT**AGAT CTCTCGAGTT AAGCCAGCCC

2041 CGACACCCGC CAACACCCGC TGACGCGCCC TGACGGGCTT GTCTGCTCCC GGCATCCGCT

2101 TACAGACAAG CTGTGACCGT CTCCGGGAGC TGCATGTGTC AGAGGTTTTC ACCGTCATCA

2161 CCGAAACGCG CGAGACGAAA GGGCCTCGTG ATACGCCTAT TTTTATAGGT TAATGTCATG

2221 ATAATAATGG TTTCTTAGAC GTCAGGTGGC ACTTTTCGGG GAAATGTGCG CGGAACCCCT

2281 ATTTGTTTAT TTTTCTAAAT ACATTCAAAT ATGTATCCGC TCATGAGACA ATAACCCTGA

2341 TAAATGCTTC AATAATAAAA AAGGATTGAT TCTAATGAAG AAAGCAGACA AGTAAGCCTC

2401 CTAAATTCAC TTTAGATAAA AATTTAGGAG GCATATCAA**A TGAACTTTAA TAAAATTGAT**

**2461 TTAGACAATT GGAAGAGAAA AGAGATATTT AATCATTATT TGAACCAACA AACGACTTTT**

**2521 AGTATAACCA CAGAAATTGA TATTAGTGTT TTATACCGAA ACATAAAACA AGAAGGATAT**

**2581 AAATTTTACC CTGCATTTAT TTTCTTAGTG ACAAGGGTGA TAAACTCAAA TACAGCTTTT**

**2641 AGAACTGGTT ACAATAGCGA CGGAGAGTTA GGTTATTGGG ATAAGTTAGA GCCACTTTAT**

**2701 ACAATTTTTG ATGGTGTATC TAAAACATTC TCTGGTATTT GGACTCCTGT AAAGAATGAC**

**2761 TTCAAAGAGT TTTATGATTT ATACCTTTCT GATGTAGAGA AATATAATGG TTCGGGGAAA**

**2821 TTGTTTCCCA AAACACCTAT ACCTGAAAAT GCTTTTTCTC TTTCTATTAT TCCATGGACT**

**2881 TCATTTACTG GGTTTAACTT AAATATCAAT AATAATAGTA ATTACCTTCT ACCCATTATT**

**2941 ACAGCAGGAA AATTCATTAA TAAAGGTAAT TCAATATATT TACCGCTATC TTTACAGGTA**

**3001 CATCATTCTG TTTGTGATGG TTATCATGCA GGATTGTTTA TGAACTCTAT TCAGGAATTG**

**3061 TCAGATAGGC CTAATGACTG GCTTTTATAA TATGAGATAA** TGCCGACTGT ACTTTTTACA

3121 GTCGGTTTTC TAATGTCACT AACCTGCCCC GTTAGTTGAA GAAGGTTTTT ATATTACAGC

3181 TCCAGATCCA TATCCTTCTT TTTCTGAACC GACTTCTCCT TTTTCGCTTC TTTATTCCAA

3241 TTGCTTTATT GACGTTGAGC CTCGGAACCC TTAACAATCC CAAAACTTGT CGAATGGTCG

3301 GCTTAATAGC TCACGCTATG CCGACATTCG TCTGCAAGTT TAGTTAAGGG TTCTTCTCAA

3361 CGCACAATAA ATTTTCTCGG CATAAATGCG TGGTCTAATT TTTATTTTTA ATAACCTTGA

3421 TAGCAAAAAA TGCCATTCCA ATACAAAACC ACATACCTAT AATCGATAAC CACATAACAG

3481 TCATAAAACC ACTCCTTTTT AACAAACTTT ATCACAAGAA ATATTTACCC GGG**GTCCAGA**

**3541 AGGTCGATAG AAAGCGTGAG AAACAGCGTA CAGACGATTT AGAGATGTAG AGGTACTTTT**

**3601 ATGCCGAGAA AACTTTTTGC GTGTGACAGT CCTTAAAATA TACTTAGAGC GTAAGCGAAA**

**3661 GTAGTAGCGA CAGCTATTAA CTTTCGGTTG CAAAGCTCTA GGATTTTTAA TGGACGCAGC**

**3721 GCATCACACG CAAAAAGGAA ATTGGAATAA ATGCGAAATT TGAGATGTTA ATTAAAGACC**

**3781 TTTTTGAGGT CTTTTTTTCT TAGATTTTTG GGGTTATTTA GGGGAGAAAA CATAGGGGGG**

**3841 TACTACGACC TCCCCCCTAG GTGTCCATTG TCCATTGTCC AAACAAATAA ATAAATATTG**

**3901 GGTTTTTAAT GTTAAAAGGT TGTTTTTTAT GTTAAAGTGA AAAAAACAGA TGTTGGGAGG**

**3961 TACAGTGATG GTTGTAGATA GAAAAGAAGA GAAAAAAGTT GCTGTTACTT TAAGACTTAC**

**4021 AACAGAAGAA AATGAGATAT TAAATAGAAT CAAAGAAAAA TATAATATTA GCAAATCAGA**

**4081 TGCAACCGGT ATTCTAATAA AAAAATATGC AAAGGAGGAA TACGGTGCAT TTTAAACAAA**

**4141 AAAAGATAGA CAGCACTGGC ATGCTGCCTA TCTATGACTA AATTTTGTTA AGTGTATTAG**

**4201 CACCGTTATT ATATCATGAG CGAAAATGTA ATAAAAGAAA CTGAAAACAA GAAAAATTCA**

**4261 AGAGGACGTA ATTGGACATT TGTTTTATAT CCAGAATCAG CAAAAGCCGA GTGGTTAGAG**

**4321 TATTTAAAAG AGTTACACAT TCAATTTGTA GTGTCTCCAT TACATGATAG GGATACTGAT**

**4381 ACAGAAGGTA GGATGAAAAA AGAGCATTAT CATATTCTAG TGATGTATGA GGGTAATAAA**

**4441 TCTTATGAAC AGATAAAAAT AATTAACAGA AGAATTGAAT GCGACTATTC CGCAGATTGC**

**4501 AGGAAGTGTG AAAGGTCTTG TGAGATATAT GCTTCACATG GACGATCCTA ATAAATTTAA**

**4561 ATATCAAAAA GAAGATATGA TAGTTTATGG CGGTGTAGAT GTTGATGAAT TATTAAAGAA**

**4621 AACAACAACA GATAGATATA AATTAATTAA AGAAATGATT GAGTTTATTG ATGAACAAGG**

**4681 AATCGTAGAA TTTAAGAGTT TAATGGATTA TGCAATGAAG TTTAAATTTG ATGATTGGTT**

**4741 CCCGCTTTTA TGTGATAACT CGGCGTATGT TATTCAAGAA TATATAAAAT CAAATCGGTA**

**4801 TAAATCTGAC CGATAGATTT TGAATTTAGG TGTCACAAGA CACTCTTTTT TCGCACCAGC**

**4861 GAAAACTGGT TTAAGCCGAC TGCGCAAAAG ACATAATCGA TTCACAAAAA ATAGGCACAC**

**4921 GAAAAACAAG TTAAGGGATG CAGTTTATGC ATCCCTTAAC** GGTACCACTG GCCGTCGTTT

4981 TACAACGTCG TGACTGGGAA AACCCTGGCG TTACCCAACT TAATCGCCTT GCAGCACATC

5041 CCCCTTTCGC CAGCTGGCGT AATAGCGAAG AGGCCCGCAC CGATCGCCCT TCCCAACAGT

5101 TGCGCAGCCT GAATGGCGAA TGGCGCCTGA TGCGGTATTT TCTCCTTACG CATCTGTGCG

5161 GTATTTCACA CCGCATATGG TGCACTCTCA GTACAATCTG CTCTGATGCC GCATAGTTAA

5221 GCCAGCCCCG ACACCCGCCA ACACCCGCTG ACGCGCCCTG ACGGGCTTGT CTGCTCCCGG

5281 CATCCGCTTA CAGACAAGCT GTGACCGTCT CCGGGAGCTG CATGTGTCAG AGGTTTTCAC

5341 CGTCATCACC GAAACGCGCG AGACGAAAGG GCCTCGTGAT ACGCCTATTT TTATAGGTTA

5401 ATGTCATGAT AATAATGGTT TCTTAGACGT CAGGTGGCAC TTTTCGGGGA AATGTGCGCG

5461 GAACCCCTAT TTGTTTATTT TTCTAAATAC ATTCAAATAT GTATCCGCTC ATGAGACAAT

5521 AACCCTGATA AATGCTTCAA TAATATTGAA AAAGGAAGAG T**ATGAGTATT CAACATTTCC**

**5581 GTGTCGCCCT TATTCCCTTT TTTGCGGCAT TTTGCCTTCC TGTTTTTGCT CACCCAGAAA**

**5641 CGCTGGTGAA AGTAAAAGAT GCTGAAGATC AGTTGGGTGC ACGAGTGGGT TACATCGAAC**

**5701 TGGATCTCAA CAGCGGTAAG ATCCTTGAGA GTTTTCGCCC CGAAGAACGT TTTCCAATGA**

**5761 TGAGCACTTT TAAAGTTCTG CTATGTGGCG CGGTATTATC CCGTATTGAC GCCGGGCAAG**

**5821 AGCAACTCGG TCGCCGCATA CACTATTCTC AGAATGACTT GGTTGAGTAC TCACCAGTCA**

**5881 CAGAAAAGCA TCTTACGGAT GGCATGACAG TAAGAGAATT ATGCAGTGCT GCCATAACCA**

**5941 TGAGTGATAA CACTGCGGCC AACTTACTTC TGACAACGAT CGGAGGACCG AAGGAGCTAA**

**6001 CCGCTTTTTT GCACAACATG GGGGATCATG TAACTCGCCT TGATCGTTGG GAACCGGAGC**

**6061 TGAATGAAGC CATACCAAAC GACGAGCGTG ACACCACGAT GCCTGTAGCA ATGGCAACAA**

**6121 CGTTGCGCAA ACTATTAACT GGCGAACTAC TTACTCTAGC TTCCCGGCAA CAATTAATAG**

**6181 ACTGGATGGA GGCGGATAAA GTTGCAGGAC CACTTCTGCG CTCGGCCCTT CCGGCTGGCT**

**6241 GGTTTATTGC TGATAAATCT GGAGCCGGTG AGCGTGGGTC TCGCGGTATC ATTGCAGCAC**

**6301 TGGGGCCAGA TGGTAAGCCC TCCCGTATCG TAGTTATCTA CACGACGGGG AGTCAGGCAA**

**6361 CTATGGATGA ACGAAATAGA CAGATCGCTG AGATAGGTGC CTCACTGATT AAGCATTGGT**

**6421 AA**CTGTCAGA CCAAGTTTAC TCATATATAC TTTAGATTGA TTTAAAACTT CATTTTTAAT

6481 TTAAAAGGAT CTAGGTGAAG ATCCTTTTTG ATAATCTCAT GACCAAAATC CCTTAACGTG

6541 AGTTTTCGTT CCACTGAGCG TCAGACCCCG TAGAAAAGAT CAAAGGATCT TCTTGAGATC

6601 CTTTTTTTCT GCGCGTAATC TGCTGCTTGC AAACAAAAAA ACCACCGCTA CCAGCGGTGG

6661 TTTGTTTGCC GGATCAAGAG CTACCAACTC TTTTTCCGAA GGTAACTGGC TTCAGCAGAG

6721 CGCAGATACC AAATACTGTT CTTCTAGTGT AGCCGTAGTT AGGCCACCAC TTCAAGAACT

6781 CTGTAGCACC GCCTACATAC CTCGCTCTGC TAATCCTGTT ACCAGTGGCT GCTGCCAGTG

6841 GCGATAAGTC GTGTCTTACC GGGTTGGACT CAAGACGATA GTTACCGGAT AAGGCGCAGC

6901 GGTCGGGCTG AACGGGGGGT TCGTGCACAC AGCCCAGCTT GGAGCGAACG ACCTACACCG

6961 AACTGAGATA CCTACAGCGT GAGCTATGAG AAAGCGCCAC GCTTCCCGAA GGGAGAAAGG

7021 CGGACAGGTA TCCGGTAAGC GGCAGGGTCG GAACAGGAGA GCGCACGAGG GAGCTTCCAG

7081 GGGGAAACGC CTGGTATCTT TATAGTCCTG TCGGGTTTCG CCACCTCTGA CTTGAGCGTC

7141 GATTTTTGTG ATGCTCGTCA GGGGGGCGGA GCCTATGGAA AAACGCCAGC AACGCGGCCT

7201 TTTTACGGTT CCTGGCCTTT TGCTGGCCTT TTGCTCACAT GTTCTTTCCT GCGTTATCCC

7261 CTGA**TTCTGT GGATAACCGT ATTACCGCCT TTGAGTGAGC TGATACCGCT CGCCGCAGCC**

**7321 GAACGACCGA GCGCAGCGAG TCAGTGAGCG AGGAAGCGGA AGA**GCGCCCA ATACGCAAAC

7381 CGCCTCTCCC CGCGCGTTGG CCGATTCATT AATGCAGCTG GCACGACAGG TTTCCCGACT

7441 GGAAAGCGGG CAGTGAGCGC AACGCAATTA ATGTGAGTTA GCTCACTCAT TAGGCACCCC

7501 AGGCTTTACA CTTTATGCTT CCGGCTCGTA TGTTGTGTGG AATTGTGAGC GGATAACAAT

7561 TTCACACAGG AAACAGCTAT GACCATGATT ACGCCAAGCT TGCATGCCCG CGG

**Fig S16** **The sequence of pTC-PrepU-fen with a nucleotide length of 7613 bp**. The bolded segments marked in gray, black, dark blue, red, green, yellow, orange and purple represent gene sequences of *P43* promoter from Bv916 (CP009611), *sgRNA,* *fen target,* *PrepU* promoter from *Staphylococcus aureus* plasmid pUB110, *Cl^r^*, thermosensitive replication origin for *Bacillus*, *Amp^r^* and replication origin for *Escherichia coli*, respectively.

1 GGATCC**CGTA GGCGCCGACA TGCCGCCTGT TTTAGGAGAT ATTATCATCA GCGCTGACAG**

**61 AACGAAAGAG CAGGCGGAAG AGTACGGCCA TTCATTTATG AGAGAGCTCG GTTTTCTGGC**

**121 CGTGCACGGC TTTTTGCATT TGCTTGGCTA TGATCACATG ACAAAAGAAG AAGAGGAAGA**

**181 AATGTTTTCA AAGCAAAAGG ATTTGCTGGA TGAGTATGGA CTCACGAGAT CATAAAAACG**

**241 AGTGGAGCAG GTTCCTGAAG AGCTTTGTCC ATGCATGGAG GGGAATCTGG AAAACGGCGC**

**301 GGTCAGAGCG GAATTTTCAA TTTCATATTA CCGCGGCCTG CGCCGTTATC GTTTGCGGTT**

**361 TTCTCACCGG GCTGAGCACA GCCGAATGGG CCGTTGTTTT GATGCTGATC GGAGGAATGC**

**421 TTGCTTTGGA GCTGTTAAAT ACCGCGATAG AGCATGTTGT GGATTTAGTT ACCGATCAGT**

**481 ATCACCCGCT CGCAAAAGCG GCAAAGGACG CGGCCGCCGG AGCCGTTTGC GTCTTTGCCG**

**541 TGATATCGTG TATCATTGGT TTACTCATCT TCTTGCCGAA GATATGTTAG CAGAAGATTC**

**601 TTACAATTAT TTTACATTGC CAAAAATGGG CGTGAAAAAC CAATCATAAT TATGTAAAAT**

**661 AAACGTTCTA CCGATACAAT AAACAAAATC TCGATCTTTA TCGTTCAATT TTATTCCGAT**

**721 CAGGCAATAG TTGAACTTTT TCACCGTGGC TCAGCCACGA AAAAAA**GAAT TC**ATGGAAAA**

**781 GAAACTTGAA GAAGTAAAAC AATTGTTATT CCGACTCGAA CTTGATATTA AAGAAACGAC**

**841 AGATTCATTA CGAAACATTA ACAAAAGCAT TGATCAACTC GATAAATACA ATTATGCAAT**

**901 GAAAATTTCG** **ATGGACTCAA AAGATCATAG AAATGAGCTG AAGCGATTCT TCAAAAGCTT**

**961 CGTGCATGCA GGCCGGGGCA TCTGGGAAAC AGCGCGGACG GAGCGGAATT TCCAATTTCA**

**1021 TGCCGCAGCC GCCTGTGCTG TTCTCATTTG CGGCTTCCTT GTAGAGCTCA GCATGATTGA**

**1081 GTGGATGATT ATATTCCTTT TGATAGGCGG TATGTTTTCG CTTGAGCTTT TAAATACAGC**

**1141 CATTGAACAT ACGGTTGATT TAATAACTGA CAAACATCAC CCTCTTGCTA AAGCGGCCAA**

**1201 GGACGCTGCC GCCGGGGCTG TTTGCGTTTT TGCCGTGATT TCGTGTATCA TTGGTTTACT**

**1261 TATTTTTTTG CCAAAGCTGT AATGGCTGAA AATTCTTACA TTTATTTTAC ATTTTTAGAA**

**1321 ATGGGCGTGA AAAAAAGCGC GCGATTATGT AAAATATAAA GTGATAGCGG TACCATTATA**

**1381 GGTAAGAGAG GAATGTACAC** **AAGGAGATTG TGGAGCAAAA CATATTTAAC GAGGATTTGT**

**1441 CACAACTTCT TTACTCATTT ATTGATTCTA AAGAAACATT CTCCTTTGCT GAATCCACAA**

**1501 TCTTACATTA TGTTGTTTTT GGAGGGGAGA ATTTAGATGT TGCAACAAGG CTTGGCGCGG**

**1561 GAATAGAAAT TCTTATTCTT TCATCAGATA TTATGGAT**CT CGAGTAACAG GTTGGCTGAT

1621 AAGTCCCCGG TCTGCCACAT AGATGGCGTC GCTAGTATTA AATGCATATT ATTTTTATAT

1681 AGTACCAACC TTCAAATGAT TCCCTATAGC TTGTAAATTC TATCATAATT GTGGTTTCAA

1741 AATCGGCTCC GTCGATACTA TGTTATACGC CAACTTTGAA AACAACTTTG AAAAAGCTGT

1801 TTTCTGGTAT TTAAGGTTTT AGAATGCAAG GAACAGTGAA TTGGAGTTCG TCTTGTTATA

1861 ATTAGCTTCT TGGGGTATCT TTAAATACTG TAGAAAAGAG GAAGGAAATA ATAA**ATGGCT**

**1921 AAAATGAGAA TATCACCGGA ATTGAAAAAA CTGATCGAAA AATACCGCTG CGTAAAAGAT**

**1981 ACGGAAGGAA TGTCTCCTGC TAAGGTATAT AAGCTGGTGG GAGAAAATGA AAACCTATAT**

**2041 TTAAAAATGA CGGACAGCCG GTATAAAGGG ACCACCTATG ATGTAGAACG GGAAAAGGAC**

**2101 ATGATGCTAT GGCTGGAAGG AAAGCTGCCT GTTCCAAAGG TCCTGCACTT TGAACGGCAT**

**2161 GATGGCTGGA GCAATCTGCT CATGAGTGAG GCCGATGGCG TCCTTTGCTC GGAAGAGTAT**

**2221 GAAGATGAAC AAAGCCCTGA AAAGATTATC GAGCTGTATG CGGAGTGCAT CAGGCTCTTT**

**2281 CACTCCATCG ACATATCGGA TTGTCCCTAT ACGAATAGCT TAGACAGCCG CTTAGCCGAA**

**2341 TTGGATTACT TACTGAATAA CGATCTGGCC GATGTGGATT GCGAAAACTG GGAAGAAGAC**

**2401 ACTCCATTTA AAGATCCGCG CGAGCTGTAT GATTTTTTAA AGACGGAAAA GCCCGAAGAG**

**2461 GAACTTGTCT TTTCCCACGG CGACCTGGGA GACAGCAACA TCTTTGTGAA AGATGGCAAA**

**2521 GTAAGTGGCT TTATTGATCT TGGGAGAAGC GGCAGGGCGG ACAAGTGGTA TGACATTGCC**

**2581 TTCTGCGTCC GGTCGATCAG GGAGGATATC GGGGAAGAAC AGTATGTCGA GCTATTTTTT**

**2641 GACTTACTGG GGATCAAGCC TGATTGGGAG AAAATAAAAT ATTATATTTT ACTGGATGAA**

**2701 TTGTTTTAG**T ACCTAGAATT TAGATGTCTA AAAAGCTTTA ACTACAAGCT TTTTAGACAT

2761 CTAATCTTTT CTGAAGTACA TCCGCAACTG TCCATACTCT GATGTTTTAT ATCTTTTCTA

2821 AAAGTTCGCT AGATAGGGGT CCCGAGCGCC TACGAGGAAT TTGTATCGCC CGGG **GTCCAG**

**2881 AAGGTCGATA GAAAGCGTGA GAAACAGCGT ACAGACGATT TAGAGATGTA GAGGTACTTT**

**2941 TATGCCGAGA AAACTTTTTG CGTGTGACAG TCCTTAAAAT ATACTTAGAG CGTAAGCGAA**

**3001 AGTAGTAGCG ACAGCTATTA ACTTTCGGTT GCAAAGCTCT AGGATTTTTA ATGGACGCAG**

**3061 CGCATCACAC GCAAAAAGGA AATTGGAATA AATGCGAAAT TTGAGATGTT AATTAAAGAC**

**3121 CTTTTTGAGG TCTTTTTTTC TTAGATTTTT GGGGTTATTT AGGGGAGAAA ACATAGGGGG**

**3181 GTACTACGAC CTCCCCCCTA GGTGTCCATT GTCCATTGTC CAAACAAATA AATAAATATT**

**3241 GGGTTTTTAA TGTTAAAAGG TTGTTTTTTA TGTTAAAGTG AAAAAAACAG ATGTTGGGAG**

**3301 GTACAGTGAT GGTTGTAGAT AGAAAAGAAG AGAAAAAAGT TGCTGTTACT TTAAGACTTA**

**3361 CAACAGAAGA AAATGAGATA TTAAATAGAA TCAAAGAAAA ATATAATATT AGCAAATCAG**

**3421 ATGCAACCGG TATTCTAATA AAAAAATATG CAAAGGAGGA ATACGGTGCA TTTTAAACAA**

**3481 AAAAAGATAG ACAGCACTGG CATGCTGCCT ATCTATGACT AAATTTTGTT AAGTGTATTA**

**3541 GCACCGTTAT TATATCATGA GCGAAAATGT AATAAAAGAA ACTGAAAACA AGAAAAATTC**

**3601 AAGAGGACGT AATTGGACAT TTGTTTTATA TCCAGAATCA GCAAAAGCCG AGTGGTTAGA**

**3661 GTATTTAAAA GAGTTACACA TTCAATTTGT AGTGTCTCCA TTACATGATA GGGATACTGA**

**3721 TACAGAAGGT AGGATGAAAA AAGAGCATTA TCATATTCTA GTGATGTATG AGGGTAATAA**

**3781 ATCTTATGAA CAGATAAAAA TAATTAACAG AAGAATTGAA TGCGACTATT CCGCAGATTG**

**3841 CAGGAAGTGT GAAAGGTCTT GTGAGATATA TGCTTCACAT GGACGATCCT AATAAATTTA**

**3901 AATATCAAAA AGAAGATATG ATAGTTTATG GCGGTGTAGA TGTTGATGAA TTATTAAAGA**

**3961 AAACAACAAC AGATAGATAT AAATTAATTA AAGAAATGAT TGAGTTTATT GATGAACAAG**

**4021 GAATCGTAGA ATTTAAGAGT TTAATGGATT ATGCAATGAA GTTTAAATTT GATGATTGGT**

**4081 TCCCGCTTTT ATGTGATAAC TCGGCGTATG TTATTCAAGA ATATATAAAA TCAAATCGGT**

**4141 ATAAATCTGA CCGATAGATT TTGAATTTAG GTGTCACAAG ACACTCTTTT TTCGCACCAG**

**4201 CGAAAACTGG TTTAAGCCGA CTGCGCAAAA GACATAATCG ATTCACAAAA AATAGGCACA**

**4261 CGAAAAACAA GTTAAGGGAT GCAGTTTATG CATCCCTTAA C**GGTACCACT GGCCGTCGTT

4321 TTACAACGTC GTGACTGGGA AAACCCTGGC GTTACCCAAC TTAATCGCCT TGCAGCACAT

4381 CCCCCTTTCG CCAGCTGGCG TAATAGCGAA GAGGCCCGCA CCGATCGCCC TTCCCAACAG

4441 TTGCGCAGCC TGAATGGCGA ATGGCGCCTG ATGCGGTATT TTCTCCTTAC GCATCTGTGC

4501 GGTATTTCAC ACCGCATATG GTGCACTCTC AGTACAATCT GCTCTGATGC CGCATAGTTA

4561 AGCCAGCCCC GACACCCGCC AACACCCGCT GACGCGCCCT GACGGGCTTG TCTGCTCCCG

4621 GCATCCGCTT ACAGACAAGC TGTGACCGTC TCCGGGAGCT GCATGTGTCA GAGGTTTTCA

4681 CCGTCATCAC CGAAACGCGC GAGACGAAAG GGCCTCGTGA TACGCCTATT TTTATAGGTT

4741 AATGTCATGA TAATAATGGT TTCTTAGACG TCAGGTGGCA CTTTTCGGGG AAATGTGCGC

4801 GGAACCCCTA TTTGTTTATT TTTCTAAATA CATTCAAATA TGTATCCGCT CATGAGACAA

4861 TAACCCTGAT AAATGCTTCA ATAATATTGA AAAAGGAAGA GT**ATGAGTAT TCAACATTTC**

**4921 CGTGTCGCCC TTATTCCCTT TTTTGCGGCA TTTTGCCTTC CTGTTTTTGC TCACCCAGAA**

**4981 ACGCTGGTGA AAGTAAAAGA TGCTGAAGAT CAGTTGGGTG CACGAGTGGG TTACATCGAA**

**5041 CTGGATCTCA ACAGCGGTAA GATCCTTGAG AGTTTTCGCC CCGAAGAACG TTTTCCAATG**

**5101 ATGAGCACTT TTAAAGTTCT GCTATGTGGC GCGGTATTAT CCCGTATTGA CGCCGGGCAA**

**5161 GAGCAACTCG GTCGCCGCAT ACACTATTCT CAGAATGACT TGGTTGAGTA CTCACCAGTC**

**5221 ACAGAAAAGC ATCTTACGGA TGGCATGACA GTAAGAGAAT TATGCAGTGC TGCCATAACC**

**5281 ATGAGTGATA ACACTGCGGC CAACTTACTT CTGACAACGA TCGGAGGACC GAAGGAGCTA**

**5341 ACCGCTTTTT TGCACAACAT GGGGGATCAT GTAACTCGCC TTGATCGTTG GGAACCGGAG**

**5401 CTGAATGAAG CCATACCAAA CGACGAGCGT GACACCACGA TGCCTGTAGC AATGGCAACA**

**5461 ACGTTGCGCA AACTATTAAC TGGCGAACTA CTTACTCTAG CTTCCCGGCA ACAATTAATA**

**5521 GACTGGATGG AGGCGGATAA AGTTGCAGGA CCACTTCTGC GCTCGGCCCT TCCGGCTGGC**

**5581 TGGTTTATTG CTGATAAATC TGGAGCCGGT GAGCGTGGGT CTCGCGGTAT CATTGCAGCA**

**5641 CTGGGGCCAG ATGGTAAGCC CTCCCGTATC GTAGTTATCT ACACGACGGG GAGTCAGGCA**

**5701 ACTATGGATG AACGAAATAG ACAGATCGCT GAGATAGGTG CCTCACTGAT TAAGCATTGG**

**5761 TAA**CTGTCAG ACCAAGTTTA CTCATATATA CTTTAGATTG ATTTAAAACT TCATTTTTAA

5821 TTTAAAAGGA TCTAGGTGAA GATCCTTTTT GATAATCTCA TGACCAAAAT CCCTTAACGT

5881 GAGTTTTCGT TCCACTGAGC GTCAGACCCC GTAGAAAAGA TCAAAGGATC TTCTTGAGAT

5941 CCTTTTTTTC TGCGCGTAAT CTGCTGCTTG CAAACAAAAA AACCACCGCT ACCAGCGGTG

6001 GTTTGTTTGC CGGATCAAGA GCTACCAACT CTTTTTCCGA AGGTAACTGG CTTCAGCAGA

6061 GCGCAGATAC CAAATACTGT TCTTCTAGTG TAGCCGTAGT TAGGCCACCA CTTCAAGAAC

6121 TCTGTAGCAC CGCCTACATA CCTCGCTCTG CTAATCCTGT TACCAGTGGC TGCTGCCAGT

6181 GGCGATAAGT CGTGTCTTAC CGGGTTGGAC TCAAGACGAT AGTTACCGGA TAAGGCGCAG

6241 CGGTCGGGCT GAACGGGGGG TTCGTGCACA CAGCCCAGCT TGGAGCGAAC GACCTACACC

6301 GAACTGAGAT ACCTACAGCG TGAGCTATGA GAAAGCGCCA CGCTTCCCGA AGGGAGAAAG

6361 GCGGACAGGT ATCCGGTAAG CGGCAGGGTC GGAACAGGAG AGCGCACGAG GGAGCTTCCA

6421 GGGGGAAACG CCTGGTATCT TTATAGTCCT GTCGGGTTTC GCCACCTCTG ACTTGAGCGT

6481 CGATTTTTGT GATGCTCGTC AGGGGGGCGG AGCCTATGGA AAAACGCCAG CAACGCGGCC

6541 TTTTTACGGT TCCTGGCCTT TTGCTGGCCT TTTGCTCACA TGTTCTTTCC TGCGTTATCC

6601 CCTGA**TTCTG TGGATAACCG TATTACCGCC TTTGAGTGAG CTGATACCGC TCGCCGCAGC**

**6661 CGAACGACCG AGCGCAGCGA GTCAGTGAGC GAGGAAGCGG AAGA**GCGCCC AATACGCAAA

6721 CCGCCTCTCC CCGCGCGTTG GCCGATTCAT TAATGCAGCT GGCACGACAG GTTTCCCGAC

6781 TGGAAAGCGG GCAGTGAGCG CAACGCAATT AATGTGAGTT AGCTCACTCA TTAGGCACCC

6841 CAGGCTTTAC ACTTTATGCT TCCGGCTCGT ATGTTGTGTG GAATTGTGAG CGGATAACAA

6901 TTTCACACAG GAAACAGCTA TGACCATGAT TACGCCAAGC TTGCATGCCC GCGG

**Fig S17** **The sequence of pTK-comX with a nucleotide length of 6954 bp**. The bolded segments marked in gray, black, dark blue, red, blue, yellow, orange and purple represent gene sequences of *P43* promoter from Bv916 (CP009611), *sgRNA,* *comX target,* *P43* promoter from *Bacillus subtilis* JCL16, *Kan^r^*, thermosensitive replication origin for *Bacillus*, *Amp^r^* and replication origin for *Escherichia coli*, respectively.

1 GGATCC**CGTA GGCGCCGACA TGCCGCCTGT TTTAGGAGAT ATTATCATCA GCGCTGACAG**

**61 AACGAAAGAG CAGGCGGAAG AGTACGGCCA TTCATTTATG AGAGAGCTCG GTTTTCTGGC**

**121 CGTGCACGGC TTTTTGCATT TGCTTGGCTA TGATCACATG ACAAAAGAAG AAGAGGAAGA**

**181 AATGTTTTCA AAGCAAAAGG ATTTGCTGGA TGAGTATGGA CTCACGAGAT CATAAAAACG**

**241 AGTGGAGCAG GTTCCTGAAG AGCTTTGTCC ATGCATGGAG GGGAATCTGG AAAACGGCGC**

**301 GGTCAGAGCG GAATTTTCAA TTTCATATTA CCGCGGCCTG CGCCGTTATC GTTTGCGGTT**

**361 TTCTCACCGG GCTGAGCACA GCCGAATGGG CCGTTGTTTT GATGCTGATC GGAGGAATGC**

**421 TTGCTTTGGA GCTGTTAAAT ACCGCGATAG AGCATGTTGT GGATTTAGTT ACCGATCAGT**

**481 ATCACCCGCT CGCAAAAGCG GCAAAGGACG CGGCCGCCGG AGCCGTTTGC GTCTTTGCCG**

**541 TGATATCGTG TATCATTGGT TTACTCATCT TCTTGCCGAA GATATGTTAG CAGAAGATTC**

**601 TTACAATTAT TTTACATTGC CAAAAATGGG CGTGAAAAAC CAATCATAAT TATGTAAAAT**

**661 AAA****ATATGCG TTCGCTTTTT TCTCAAAATC TCGATCTTTA TCGTTCAATT TTATTCCGAT**

**721 CAGGCAATAG TTGAACTTTT TCACCGTGGC TCAGCCACGA AAAAAA**TCTA GAGAATTC**GT**

**781 CAGCAAGGAG TGCGCATCCG AACTGGCTAA GGGTGTTCAA AAACTCACTG GCAGCGATAT**

**841 CGGCATTAGC TTTACTGGTG TAGCAGGACC TGATGCTCAA GAAGGGCATG AGCCTGGGCA**

**901 TGTGTTTATC GGCATTTCCG CAAATGGTAA AGAAGAGGTT CACGAGTTTC ACTTTGCGGG**

**961 CTCCAGAACG GGGATCAGAA AACGCGGCGC TAAATACGGC TGCCATTTAA TCTTAAAGCT**

**1021 TTTAGAGCAA AAACATGGTC TCACTTTTCC ACTTTTTGTC TTGTCCACTA AAACCCTTGA**

**1081 TTTTTCATCT GAATAAATGC TACTATTAGG ACACATAATA TTAAAAGAAA CCCCCATCTA**

**1141 TTTAGTTATT TGTTTAGTCA CTTATAACTT TAACAGATGG GGTTTTTCTG TGCAACCAAT**

**1201 TTTAAGGGTT TTCAATACTT TAAAACACAT ACATACCAAC ACTTCAACGC ACCTTTCAGC**

**1261 AACTAAAATA AAAATGACGT TATTTCTATA TGTATCAAGA TAAGAAAGAA CAAGTTCAAA**

**1321 ACCATCAAAA AAAGACACCT TTTCAGGTGC TTTTTTTATT TTATAAACTC ATTCCCTGAT**

**1381 CTCAGTGATC GTCAGGCAGC CTTAGATATG GCTCTTAAAC AAATAGAAAA ACAGTTCGGC**

**1441 AAAGGTTCCA TTATGAAACT GGGAGAAAAG ACAGATACAA GAATTTCTAC TGTACCAAGC**

**1501 GGCTCCCTCG CTCTTGATAC AGCACTGGGA ATTGGCGGAT ATCCTCGCGG ACGGATTATT**

**1561 GAAGTATACG GTCCTGAAAG CTCAGGTAAA ACAACTGTGG CGCTTCATGC GATTGCTGAA**

**1621 GTTCAGCAGC AGGGCGGACA AGCCGCGTTT ATCGATGCGG AGCATGCGTT AGATCCGGTA**

**1681 TACGCGCAAA AGCT**CTCGAG TTAAGCCAGC CCCGACACCC GCCAACACCC GCTGACGCGC

1741 CCTGACGGGC TTGTCTGCTC CCGGCATCCG CTTACAGACA AGCTGTGACC GTCTCCGGGA

1801 GCTGCATGTG TCAGAGGTTT TCACCGTCAT CACCGAAACG CGCGAGACGA AAGGGCCTCG

1861 TGATACGCCT ATTTTTATAG GTTAATGTCA TGATAATAAT GGTTTCTTAG ACGTCAGGTG

1921 GCACTTTTCG GGGAAATGTG CGCGGAACCC CTATTTGTTT ATTTTTCTAA ATACATTCAA

1981 ATATGTATCC GCTCATGAGA CAATAACCCT GATAAATGCT TCAATAATAA AAAAGGATTG

2041 ATTCTAATGA AGAAAGCAGA CAAGTAAGCC TCCTAAATTC ACTTTAGATA AAAATTTAGG

2101 AGGCATATCA A**ATGAACTTT AATAAAATTG ATTTAGACAA TTGGAAGAGA AAAGAGATAT**

**2161 TTAATCATTA TTTGAACCAA CAAACGACTT TTAGTATAAC CACAGAAATT GATATTAGTG**

**2221 TTTTATACCG AAACATAAAA CAAGAAGGAT ATAAATTTTA CCCTGCATTT ATTTTCTTAG**

**2281 TGACAAGGGT GATAAACTCA AATACAGCTT TTAGAACTGG TTACAATAGC GACGGAGAGT**

**2341 TAGGTTATTG GGATAAGTTA GAGCCACTTT ATACAATTTT TGATGGTGTA TCTAAAACAT**

**2401 TCTCTGGTAT TTGGACTCCT GTAAAGAATG ACTTCAAAGA GTTTTATGAT TTATACCTTT**

**2461 CTGATGTAGA GAAATATAAT GGTTCGGGGA AATTGTTTCC CAAAACACCT ATACCTGAAA**

**2521 ATGCTTTTTC TCTTTCTATT ATTCCATGGA CTTCATTTAC TGGGTTTAAC TTAAATATCA**

**2581 ATAATAATAG TAATTACCTT CTACCCATTA TTACAGCAGG AAAATTCATT AATAAAGGTA**

**2641 ATTCAATATA TTTACCGCTA TCTTTACAGG TACATCATTC TGTTTGTGAT GGTTATCATG**

**2701 CAGGATTGTT TATGAACTCT ATTCAGGAAT TGTCAGATAG GCCTAATGAC TGGCTTTTAT**

**2761 AATATGAGAT AA**TGCCGACT GTACTTTTTA CAGTCGGTTT TCTAATGTCA CTAACCTGCC

2821 CCGTTAGTTG AAGAAGGTTT TTATATTACA GCTCCAGATC CATATCCTTC TTTTTCTGAA

2881 CCGACTTCTC CTTTTTCGCT TCTTTATTCC AATTGCTTTA TTGACGTTGA GCCTCGGAAC

2941 CCTTAACAAT CCCAAAACTT GTCGAATGGT CGGCTTAATA GCTCACGCTA TGCCGACATT

3001 CGTCTGCAAG TTTAGTTAAG GGTTCTTCTC AACGCACAAT AAATTTTCTC GGCATAAATG

3061 CGTGGTCTAA TTTTTATTTT TAATAACCTT GATAGCAAAA AATGCCATTC CAATACAAAA

3121 CCACATACCT ATAATCGATA ACCACATAAC AGTCATAAAA CCACTCCTTT TTAACAAACT

3181 TTATCACAAG AAATATTTAC CCGGG**GTCCA GAAGGTCGAT AGAAAGCGTG AGAAACAGCG**

**3241 TACAGACGAT TTAGAGATGT AGAGGTACTT TTATGCCGAG AAAACTTTTT GCGTGTGACA**

**3301 GTCCTTAAAA TATACTTAGA GCGTAAGCGA AAGTAGTAGC GACAGCTATT AACTTTCGGT**

**3361 TGCAAAGCTC TAGGATTTTT AATGGACGCA GCGCATCACA CGCAAAAAGG AAATTGGAAT**

**3421 AAATGCGAAA TTTGAGATGT TAATTAAAGA CCTTTTTGAG GTCTTTTTTT CTTAGATTTT**

**3481 TGGGGTTATT TAGGGGAGAA AACATAGGGG GGTACTACGA CCTCCCCCCT AGGTGTCCAT**

**3541 TGTCCATTGT CCAAACAAAT AAATAAATAT TGGGTTTTTA ATGTTAAAAG GTTGTTTTTT**

**3601 ATGTTAAAGT GAAAAAAACA GATGTTGGGA GGTACAGTGA TGGTTGTAGA TAGAAAAGAA**

**3661 GAGAAAAAAG TTGCTGTTAC TTTAAGACTT ACAACAGAAG AAAATGAGAT ATTAAATAGA**

**3721 ATCAAAGAAA AATATAATAT TAGCAAATCA GATGCAACCG GTATTCTAAT AAAAAAATAT**

**3781 GCAAAGGAGG AATACGGTGC ATTTTAAACA AAAAAAGATA GACAGCACTG GCATGCTGCC**

**3841 TATCTATGAC TAAATTTTGT TAAGTGTATT AGCACCGTTA TTATATCATG AGCGAAAATG**

**3901 TAATAAAAGA AACTGAAAAC AAGAAAAATT CAAGAGGACG TAATTGGACA TTTGTTTTAT**

**3961 ATCCAGAATC AGCAAAAGCC GAGTGGTTAG AGTATTTAAA AGAGTTACAC ATTCAATTTG**

**4021 TAGTGTCTCC ATTACATGAT AGGGATACTG ATACAGAAGG TAGGATGAAA AAAGAGCATT**

**4081 ATCATATTCT AGTGATGTAT GAGGGTAATA AATCTTATGA ACAGATAAAA ATAATTAACA**

**4141 GAAGAATTGA ATGCGACTAT TCCGCAGATT GCAGGAAGTG TGAAAGGTCT TGTGAGATAT**

**4201 ATGCTTCACA TGGACGATCC TAATAAATTT AAATATCAAA AAGAAGATAT GATAGTTTAT**

**4261 GGCGGTGTAG ATGTTGATGA ATTATTAAAG AAAACAACAA CAGATAGATA TAAATTAATT**

**4321 AAAGAAATGA TTGAGTTTAT TGATGAACAA GGAATCGTAG AATTTAAGAG TTTAATGGAT**

**4381 TATGCAATGA AGTTTAAATT TGATGATTGG TTCCCGCTTT TATGTGATAA CTCGGCGTAT**

**4441 GTTATTCAAG AATATATAAA ATCAAATCGG TATAAATCTG ACCGATAGAT TTTGAATTTA**

**4501 GGTGTCACAA GACACTCTTT TTTCGCACCA GCGAAAACTG GTTTAAGCCG ACTGCGCAAA**

**4561 AGACATAATC GATTCACAAA AAATAGGCAC ACGAAAAACA AGTTAAGGGA TGCAGTTTAT**

**4621 GCATCCCTTA AC**GGTACCAC TGGCCGTCGT TTTACAACGT CGTGACTGGG AAAACCCTGG

4681 CGTTACCCAA CTTAATCGCC TTGCAGCACA TCCCCCTTTC GCCAGCTGGC GTAATAGCGA

4741 AGAGGCCCGC ACCGATCGCC CTTCCCAACA GTTGCGCAGC CTGAATGGCG AATGGCGCCT

4801 GATGCGGTAT TTTCTCCTTA CGCATCTGTG CGGTATTTCA CACCGCATAT GGTGCACTCT

4861 CAGTACAATC TGCTCTGATG CCGCATAGTT AAGCCAGCCC CGACACCCGC CAACACCCGC

4921 TGACGCGCCC TGACGGGCTT GTCTGCTCCC GGCATCCGCT TACAGACAAG CTGTGACCGT

4981 CTCCGGGAGC TGCATGTGTC AGAGGTTTTC ACCGTCATCA CCGAAACGCG CGAGACGAAA

5041 GGGCCTCGTG ATACGCCTAT TTTTATAGGT TAATGTCATG ATAATAATGG TTTCTTAGAC

5101 GTCAGGTGGC ACTTTTCGGG GAAATGTGCG CGGAACCCCT ATTTGTTTAT TTTTCTAAAT

5161 ACATTCAAAT ATGTATCCGC TCATGAGACA ATAACCCTGA TAAATGCTTC AATAATATTG

5221 AAAAAGGAAG AGT**ATGAGTA TTCAACATTT CCGTGTCGCC CTTATTCCCT TTTTTGCGGC**

**5281 ATTTTGCCTT CCTGTTTTTG CTCACCCAGA AACGCTGGTG AAAGTAAAAG ATGCTGAAGA**

**5341 TCAGTTGGGT GCACGAGTGG GTTACATCGA ACTGGATCTC AACAGCGGTA AGATCCTTGA**

**5401 GAGTTTTCGC CCCGAAGAAC GTTTTCCAAT GATGAGCACT TTTAAAGTTC TGCTATGTGG**

**5461 CGCGGTATTA TCCCGTATTG ACGCCGGGCA AGAGCAACTC GGTCGCCGCA TACACTATTC**

**5521 TCAGAATGAC TTGGTTGAGT ACTCACCAGT CACAGAAAAG CATCTTACGG ATGGCATGAC**

**5581 AGTAAGAGAA TTATGCAGTG CTGCCATAAC CATGAGTGAT AACACTGCGG CCAACTTACT**

**5641 TCTGACAACG ATCGGAGGAC CGAAGGAGCT AACCGCTTTT TTGCACAACA TGGGGGATCA**

**5701 TGTAACTCGC CTTGATCGTT GGGAACCGGA GCTGAATGAA GCCATACCAA ACGACGAGCG**

**5761 TGACACCACG ATGCCTGTAG CAATGGCAAC AACGTTGCGC AAACTATTAA CTGGCGAACT**

**5821 ACTTACTCTA GCTTCCCGGC AACAATTAAT AGACTGGATG GAGGCGGATA AAGTTGCAGG**

**5881 ACCACTTCTG CGCTCGGCCC TTCCGGCTGG CTGGTTTATT GCTGATAAAT CTGGAGCCGG**

**5941 TGAGCGTGGG TCTCGCGGTA TCATTGCAGC ACTGGGGCCA GATGGTAAGC CCTCCCGTAT**

**6001 CGTAGTTATC TACACGACGG GGAGTCAGGC AACTATGGAT GAACGAAATA GACAGATCGC**

**6061 TGAGATAGGT GCCTCACTGA TTAAGCATTG GTAA**CTGTCA GACCAAGTTT ACTCATATAT

6121 ACTTTAGATT GATTTAAAAC TTCATTTTTA ATTTAAAAGG ATCTAGGTGA AGATCCTTTT

6181 TGATAATCTC ATGACCAAAA TCCCTTAACG TGAGTTTTCG TTCCACTGAG CGTCAGACCC

6241 CGTAGAAAAG ATCAAAGGAT CTTCTTGAGA TCCTTTTTTT CTGCGCGTAA TCTGCTGCTT

6301 GCAAACAAAA AAACCACCGC TACCAGCGGT GGTTTGTTTG CCGGATCAAG AGCTACCAAC

6361 TCTTTTTCCG AAGGTAACTG GCTTCAGCAG AGCGCAGATA CCAAATACTG TTCTTCTAGT

6421 GTAGCCGTAG TTAGGCCACC ACTTCAAGAA CTCTGTAGCA CCGCCTACAT ACCTCGCTCT

6481 GCTAATCCTG TTACCAGTGG CTGCTGCCAG TGGCGATAAG TCGTGTCTTA CCGGGTTGGA

6541 CTCAAGACGA TAGTTACCGG ATAAGGCGCA GCGGTCGGGC TGAACGGGGG GTTCGTGCAC

6601 ACAGCCCAGC TTGGAGCGAA CGACCTACAC CGAACTGAGA TACCTACAGC GTGAGCTATG

6661 AGAAAGCGCC ACGCTTCCCG AAGGGAGAAA GGCGGACAGG TATCCGGTAA GCGGCAGGGT

6721 CGGAACAGGA GAGCGCACGA GGGAGCTTCC AGGGGGAAAC GCCTGGTATC TTTATAGTCC

6781 TGTCGGGTTT CGCCACCTCT GACTTGAGCG TCGATTTTTG TGATGCTCGT CAGGGGGGCG

6841 GAGCCTATGG AAAAACGCCA GCAACGCGGC CTTTTTACGG TTCCTGGCCT TTTGCTGGCC

6901 TTTTGCTCAC ATGTTCTTTC CTGCGTTATC CCCTGA**TTCT GTGGATAACC GTATTACCGC**

**6961 CTTTGAGTGA GCTGATACCG CTCGCCGCAG CCGAACGACC GAGCGCAGCG AGTCAGTGAG**

**7021 CGAGGAAGCG GAAGA**GCGCC CAATACGCAA ACCGCCTCTC CCCGCGCGTT GGCCGATTCA

7081 TTAATGCAGC TGGCACGACA GGTTTCCCGA CTGGAAAGCG GGCAGTGAGC GCAACGCAAT

7141 TAATGTGAGT TAGCTCACTC ATTAGGCACC CCAGGCTTTA CACTTTATGC TTCCGGCTCG

7201 TATGTTGTGT GGAATTGTGA GCGGATAACA ATTTCACACA GGAAACAGCT ATGACCATGA

7261 TTACGCCAAG CTTGCATGCC CGCGG

**Fig S18** **The sequence of pTC-recA with a nucleotide length of 7285 bp**. The bolded segments marked in gray, black, dark blue, red, green, yellow, orange and purple represent gene sequences of *P43* promoter from Bv916 (CP009611), *sgRNA,* *ComX target,* *PrepU* promoter from *Staphylococcus aureus* plasmid pUB110, *Cl^r^*, thermosensitive replication origin for *Bacillus*, *Amp^r^* and replication origin for *Escherichia coli*, respectively.
